# Supplementary material for: An insulin receptor activity surge in follicle cells drives vitellogenesis by upregulating CrebA
Source: EMBO Rep. 2026 Jan 3;27(3):748–73. doi: 10.1038/s44319-025-00672-6 (PMC12894986; doi:10.1038/s44319-025-00672-6)
Supplement: Supplementary file 1 — Appendix [file 44319_2025_672_MOESM1_ESM.pdf]

**Appendix for "An insulin receptor activity surge in follicle cells drives vitellogenesis by upregulating CrebA"**

**Table of Contents**


| | | |
|---|---|---|
| Appendix Figure S1 | Reproducibility of ovarian scRNA-seq data | Page 2 |
| Appendix Figure S2 | Fat-body knockdown of *CrebA* has little effect on female fecundity | Page 3-4 |
| Appendix Figure S3 | The very few eggs deposited by *tj>CrebA*$^{RNAi\text{-}2}$ females show eggshell deformation | Page 5 |
| Appendix Figure S4 | Treatment of cultured *Drosophila* ovaries with human insulin increased the *CrebA* mRNA | Page 6 |

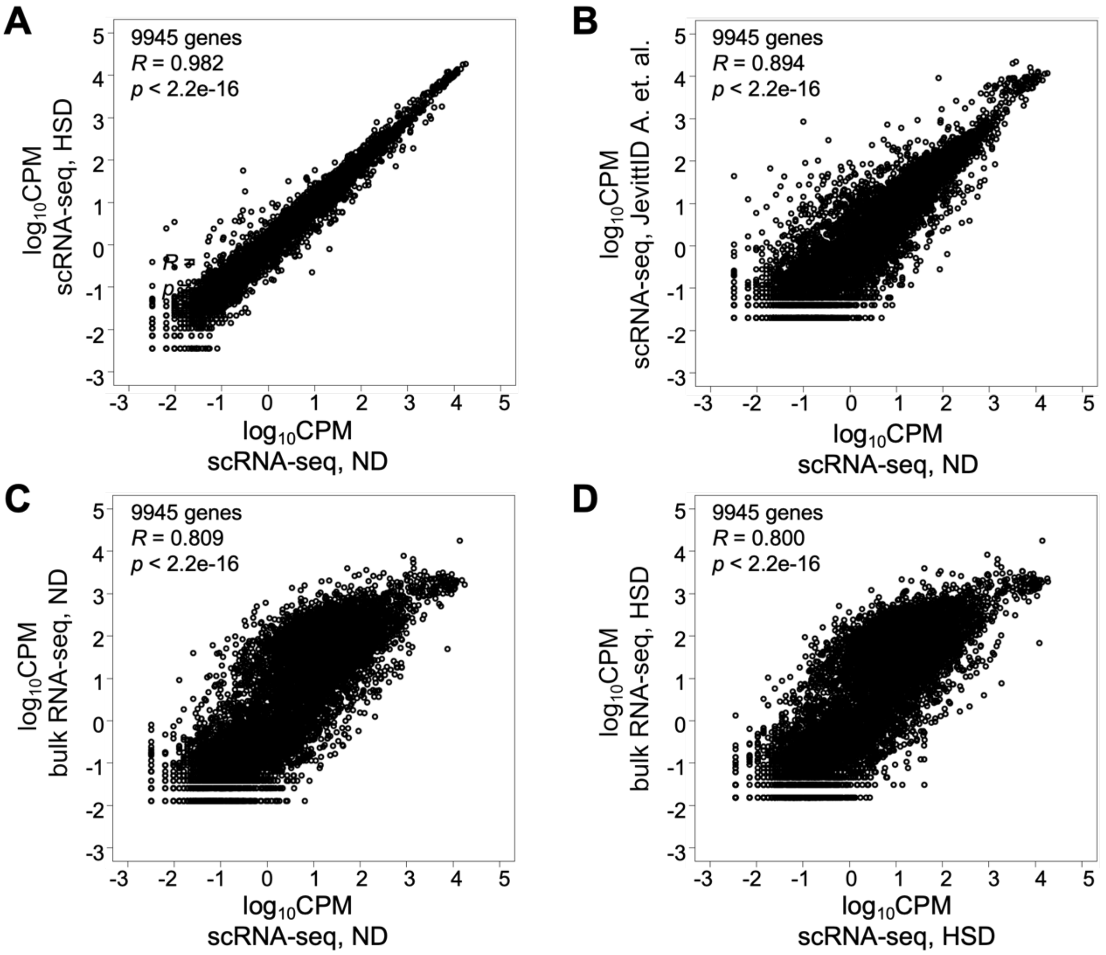


**Appendix Figure S1. Reproducibility of ovarian scRNA-seq data.**

Scatter plots showing good correlations between the two current scRNA-seq datasets of ND and HSD (A), between the ND scRNA-seq dataset and the scRNA-seq dataset by JevittID A. *et. al*. (B), between the ND scRNA-seq dataset and the corresponding bulk RNA-seq dataset (C), and between the HSD scRNA-seq dataset and the corresponding bulk RNA-seq dataset (D). Each dot represents one of the 9945 protein-coding genes with at least 3 expressing cells in every dataset. For each gene, counts per million mapped reads (CPMs) are normalized by DESeq2. Pearson's correlation was computed based on 9,945 genes that were expressed in all the samples. The correlation coefficients $R = 0.855$, 0.894, 0.809 and 0.800, respectively, and all the $p$-values are approaching 0.

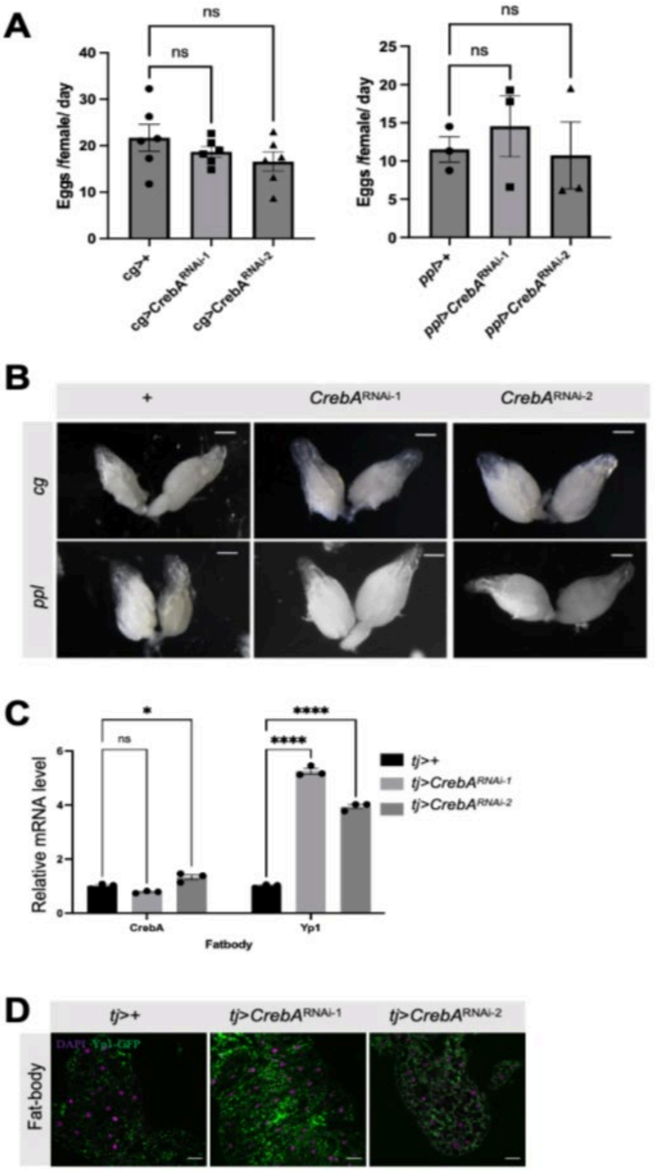


**Appendix Figure S2. Fat-body knockdown of *CrebA* has little effect on female fecundity.**

(A) Female fecundity was not significantly changed when *CrebA* was knocked down in the fat body. Data were presented as mean ± SEM. Statistical significance was determined using one-way ANOVA, ns not significant. (Left) RNAi driven by *cg-Gal4*: $N = 6$ biological replicates; *p*-values = 0.52 (denoted as ns) and 0.20 (ns). (Right) RNAi driven by *ppl*-*Gal4*: $N = 3$ biological replicates; *p*-values = 0.78 (ns) and 0.98 (ns).

(B) Representative images showing the normal ovarian morphology when *CrebA* was knocked down in the fat body. Scale bars = 200 μm.

(C) RT-qPCR shows that the *CrebA* mRNA had little changes in the fat body of *tj>CrebA*$^{RNAi\text{-}1}$ and *tj>CrebA*$^{RNAi\text{-}2}$ females, but the *Yp1* mRNA was significantly increased. For each genotype, fat body tissues were dissected from 7 females and pooled as one sample. Each data point was presented as mean ± SEM measured from 3 biological replicates. Statistical significance was determined using one-way ANOVA. For *CrebA*, $p = 0.10$ (denoted as ns) and 0.02 (denoted as *), respectively; for *Yp1*, *p*-values < 0.0001 (denoted as ****).

(D) Yp1-GFP was increased in the fat body of *tj>CrebA*$^{RNAi\text{-}1}$ and *tj>CrebA*$^{RNAi\text{-}2}$ females. Scale bars = 20 μm.

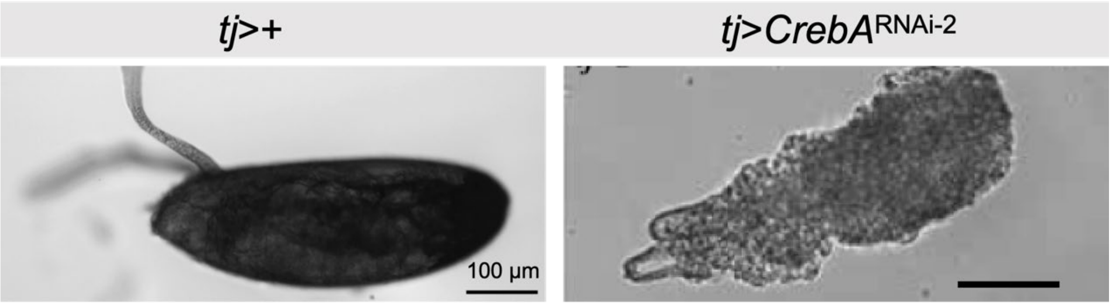


**Appendix Figure S3. The very few eggs deposited by *tj>CrebA*$^{RNAi-2}$ females show eggshell deformation.**

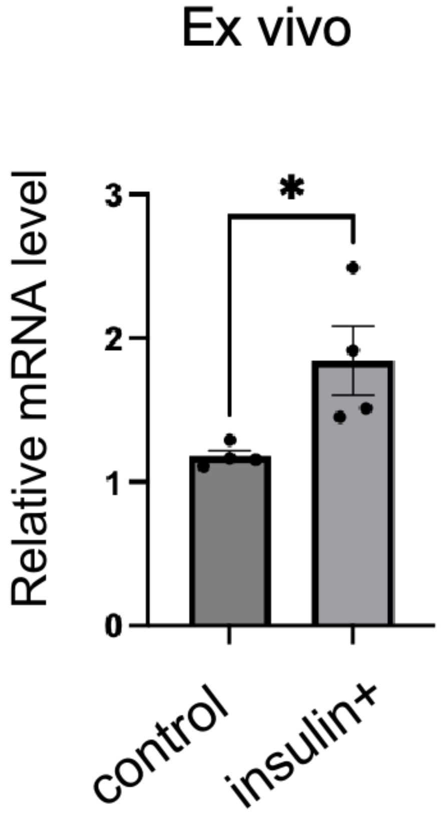


**Appendix Figure S4. Treatment of cultured *Drosophila* ovaries with human insulin increased the *CrebA* mRNA.**

For each experimental group, 7 pairs of ovaries were freshly dissected from the *w1118* females and pooled as one sample. Each data point was presented as mean ± SEM measured from four RT-qPCR biological replicates. Two-tailed Student's t-test *p*-value = 0.03 (denoted as *).
