# Supplementary figures and images for: An insulin receptor activity surge in follicle cells drives vitellogenesis by upregulating CrebA (part 1 of 2)
Source: EMBO Rep. 2026 Jan 3;27(3):748–73. doi: 10.1038/s44319-025-00672-6 (PMC12894986; doi:10.1038/s44319-025-00672-6)

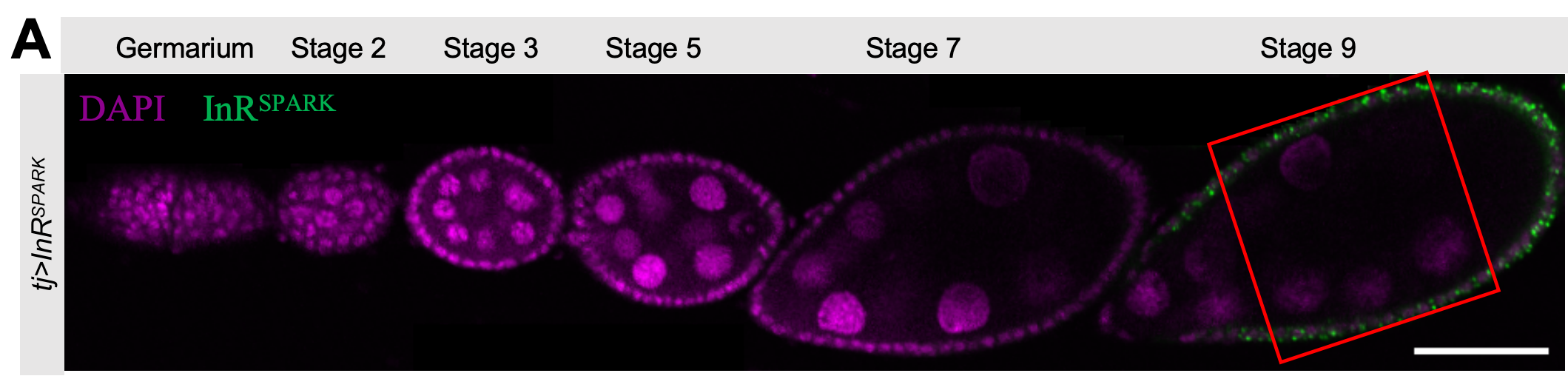

Supplement: Supplementary file 3 — Source data Fig. 1 [file 44319_2025_672_MOESM3_ESM.zip › Figure1/A/SPARK-stage1-9.tif]

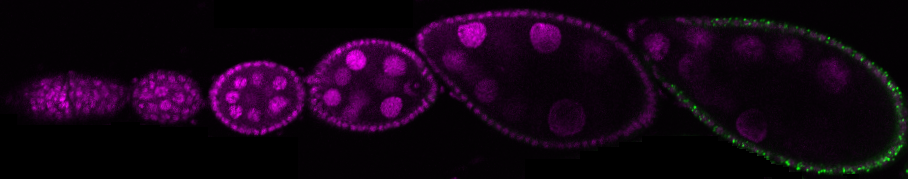

Supplement: Supplementary file 3 — Source data Fig. 1 [file 44319_2025_672_MOESM3_ESM.zip › Figure1/A/SPARKstage1-9-merge.tif]

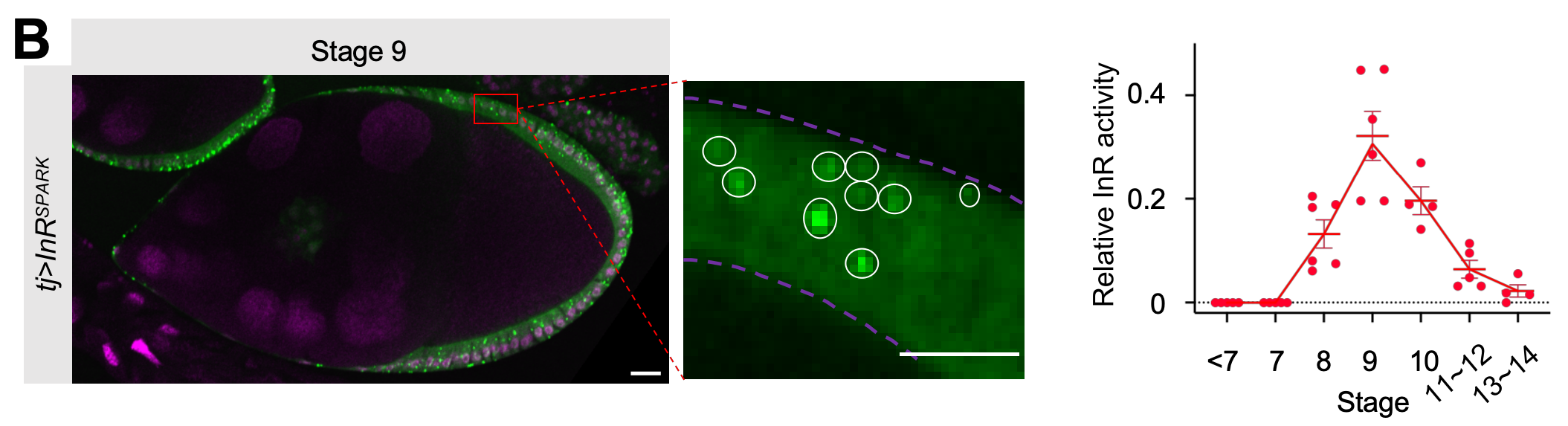

Supplement: Supplementary file 3 — Source data Fig. 1 [file 44319_2025_672_MOESM3_ESM.zip › Figure1/B/B.tif]

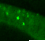

Supplement: Supplementary file 3 — Source data Fig. 1 [file 44319_2025_672_MOESM3_ESM.zip › Figure1/B/magnified.tif]

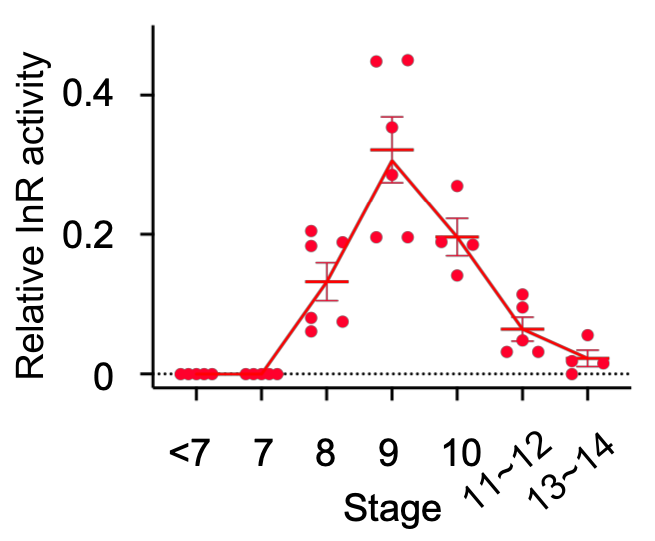

Supplement: Supplementary file 3 — Source data Fig. 1 [file 44319_2025_672_MOESM3_ESM.zip › Figure1/B/Right.tif]

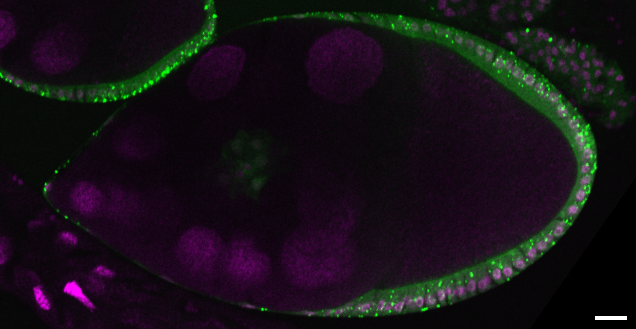

Supplement: Supplementary file 3 — Source data Fig. 1 [file 44319_2025_672_MOESM3_ESM.zip › Figure1/B/stgae9.tif]

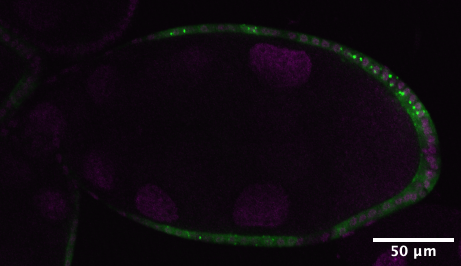

Supplement: Supplementary file 3 — Source data Fig. 1 [file 44319_2025_672_MOESM3_ESM.zip › Figure1/C/stage9-InRDNspark.tif]

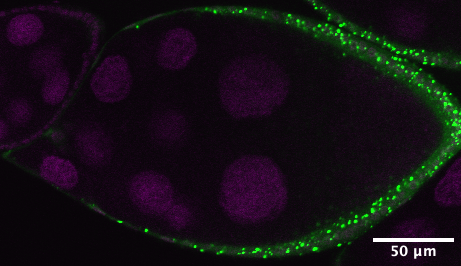

Supplement: Supplementary file 3 — Source data Fig. 1 [file 44319_2025_672_MOESM3_ESM.zip › Figure1/C/stage9-InRspark.tif]

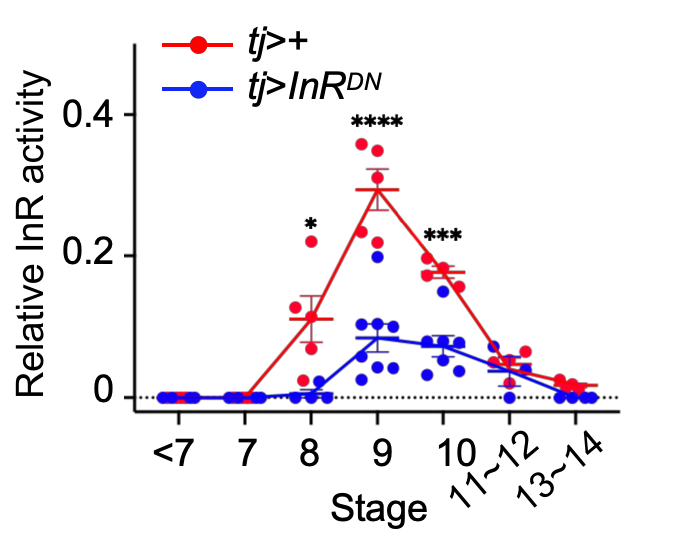

Supplement: Supplementary file 3 — Source data Fig. 1 [file 44319_2025_672_MOESM3_ESM.zip › Figure1/D/D.tif]

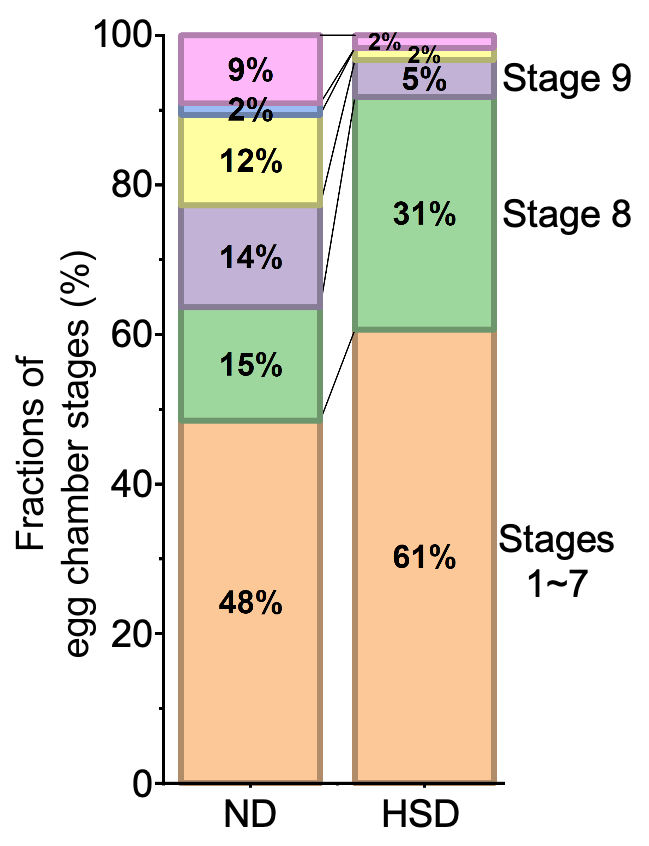

Supplement: Supplementary file 3 — Source data Fig. 1 [file 44319_2025_672_MOESM3_ESM.zip › Figure1/E/Distributions of egg chambers.tif]

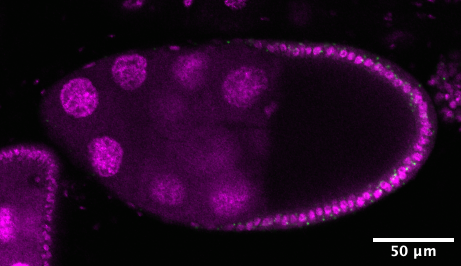

Supplement: Supplementary file 3 — Source data Fig. 1 [file 44319_2025_672_MOESM3_ESM.zip › Figure1/F/HSD-spark-stage9.tif]

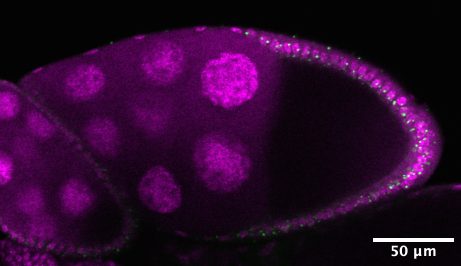

Supplement: Supplementary file 3 — Source data Fig. 1 [file 44319_2025_672_MOESM3_ESM.zip › Figure1/F/ND-spark- stage9.tif]

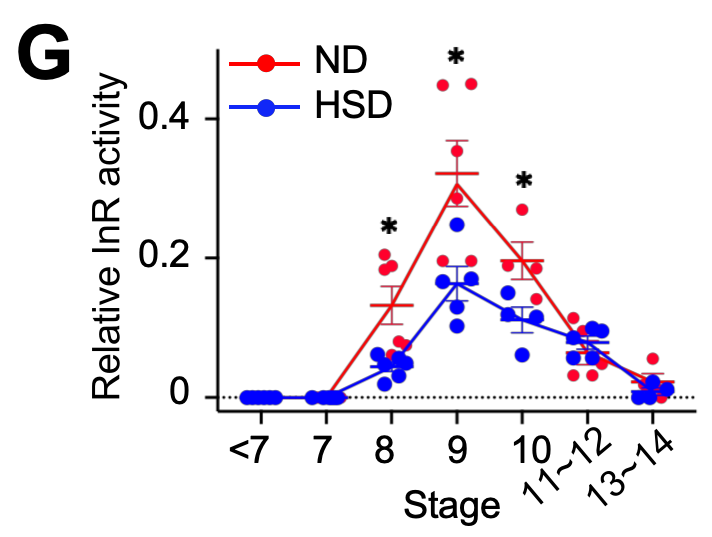

Supplement: Supplementary file 3 — Source data Fig. 1 [file 44319_2025_672_MOESM3_ESM.zip › Figure1/G/G.tif]

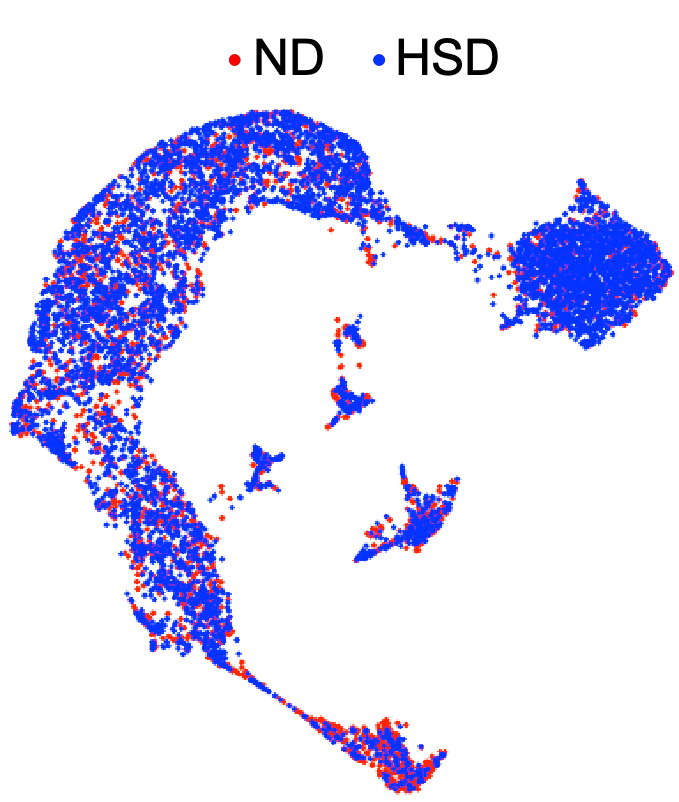

Supplement: Supplementary file 4 — Source data Fig. 2 [file 44319_2025_672_MOESM4_ESM.zip › Figure2/A/A.tif]

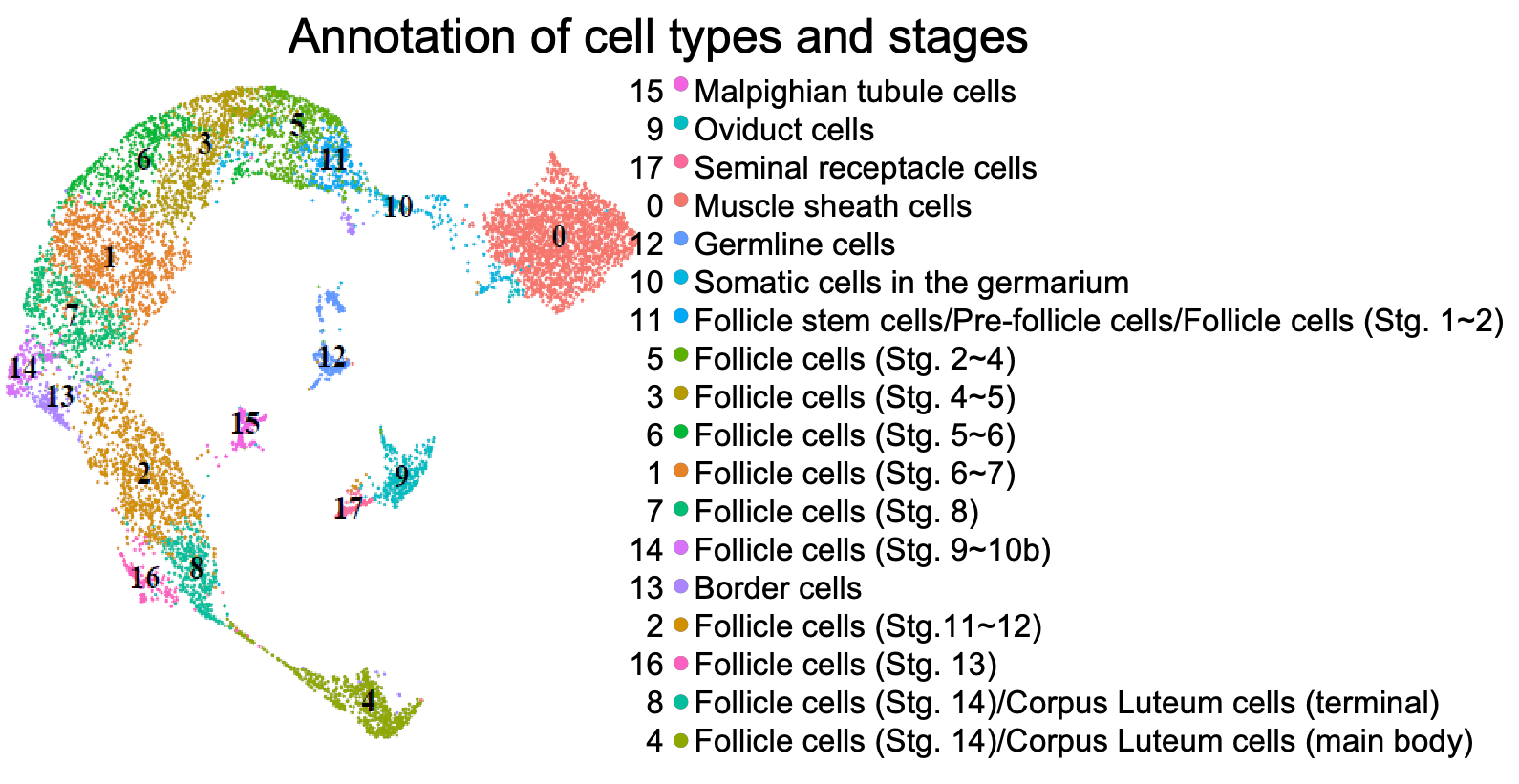

Supplement: Supplementary file 4 — Source data Fig. 2 [file 44319_2025_672_MOESM4_ESM.zip › Figure2/B/B.tif]

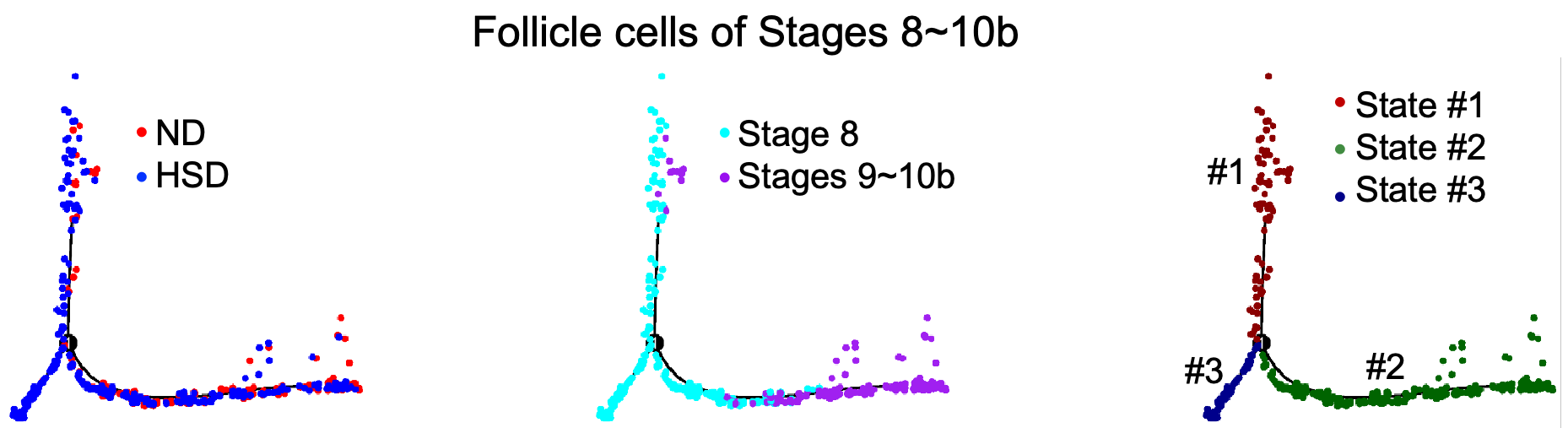

Supplement: Supplementary file 4 — Source data Fig. 2 [file 44319_2025_672_MOESM4_ESM.zip › Figure2/C/arichive.tif]

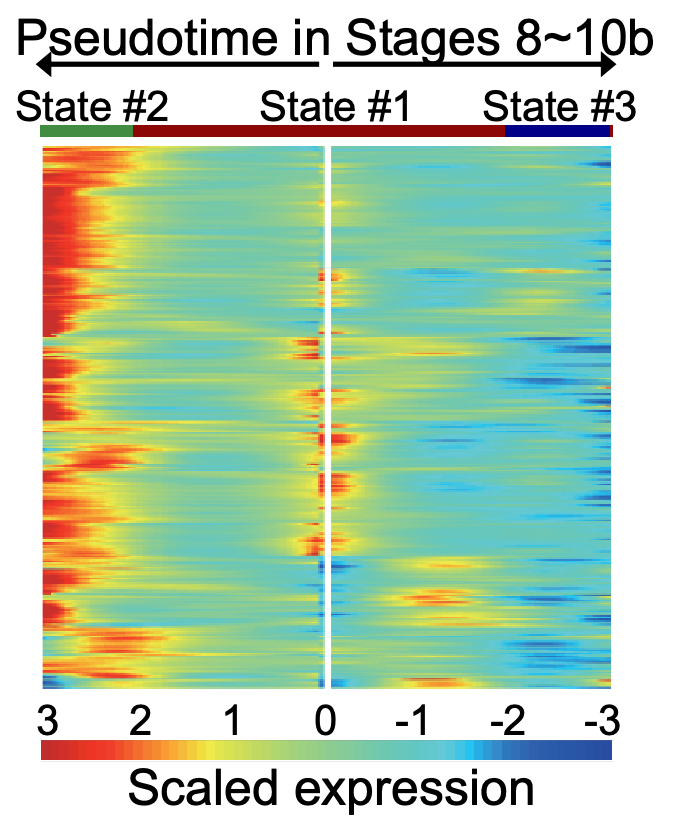

Supplement: Supplementary file 4 — Source data Fig. 2 [file 44319_2025_672_MOESM4_ESM.zip › Figure2/D/map.tif]

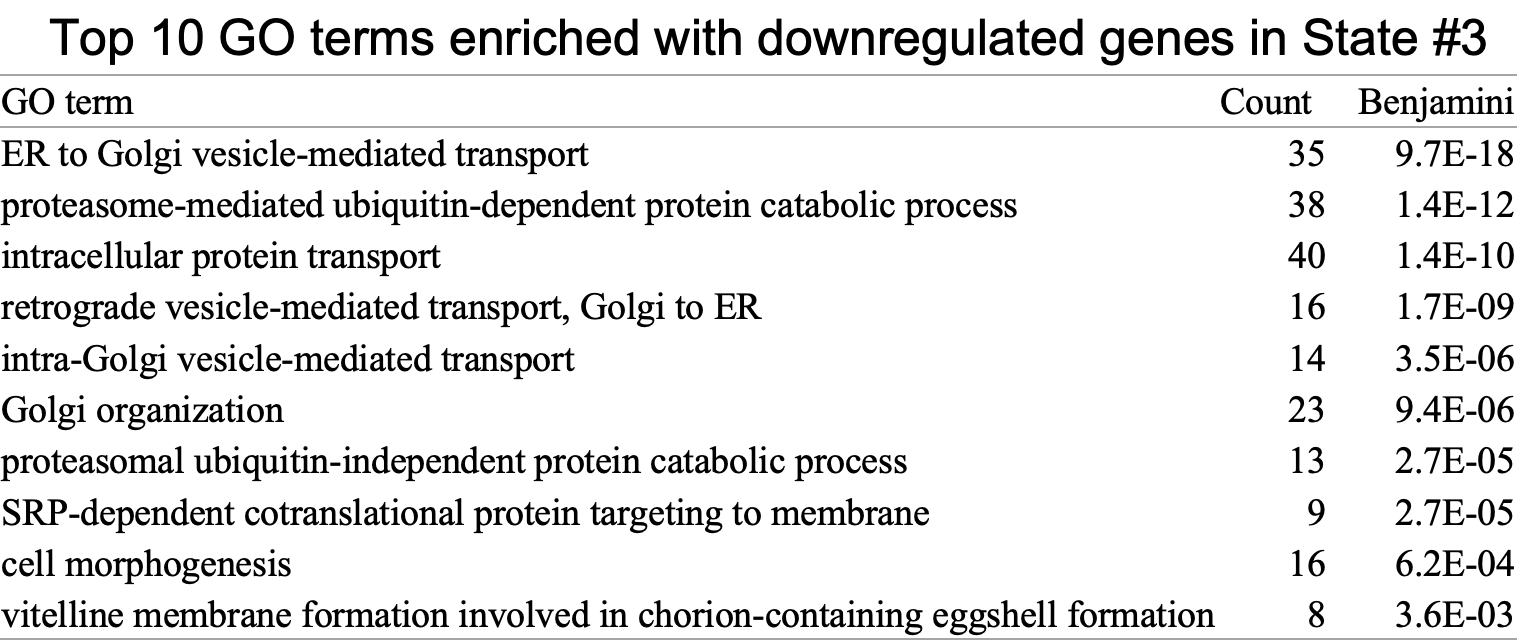

Supplement: Supplementary file 4 — Source data Fig. 2 [file 44319_2025_672_MOESM4_ESM.zip › Figure2/E/GO term.tif]

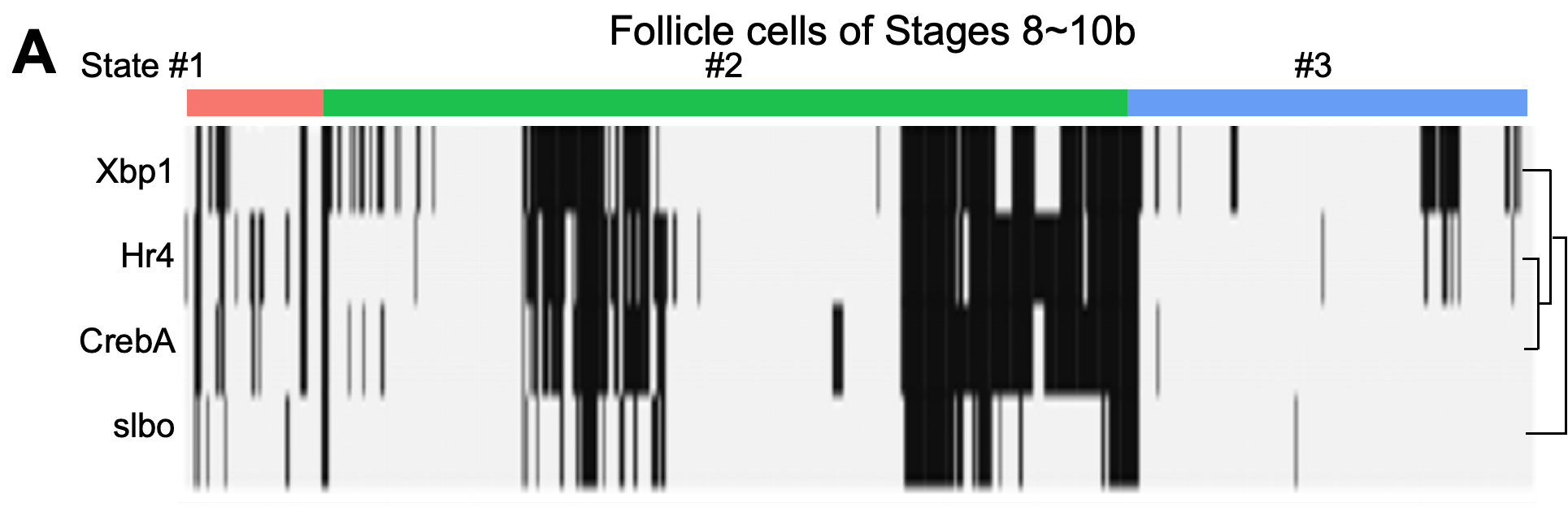

Supplement: Supplementary file 5 — Source data Fig. 3 [file 44319_2025_672_MOESM5_ESM.zip › Figure3/A/Atif.tif]

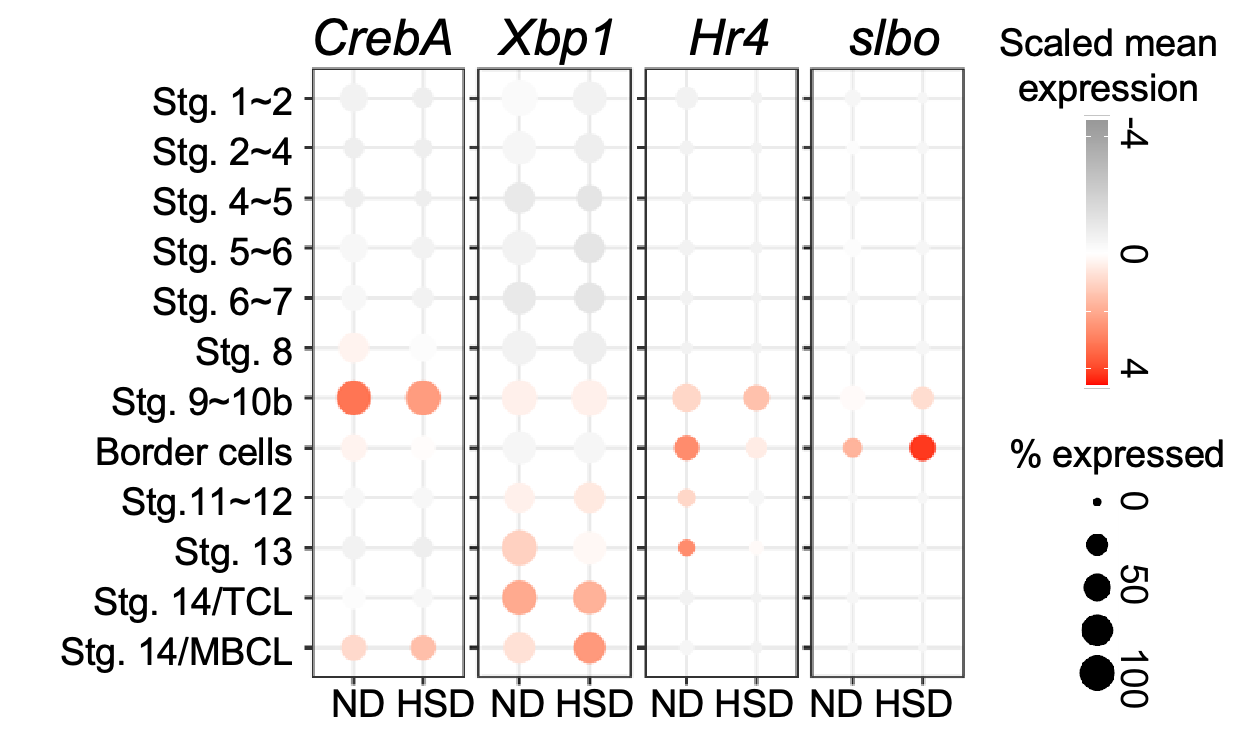

Supplement: Supplementary file 5 — Source data Fig. 3 [file 44319_2025_672_MOESM5_ESM.zip › Figure3/B/B.tif]

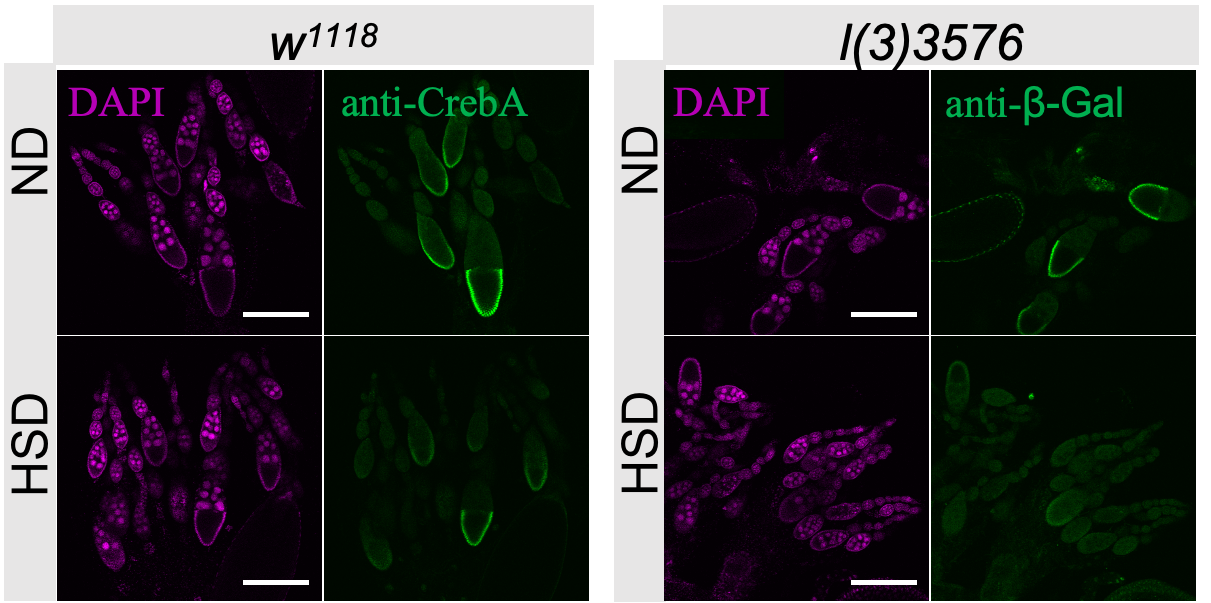

Supplement: Supplementary file 5 — Source data Fig. 3 [file 44319_2025_672_MOESM5_ESM.zip › Figure3/C/C.tif]

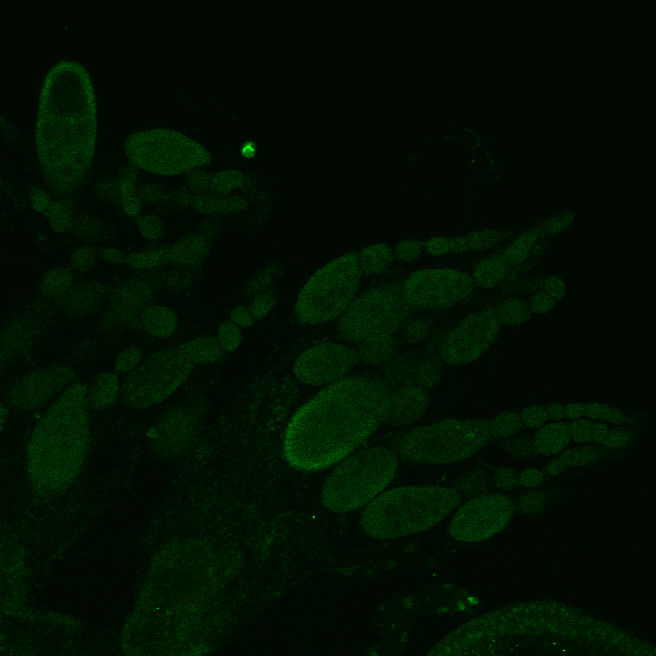

Supplement: Supplementary file 5 — Source data Fig. 3 [file 44319_2025_672_MOESM5_ESM.zip › Figure3/C/HSD-l(3)3576-anti-Gal.tif]

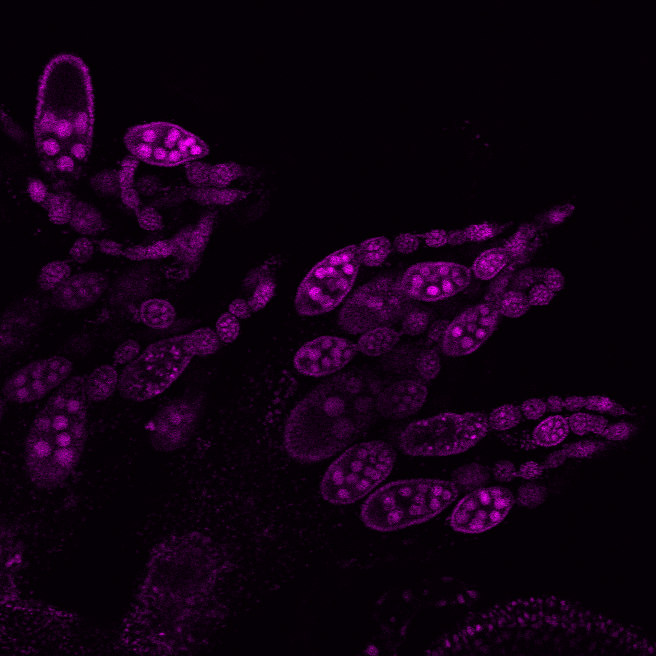

Supplement: Supplementary file 5 — Source data Fig. 3 [file 44319_2025_672_MOESM5_ESM.zip › Figure3/C/HSD-l(3)3576-DAPI.tif]

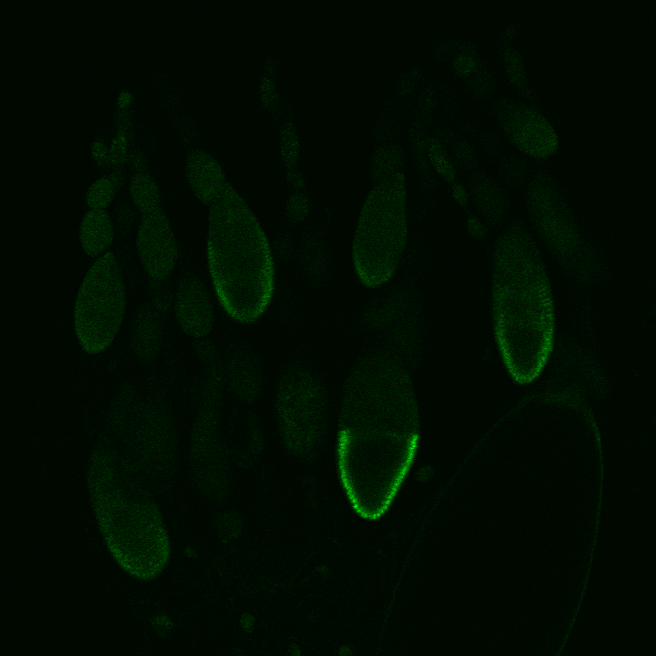

Supplement: Supplementary file 5 — Source data Fig. 3 [file 44319_2025_672_MOESM5_ESM.zip › Figure3/C/HSD-w1118-anti-CrebA.tif]

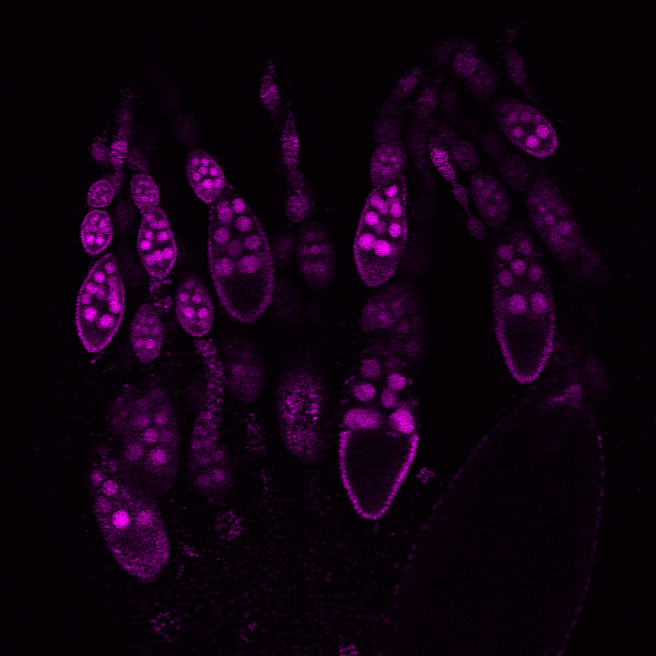

Supplement: Supplementary file 5 — Source data Fig. 3 [file 44319_2025_672_MOESM5_ESM.zip › Figure3/C/HSD-w1118-DAPI.tif]

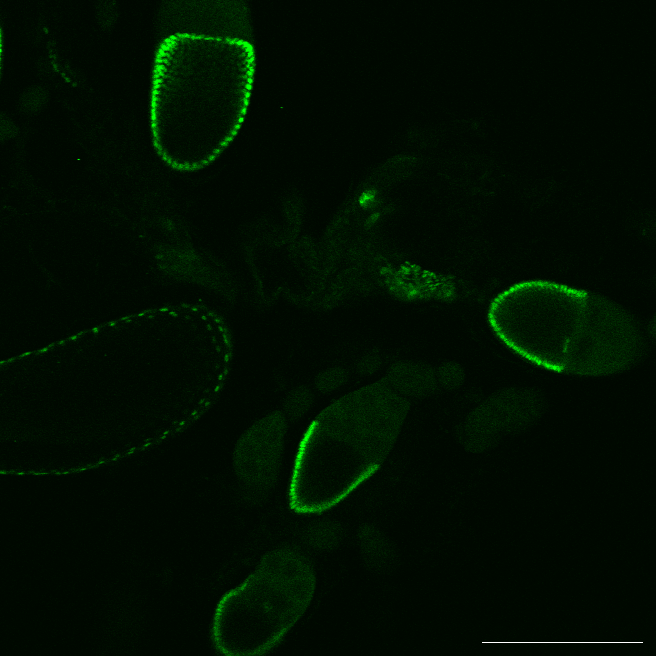

Supplement: Supplementary file 5 — Source data Fig. 3 [file 44319_2025_672_MOESM5_ESM.zip › Figure3/C/ND-l(3)3576-anti-Gal.tif]

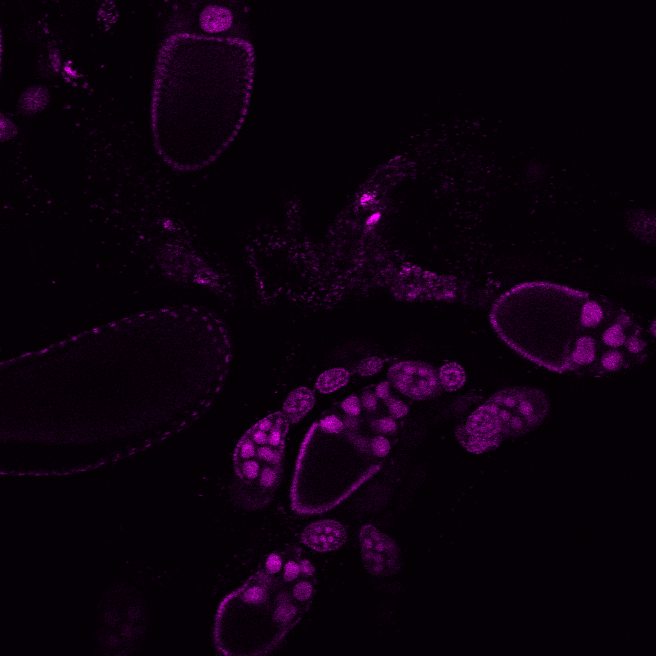

Supplement: Supplementary file 5 — Source data Fig. 3 [file 44319_2025_672_MOESM5_ESM.zip › Figure3/C/ND-l(3)3576-DAPI.tif]

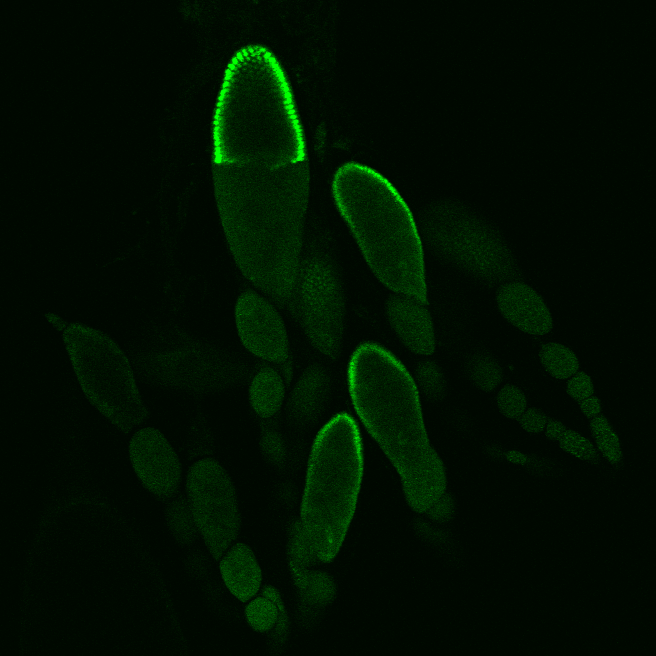

Supplement: Supplementary file 5 — Source data Fig. 3 [file 44319_2025_672_MOESM5_ESM.zip › Figure3/C/ND-w1118-anti-CrebA.tif]

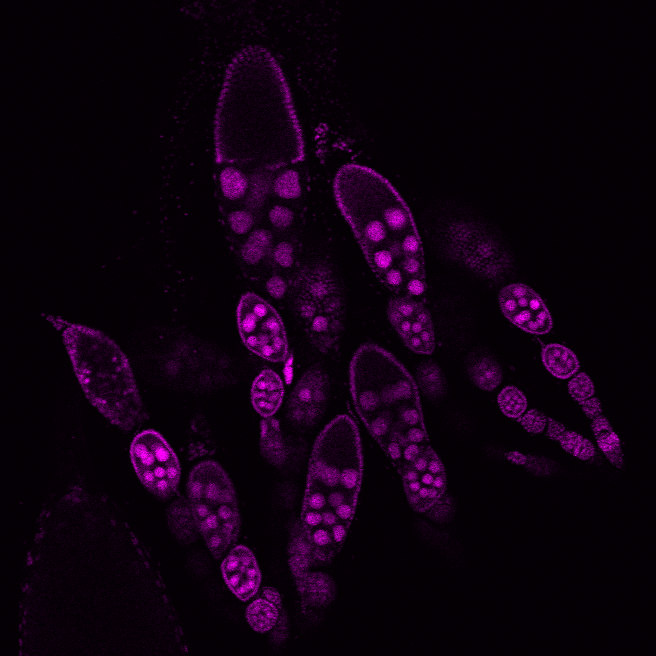

Supplement: Supplementary file 5 — Source data Fig. 3 [file 44319_2025_672_MOESM5_ESM.zip › Figure3/C/ND-w1118-DAPI.tif]

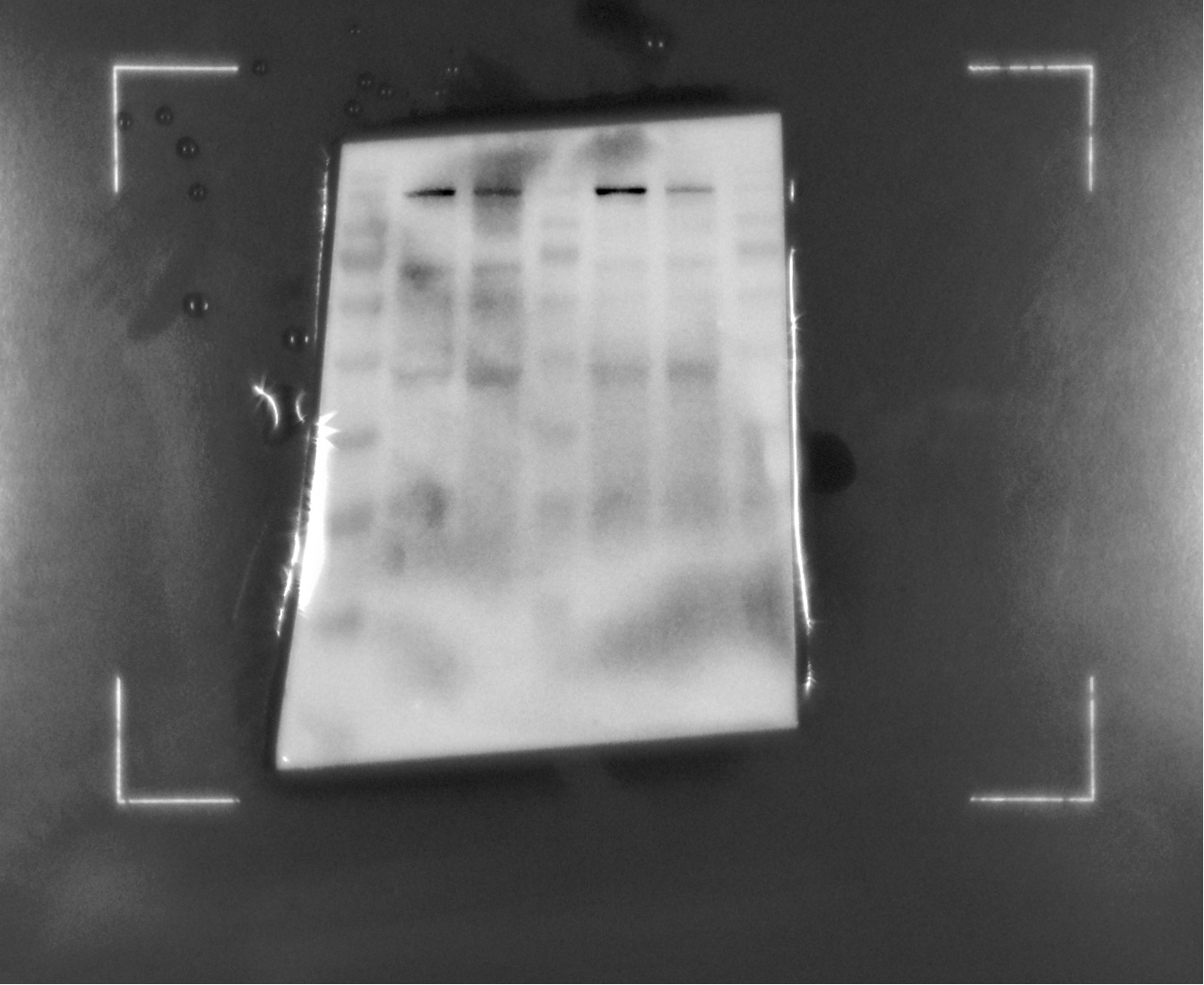

Supplement: Supplementary file 5 — Source data Fig. 3 [file 44319_2025_672_MOESM5_ESM.zip › Figure3/D/anti-Gal.jpg]

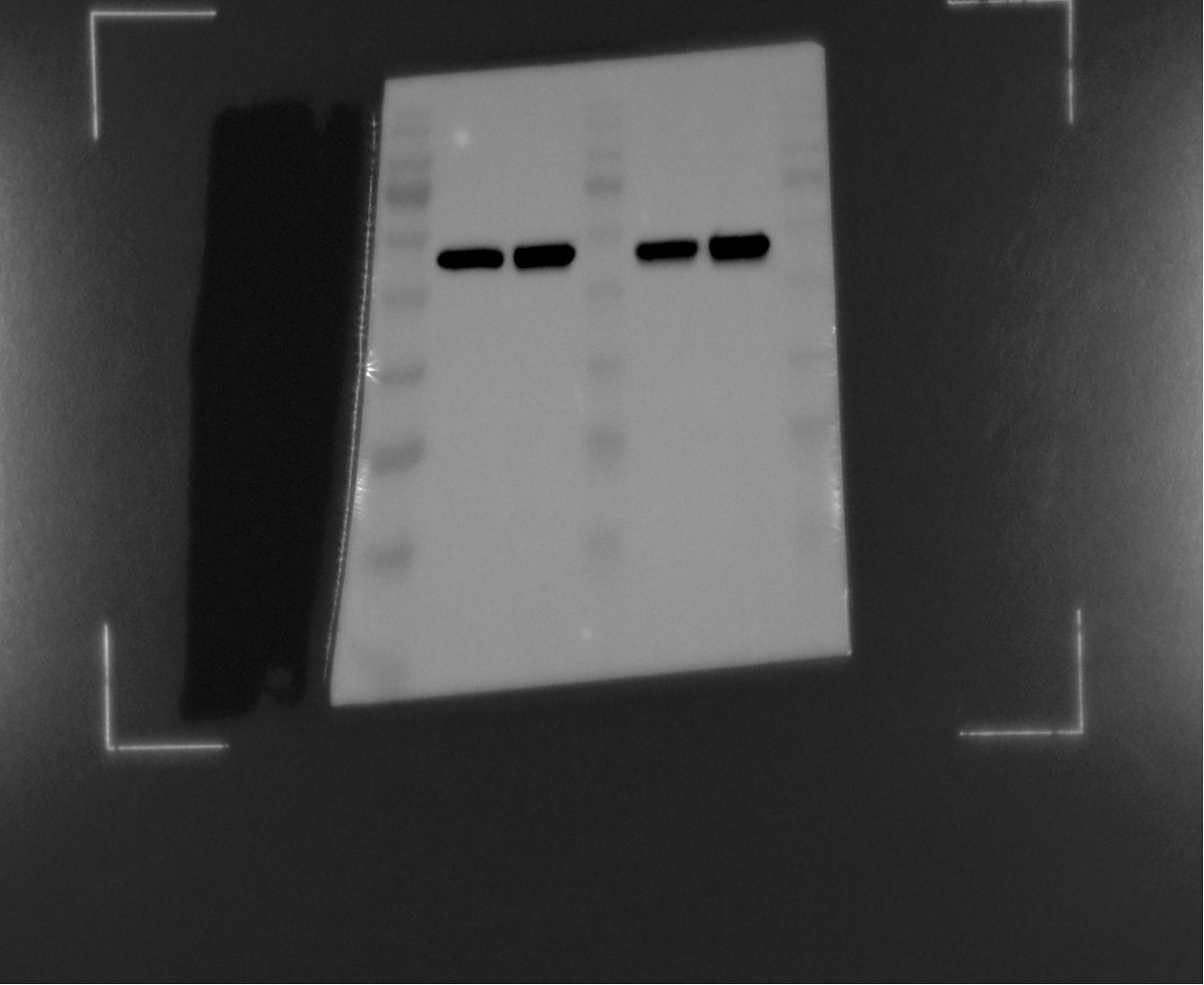

Supplement: Supplementary file 5 — Source data Fig. 3 [file 44319_2025_672_MOESM5_ESM.zip › Figure3/D/anti-Tubulin.jpg]

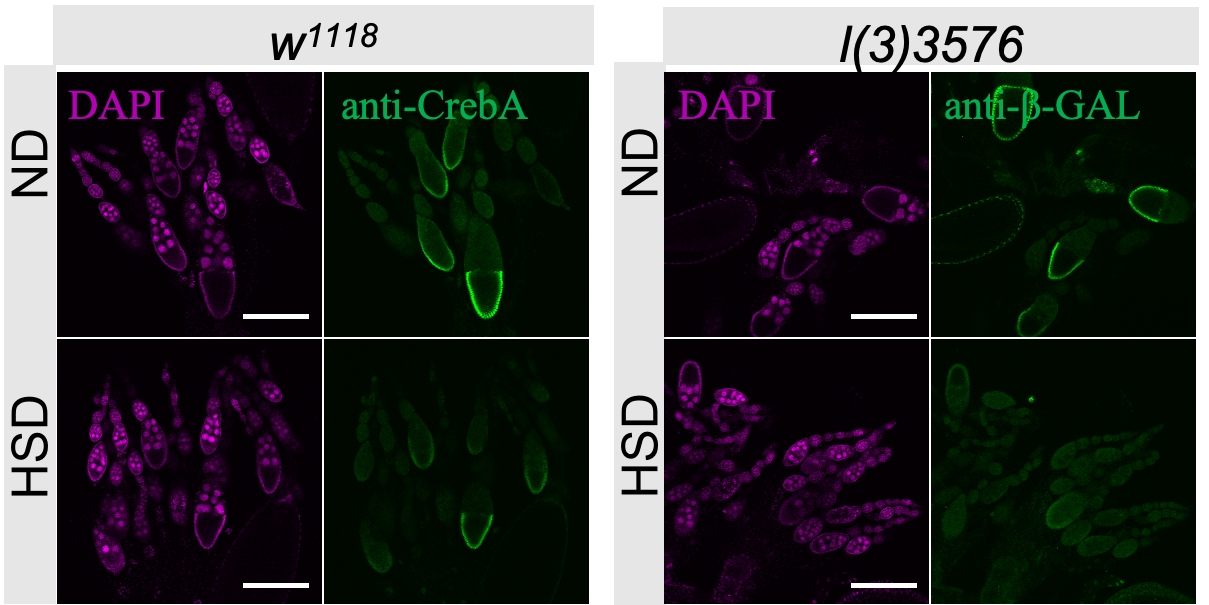

Supplement: Supplementary file 5 — Source data Fig. 3 [file 44319_2025_672_MOESM5_ESM.zip › Figure3/D/D.tif]

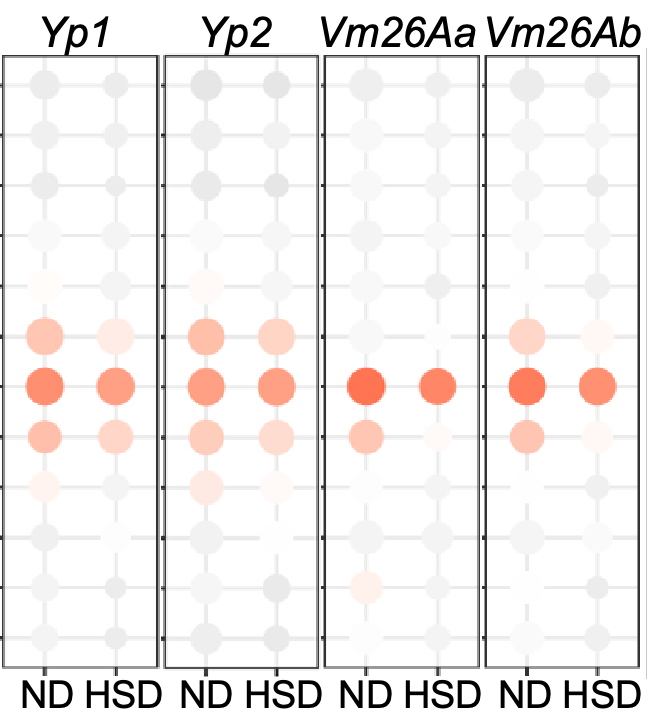

Supplement: Supplementary file 5 — Source data Fig. 3 [file 44319_2025_672_MOESM5_ESM.zip › Figure3/E/E.tif]

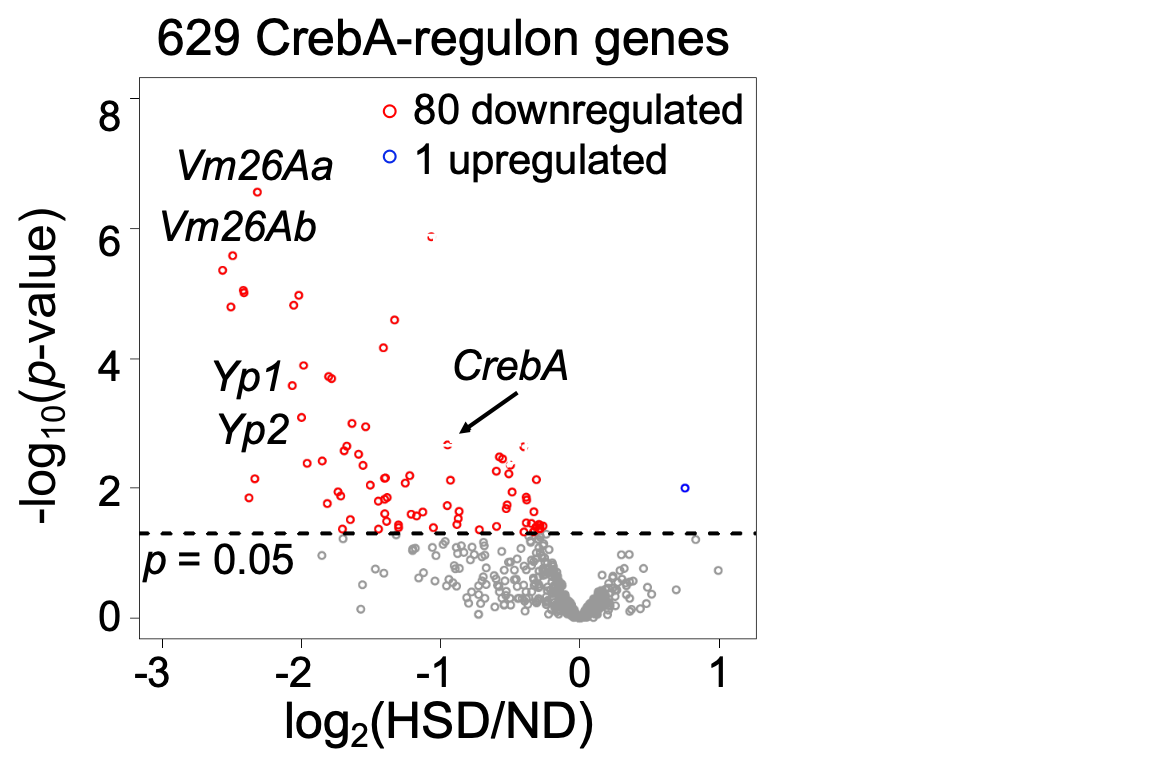

Supplement: Supplementary file 5 — Source data Fig. 3 [file 44319_2025_672_MOESM5_ESM.zip › Figure3/F/F.tif]

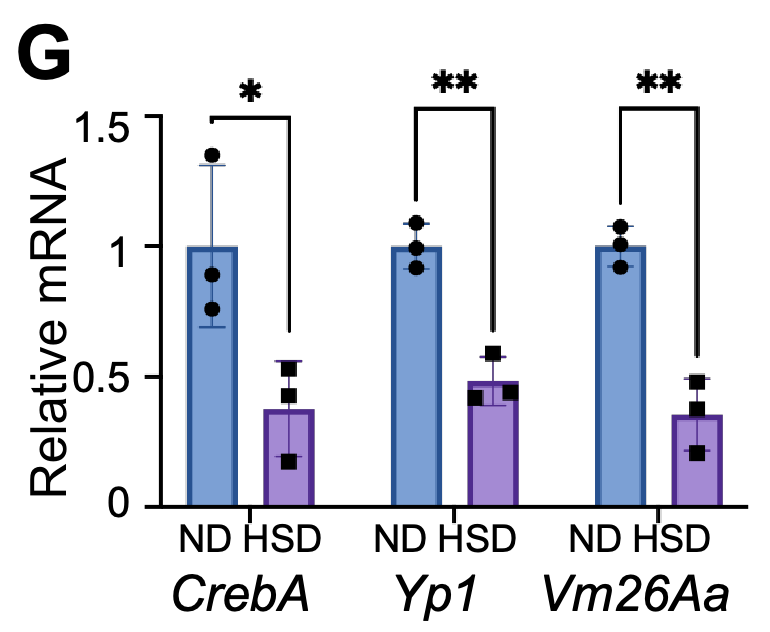

Supplement: Supplementary file 5 — Source data Fig. 3 [file 44319_2025_672_MOESM5_ESM.zip › Figure3/G/G.tif]

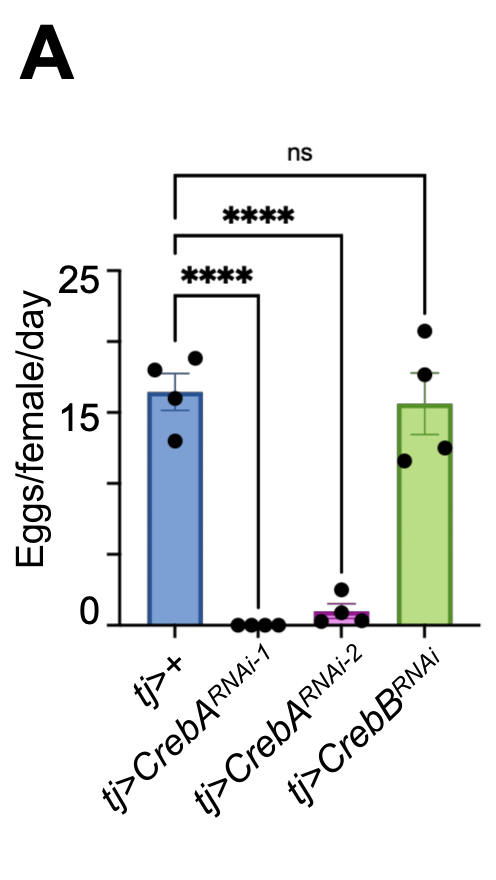

Supplement: Supplementary file 6 — Source data Fig. 4 [file 44319_2025_672_MOESM6_ESM.zip › Figure4/A/A.tif]

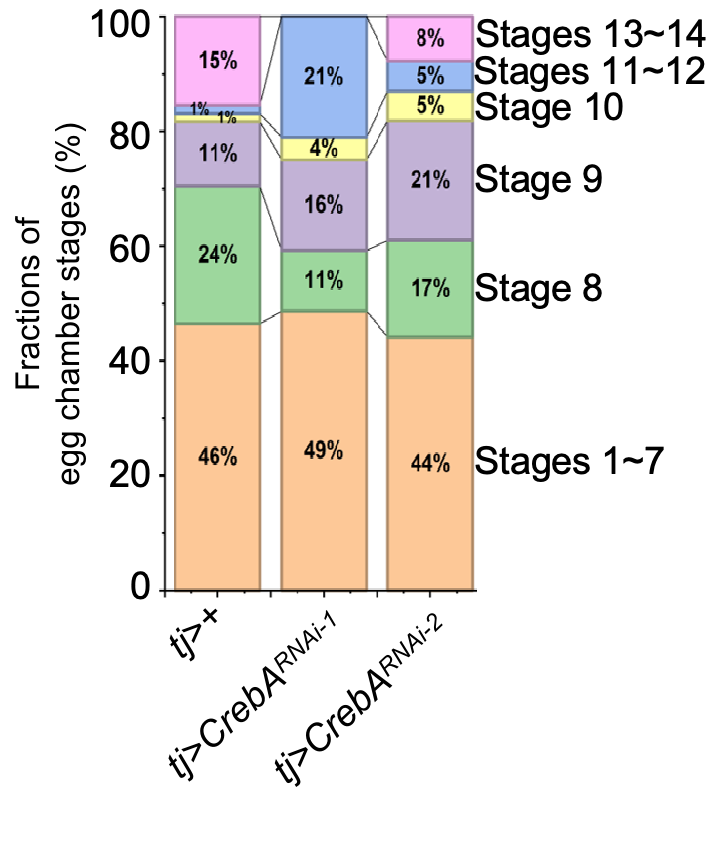

Supplement: Supplementary file 6 — Source data Fig. 4 [file 44319_2025_672_MOESM6_ESM.zip › Figure4/B/B.tif]

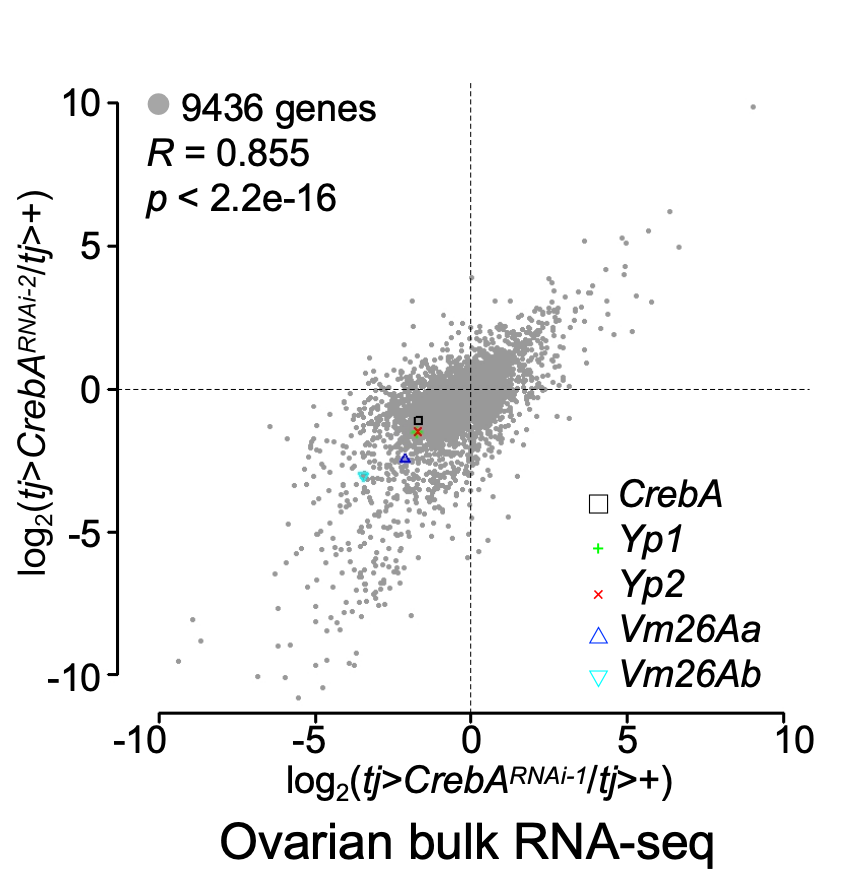

Supplement: Supplementary file 6 — Source data Fig. 4 [file 44319_2025_672_MOESM6_ESM.zip › Figure4/C/C.tif]

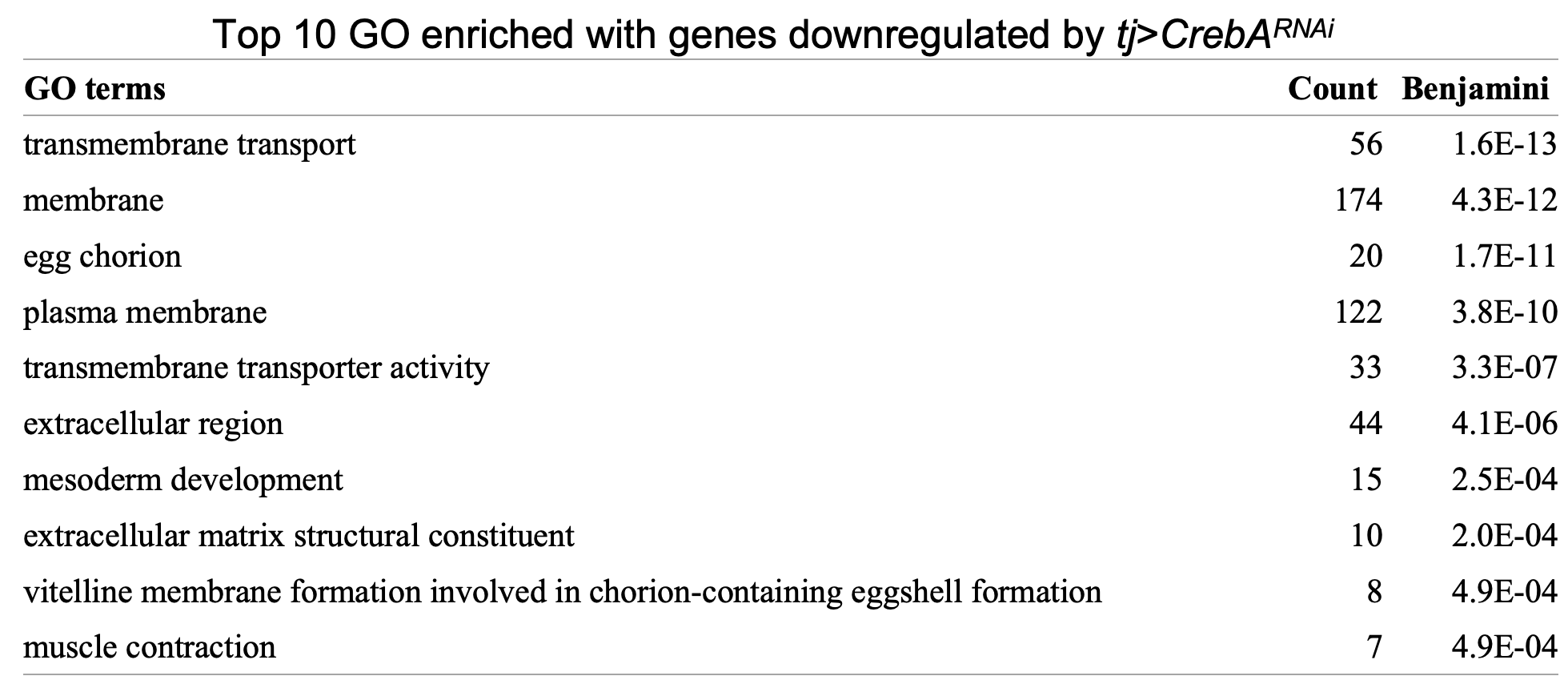

Supplement: Supplementary file 6 — Source data Fig. 4 [file 44319_2025_672_MOESM6_ESM.zip › Figure4/D/D.tif]

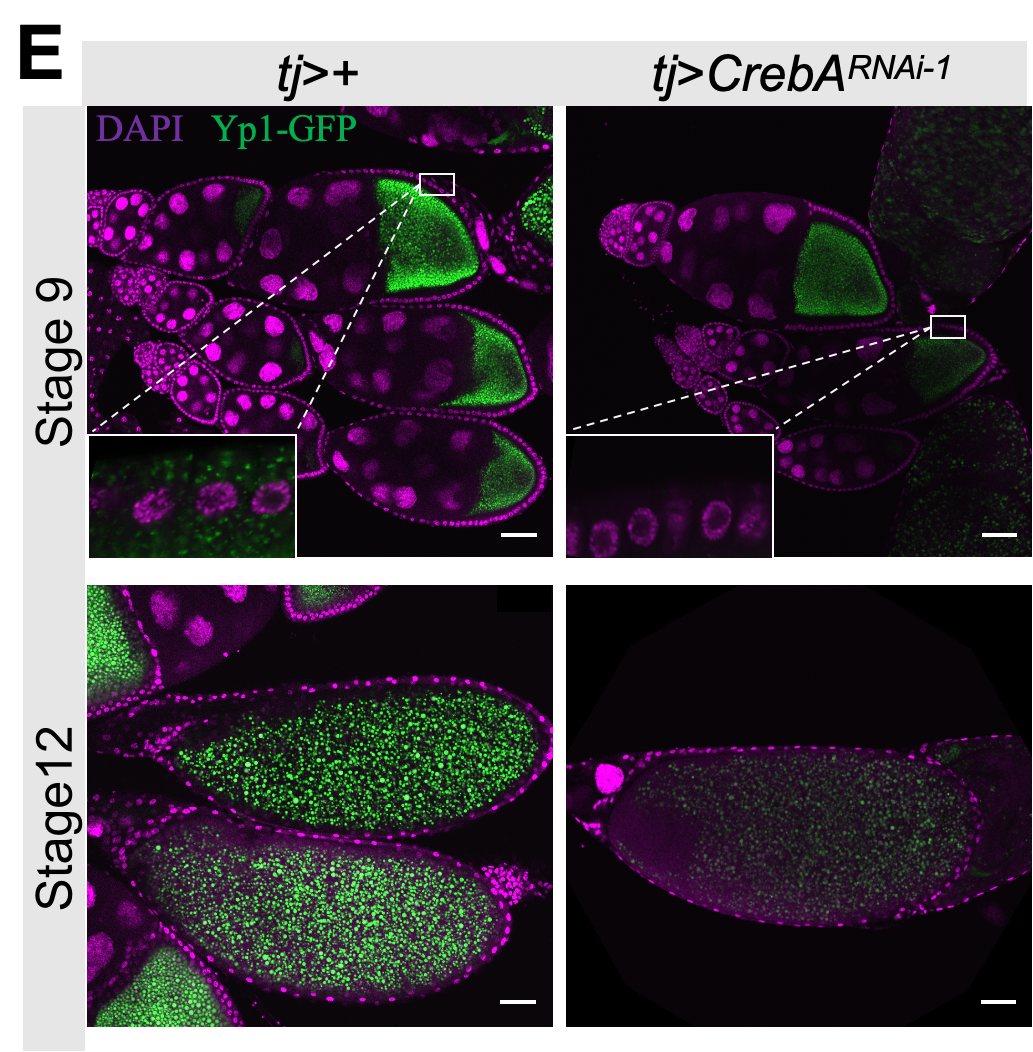

Supplement: Supplementary file 6 — Source data Fig. 4 [file 44319_2025_672_MOESM6_ESM.zip › Figure4/E/E.tif]

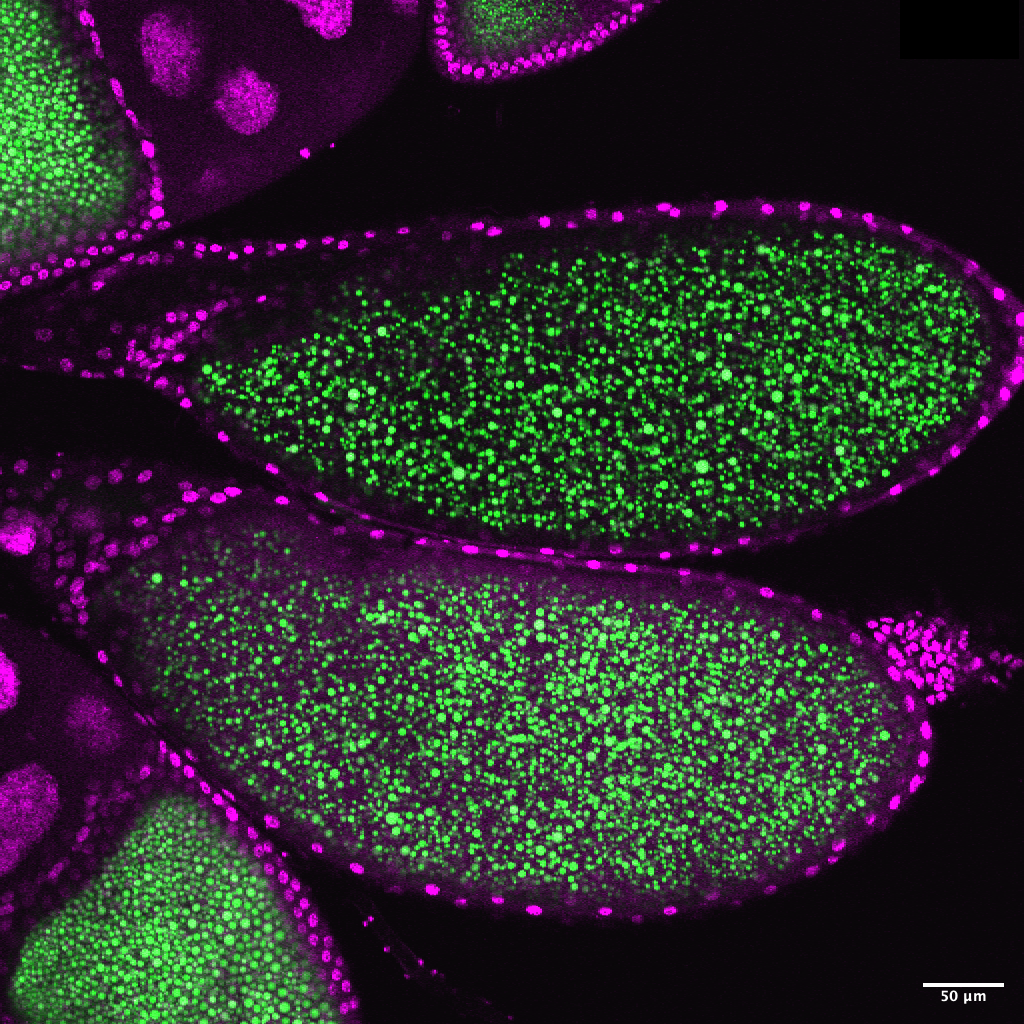

Supplement: Supplementary file 6 — Source data Fig. 4 [file 44319_2025_672_MOESM6_ESM.zip › Figure4/E/tj_+ stage12.tif]

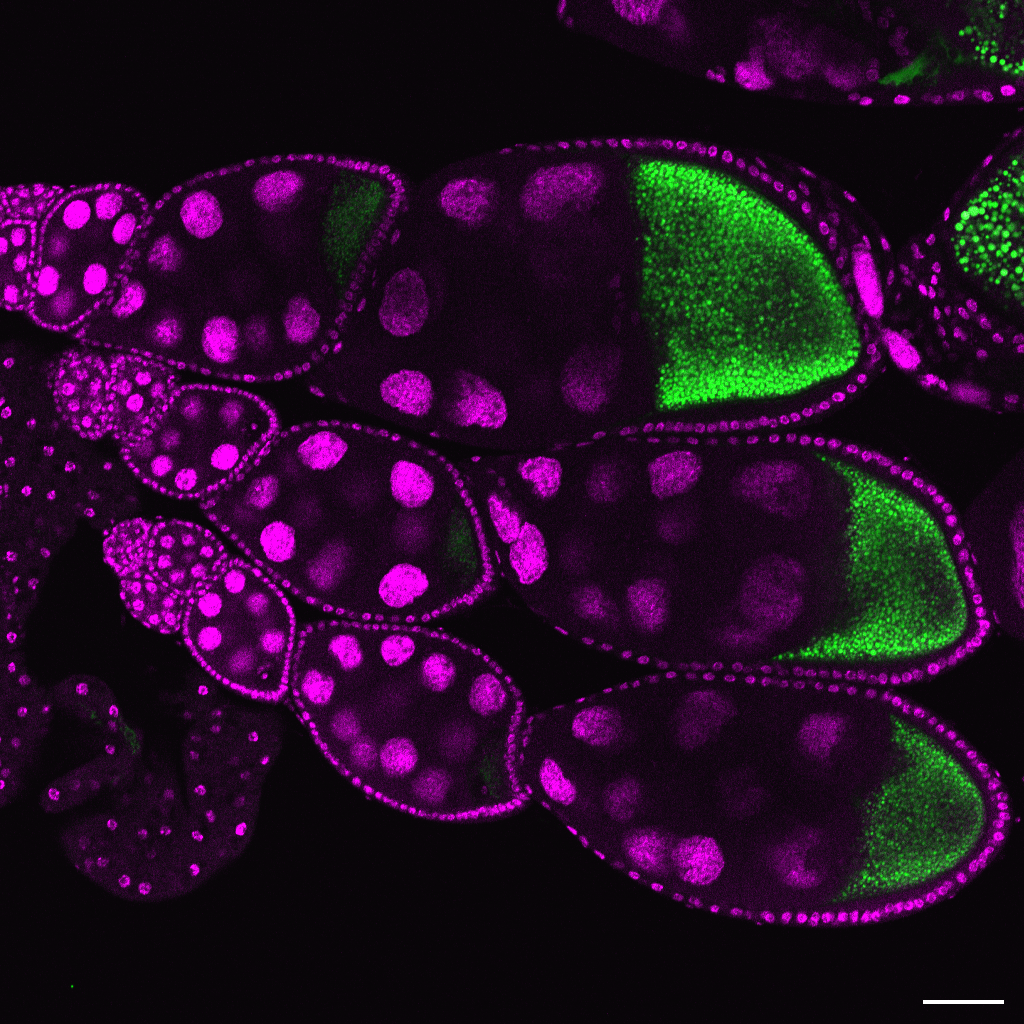

Supplement: Supplementary file 6 — Source data Fig. 4 [file 44319_2025_672_MOESM6_ESM.zip › Figure4/E/tj_+ stage9.tif]

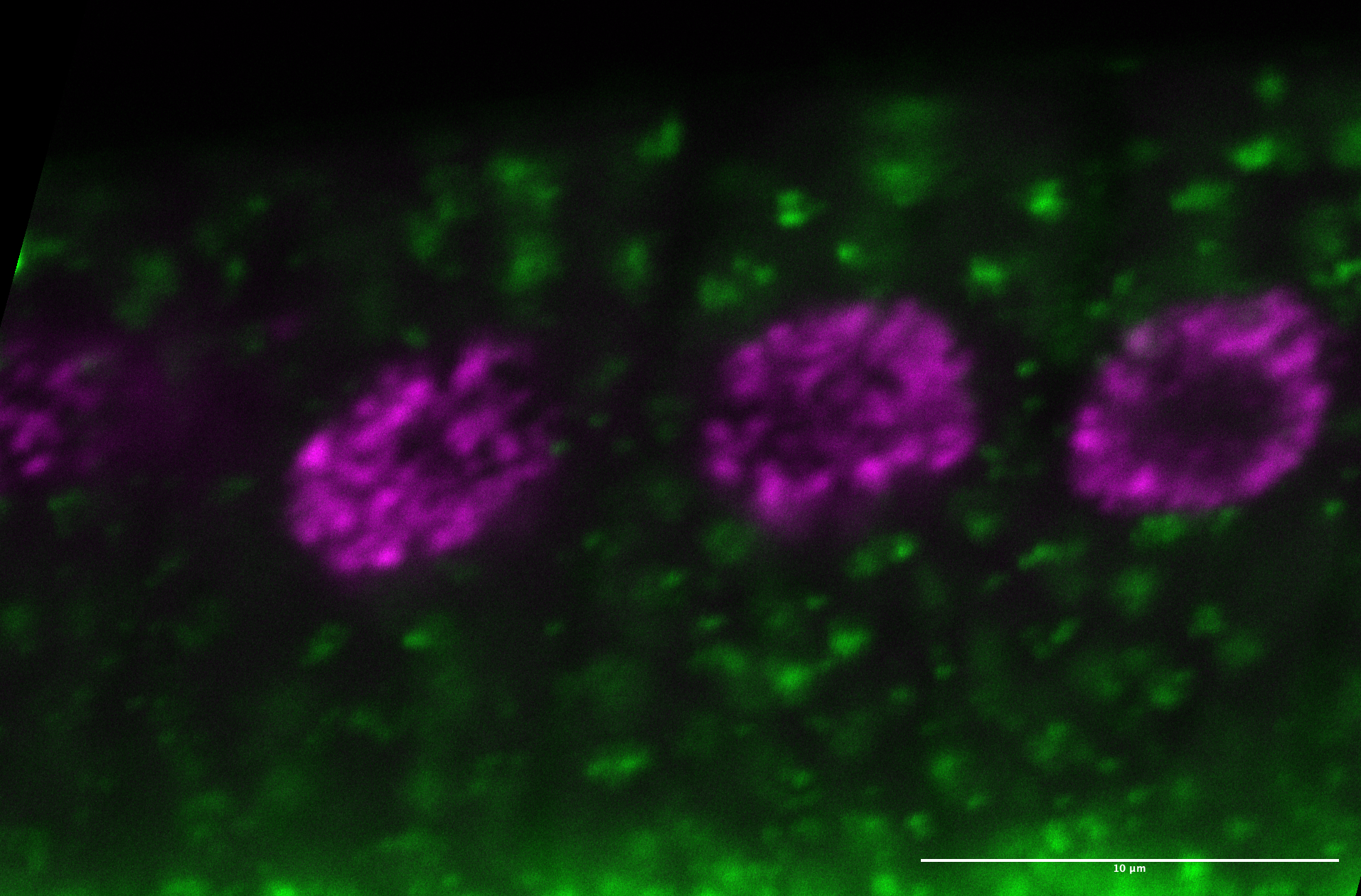

Supplement: Supplementary file 6 — Source data Fig. 4 [file 44319_2025_672_MOESM6_ESM.zip › Figure4/E/tj_+ stage9-magnified.tif]

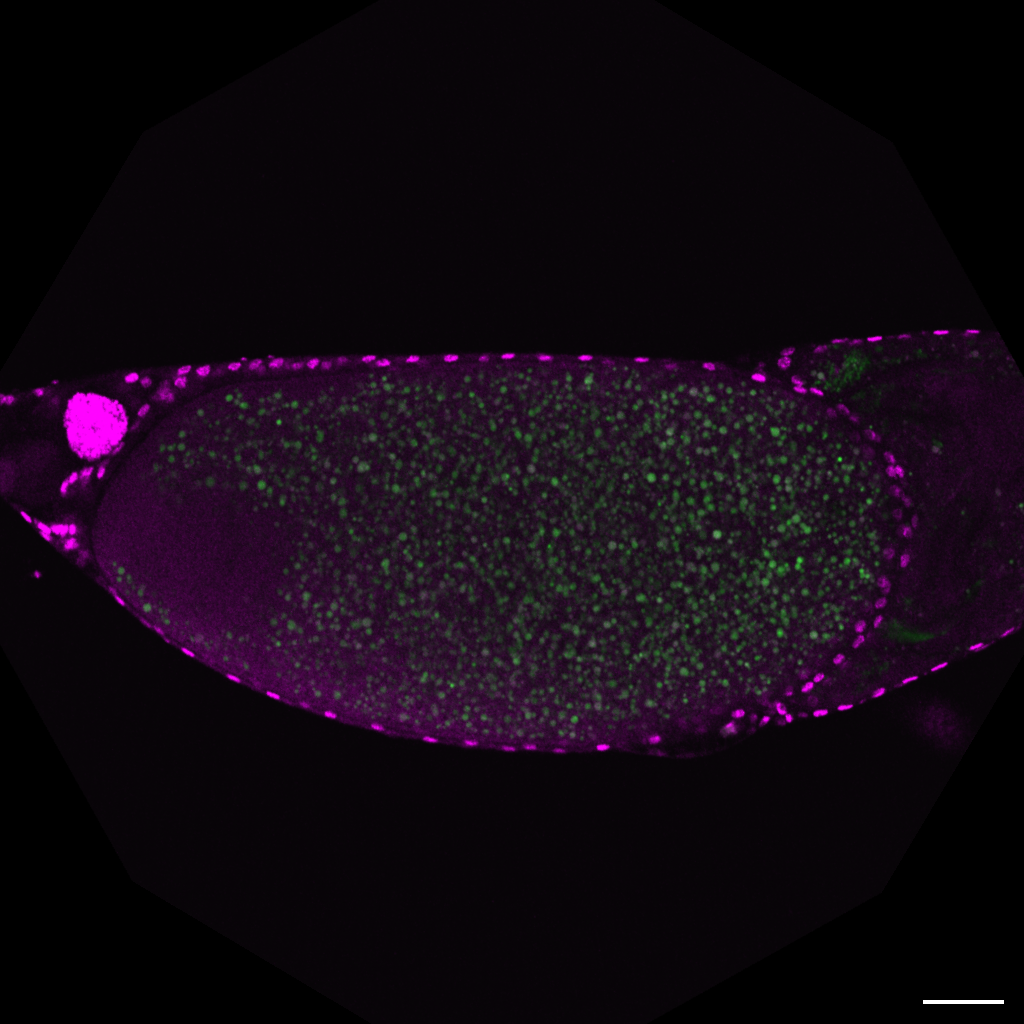

Supplement: Supplementary file 6 — Source data Fig. 4 [file 44319_2025_672_MOESM6_ESM.zip › Figure4/E/tj_CrebA RNAi1-stage12.tif]

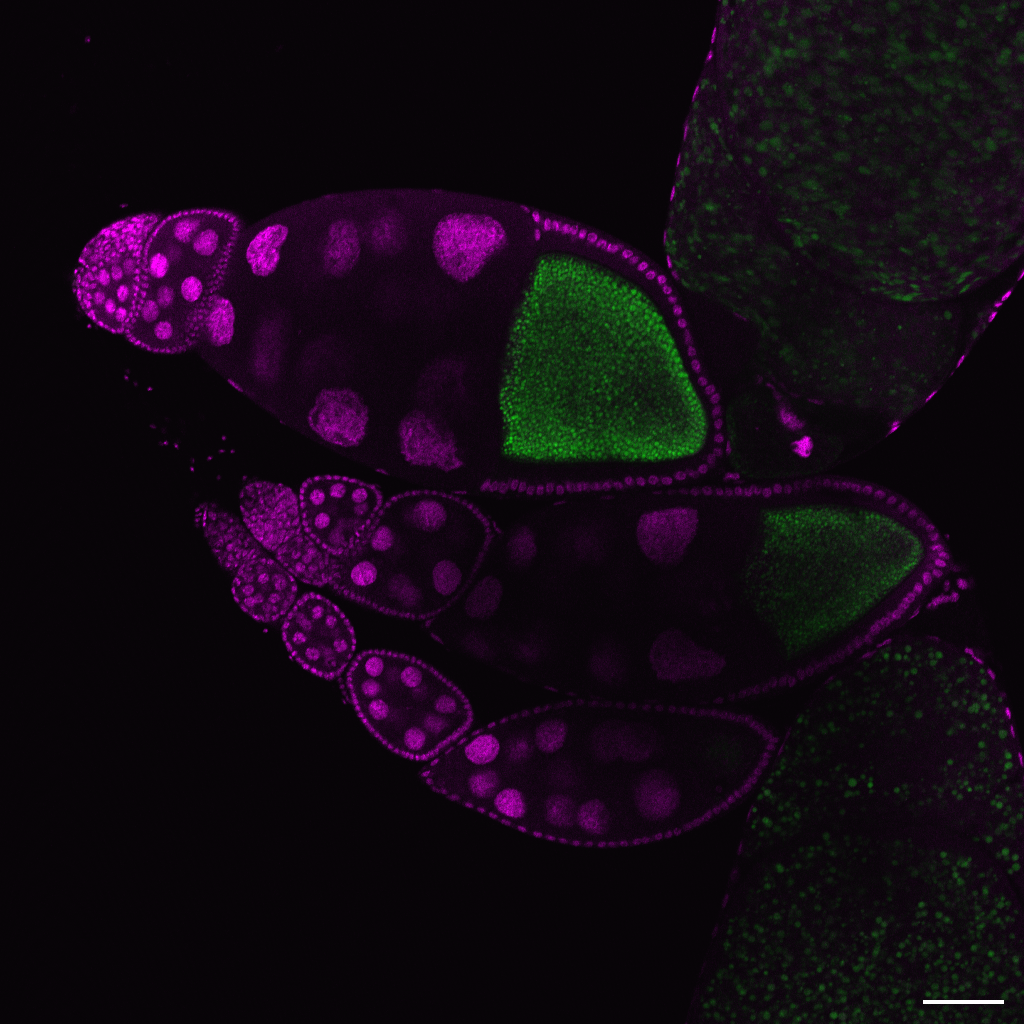

Supplement: Supplementary file 6 — Source data Fig. 4 [file 44319_2025_672_MOESM6_ESM.zip › Figure4/E/tj_CrebA RNAi1-stage9.tif]

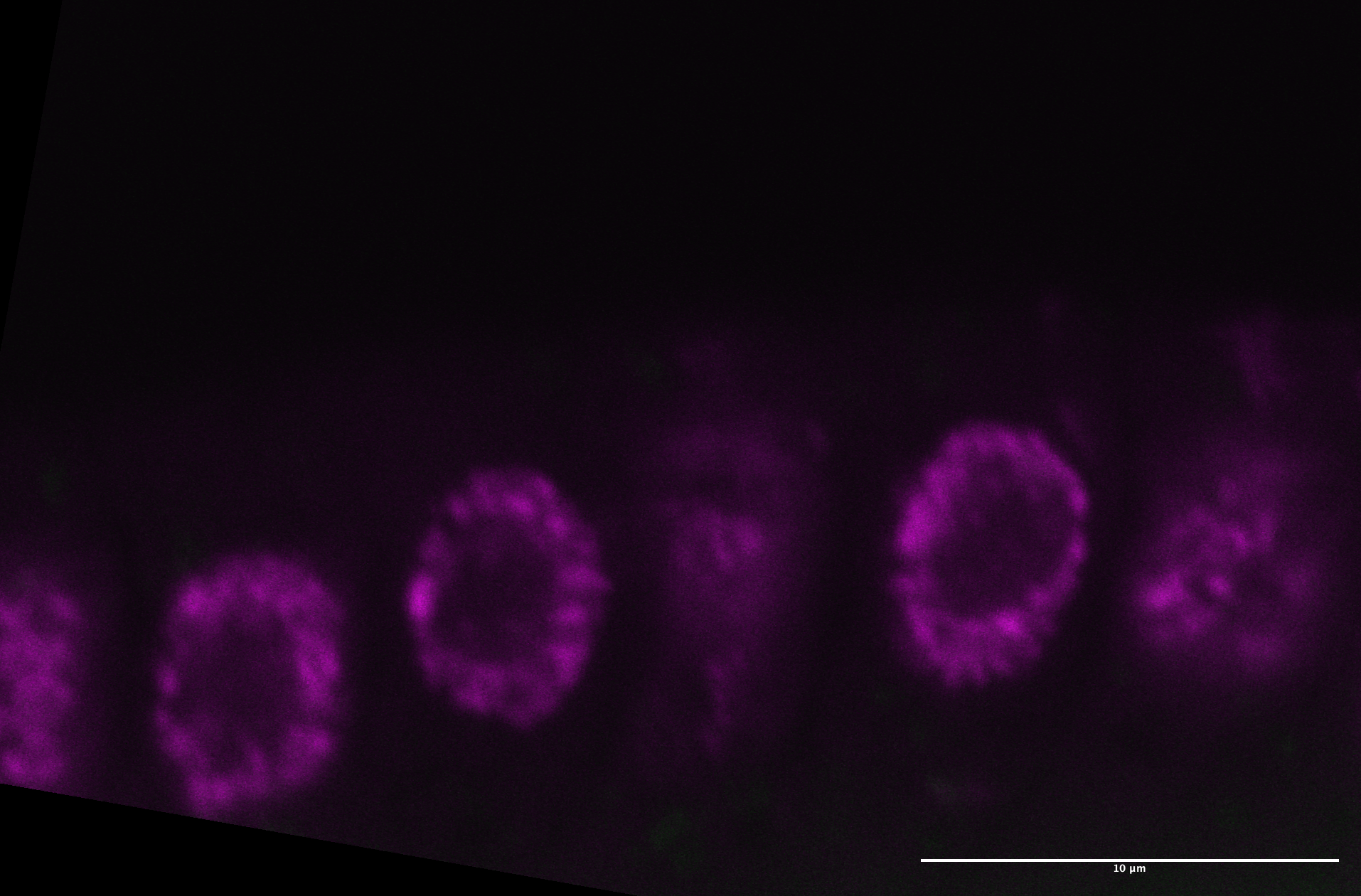

Supplement: Supplementary file 6 — Source data Fig. 4 [file 44319_2025_672_MOESM6_ESM.zip › Figure4/E/tj_CrebA RNAi1-stage9-magnified.tif]

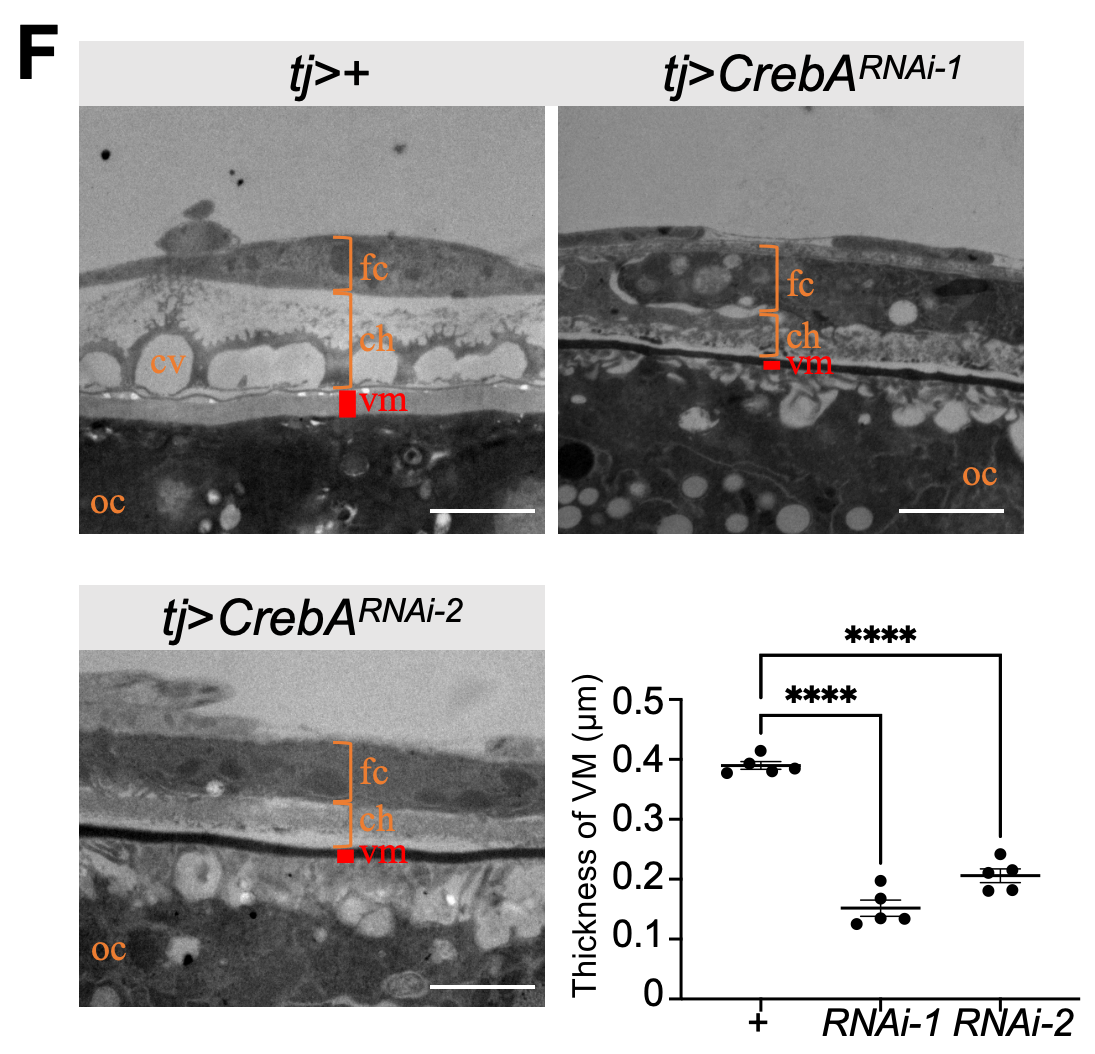

Supplement: Supplementary file 6 — Source data Fig. 4 [file 44319_2025_672_MOESM6_ESM.zip › Figure4/F/F.tif]

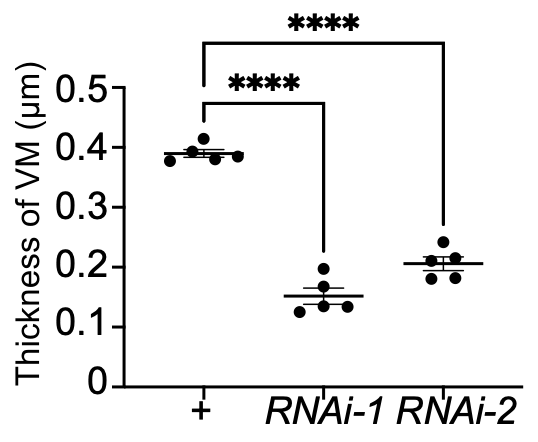

Supplement: Supplementary file 6 — Source data Fig. 4 [file 44319_2025_672_MOESM6_ESM.zip › Figure4/F/q.tif]

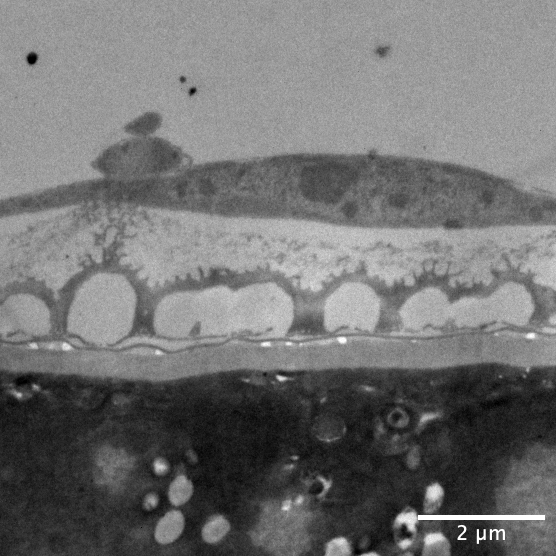

Supplement: Supplementary file 6 — Source data Fig. 4 [file 44319_2025_672_MOESM6_ESM.zip › Figure4/F/tj_+.tif]

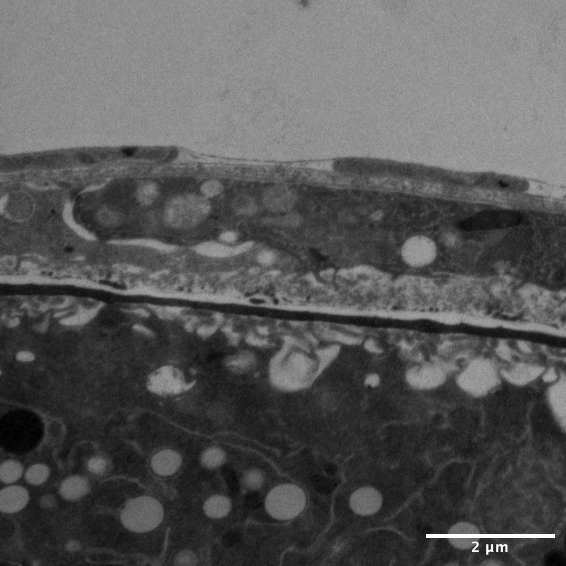

Supplement: Supplementary file 6 — Source data Fig. 4 [file 44319_2025_672_MOESM6_ESM.zip › Figure4/F/tj_CrebA RNAi1.tif]

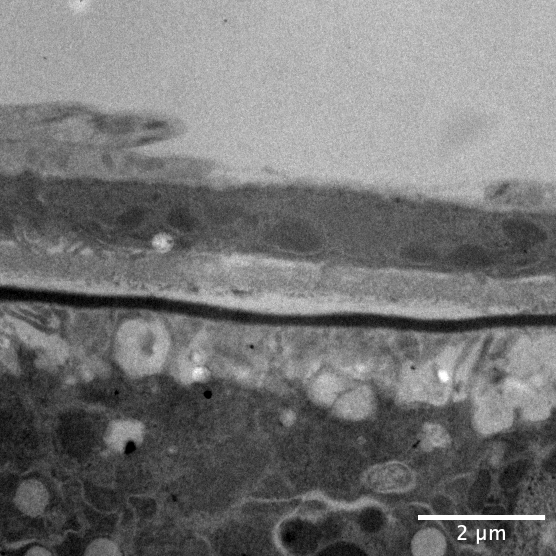

Supplement: Supplementary file 6 — Source data Fig. 4 [file 44319_2025_672_MOESM6_ESM.zip › Figure4/F/tj_CrebA RNAi2.tif]

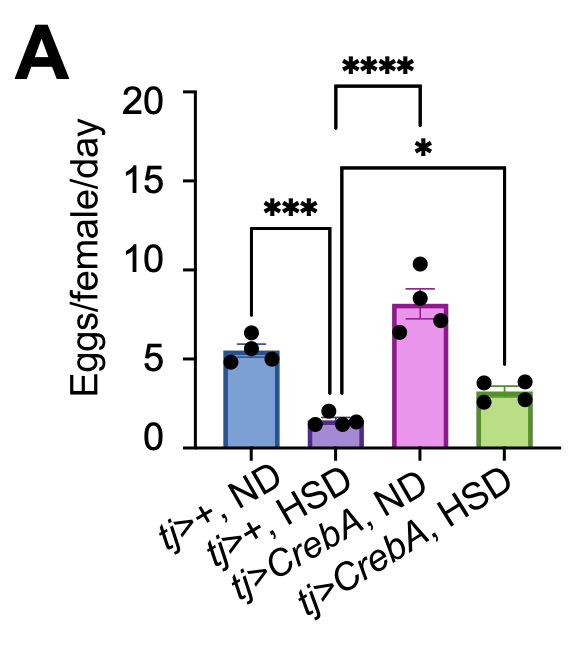

Supplement: Supplementary file 7 — Source data Fig. 5 [file 44319_2025_672_MOESM7_ESM.zip › Figure5/A/A.tif]

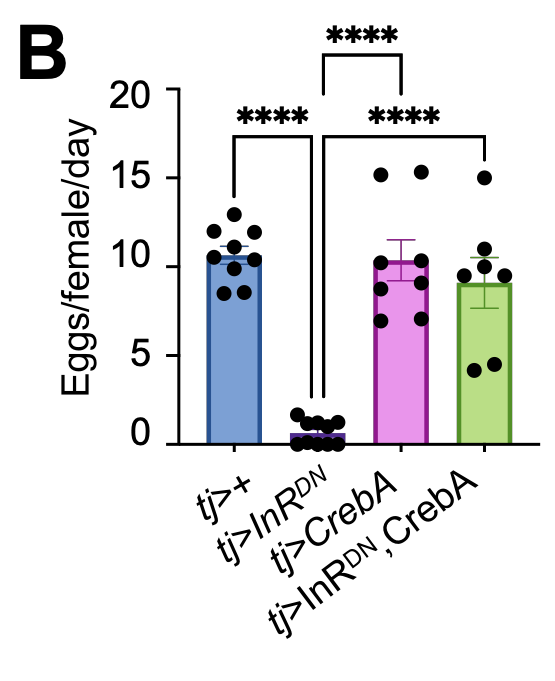

Supplement: Supplementary file 7 — Source data Fig. 5 [file 44319_2025_672_MOESM7_ESM.zip › Figure5/B/B.tif]

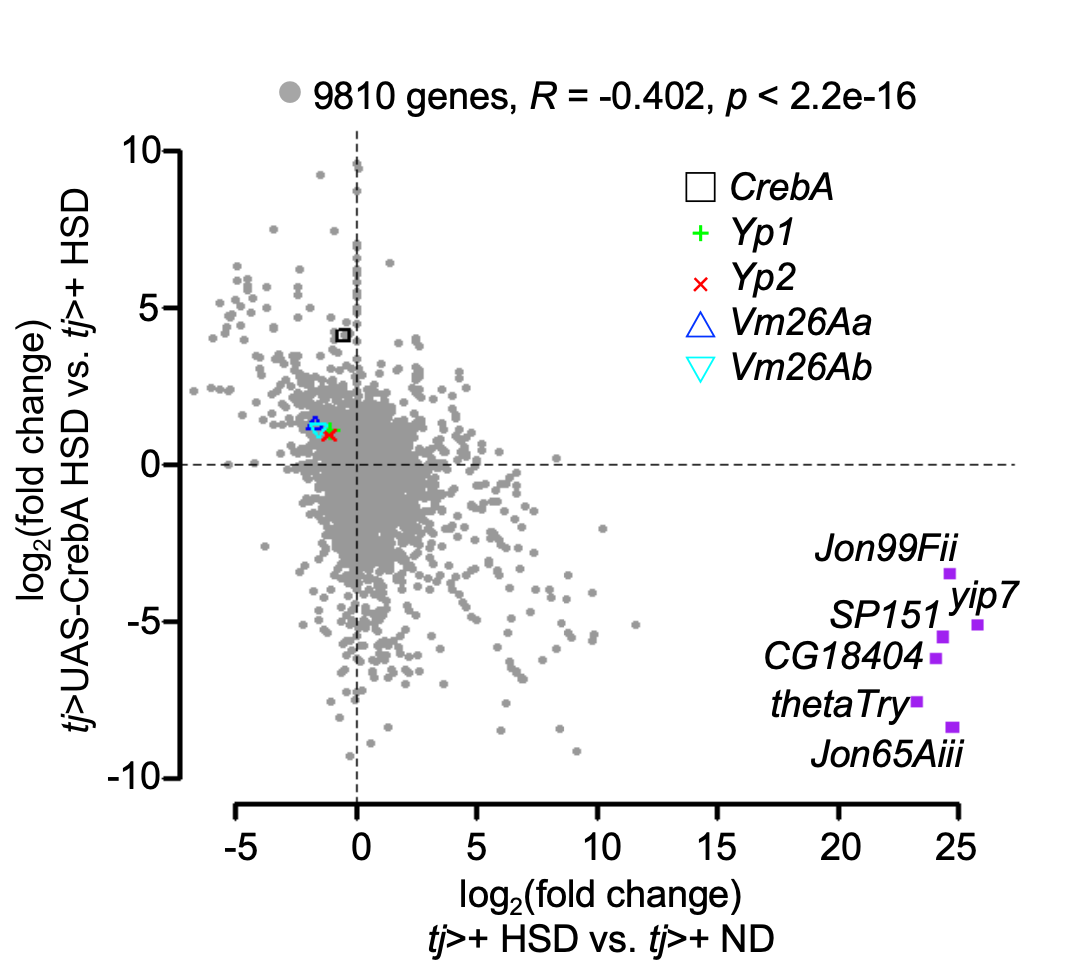

Supplement: Supplementary file 7 — Source data Fig. 5 [file 44319_2025_672_MOESM7_ESM.zip › Figure5/C/C.tif]

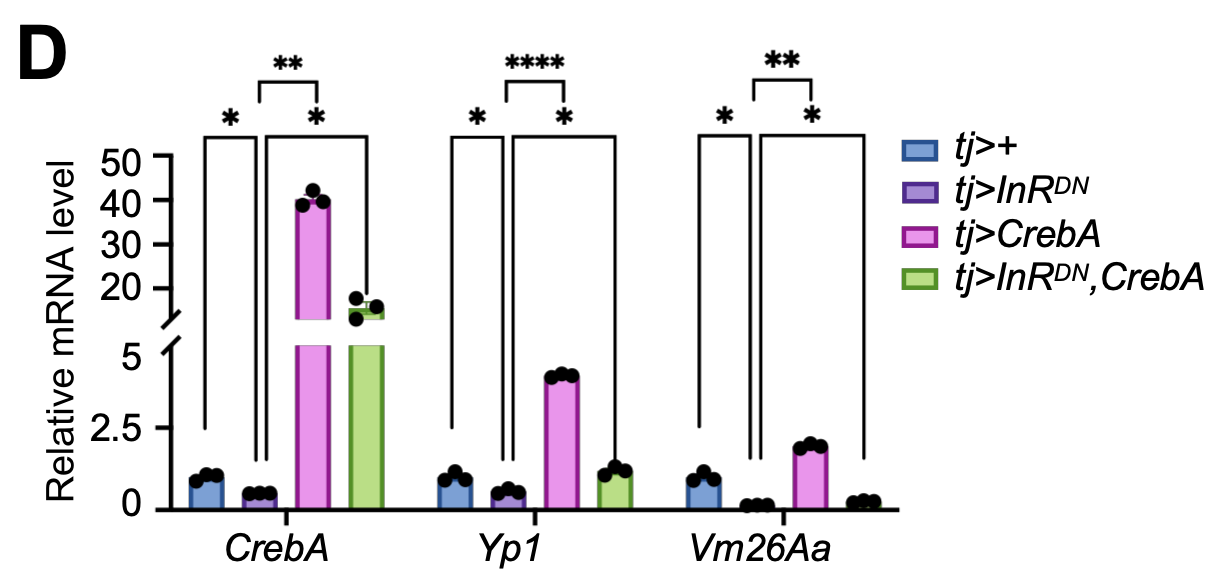

Supplement: Supplementary file 7 — Source data Fig. 5 [file 44319_2025_672_MOESM7_ESM.zip › Figure5/D/D.tif]

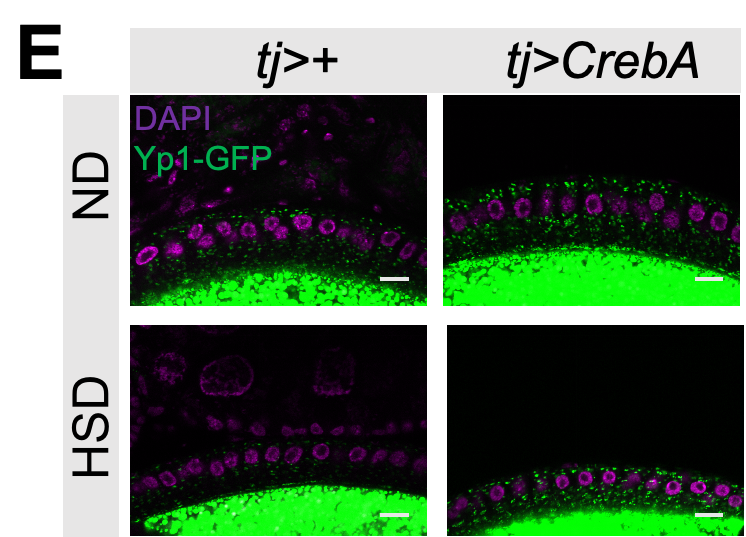

Supplement: Supplementary file 7 — Source data Fig. 5 [file 44319_2025_672_MOESM7_ESM.zip › Figure5/E/E.tif]

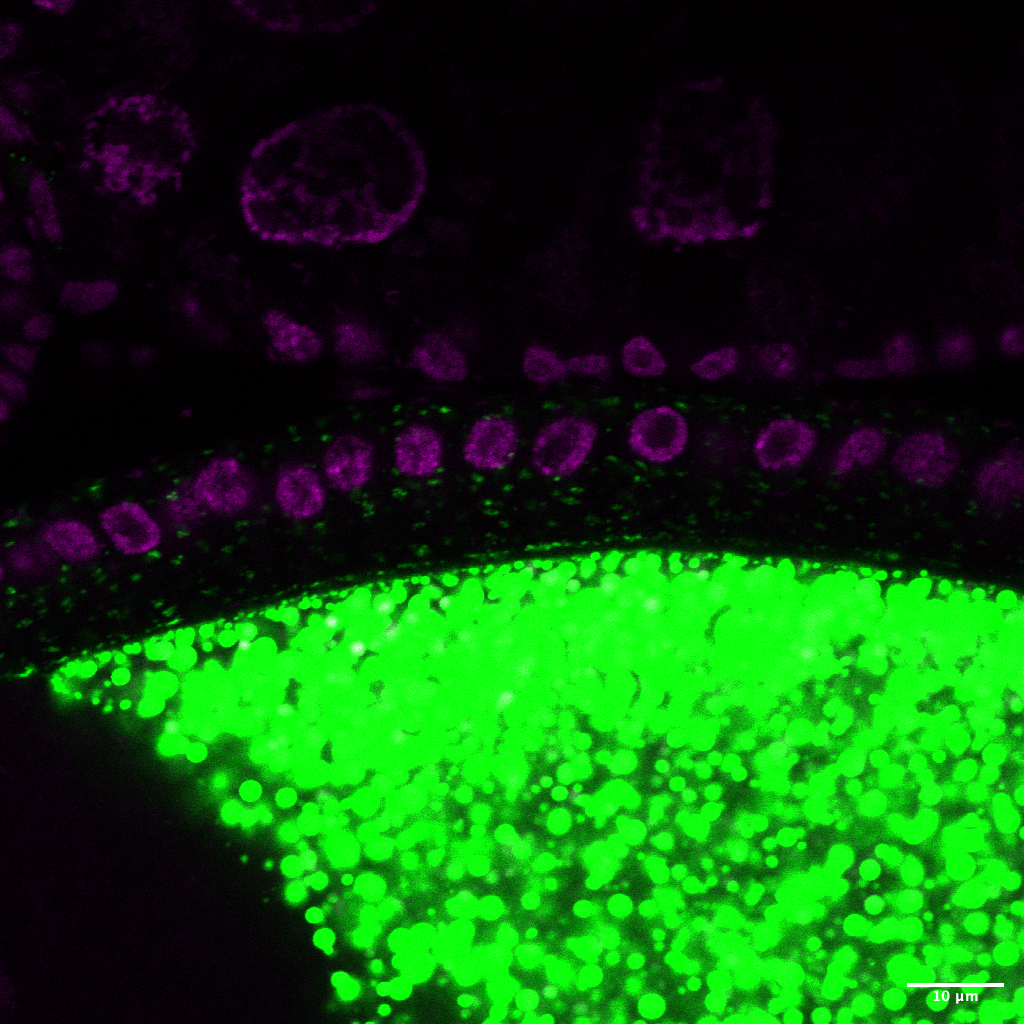

Supplement: Supplementary file 7 — Source data Fig. 5 [file 44319_2025_672_MOESM7_ESM.zip › Figure5/E/HSD-tj_+.tif]

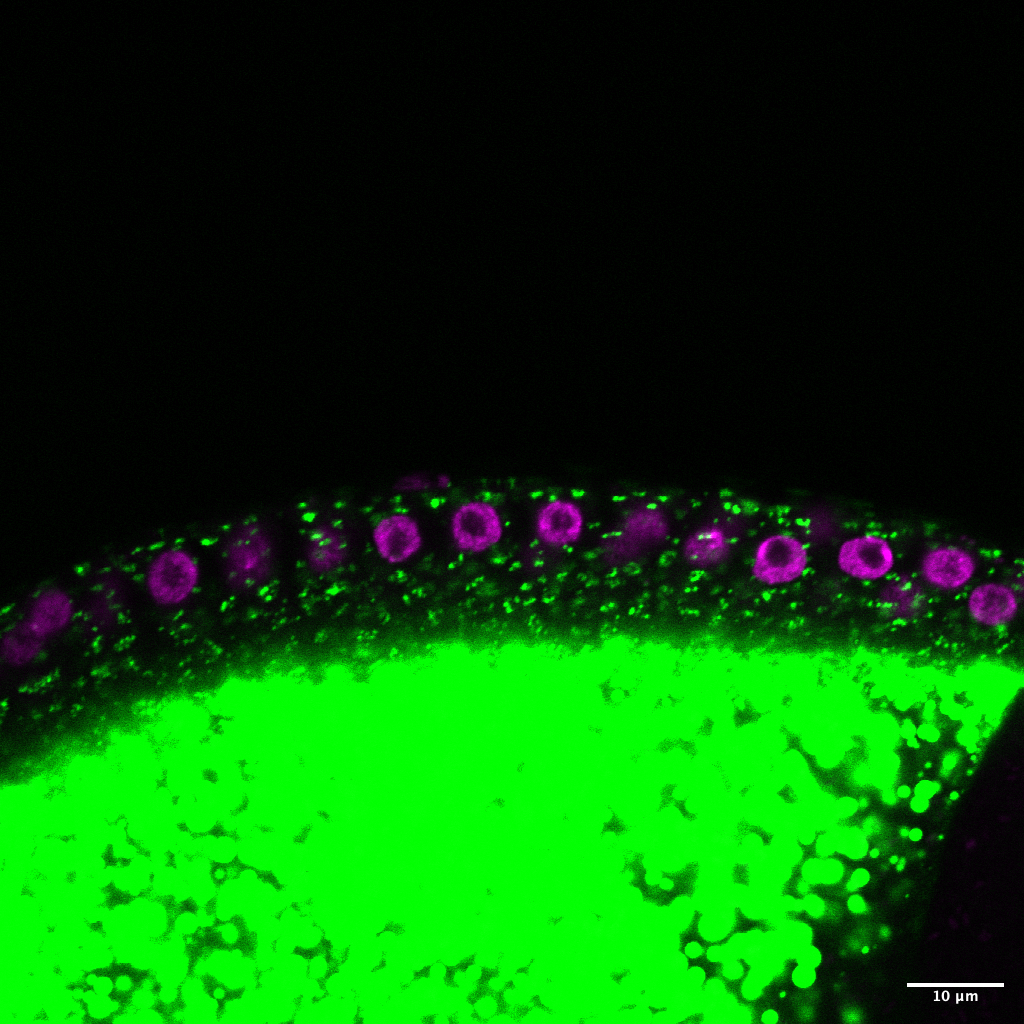

Supplement: Supplementary file 7 — Source data Fig. 5 [file 44319_2025_672_MOESM7_ESM.zip › Figure5/E/HSD-tj_CrebA.tif]

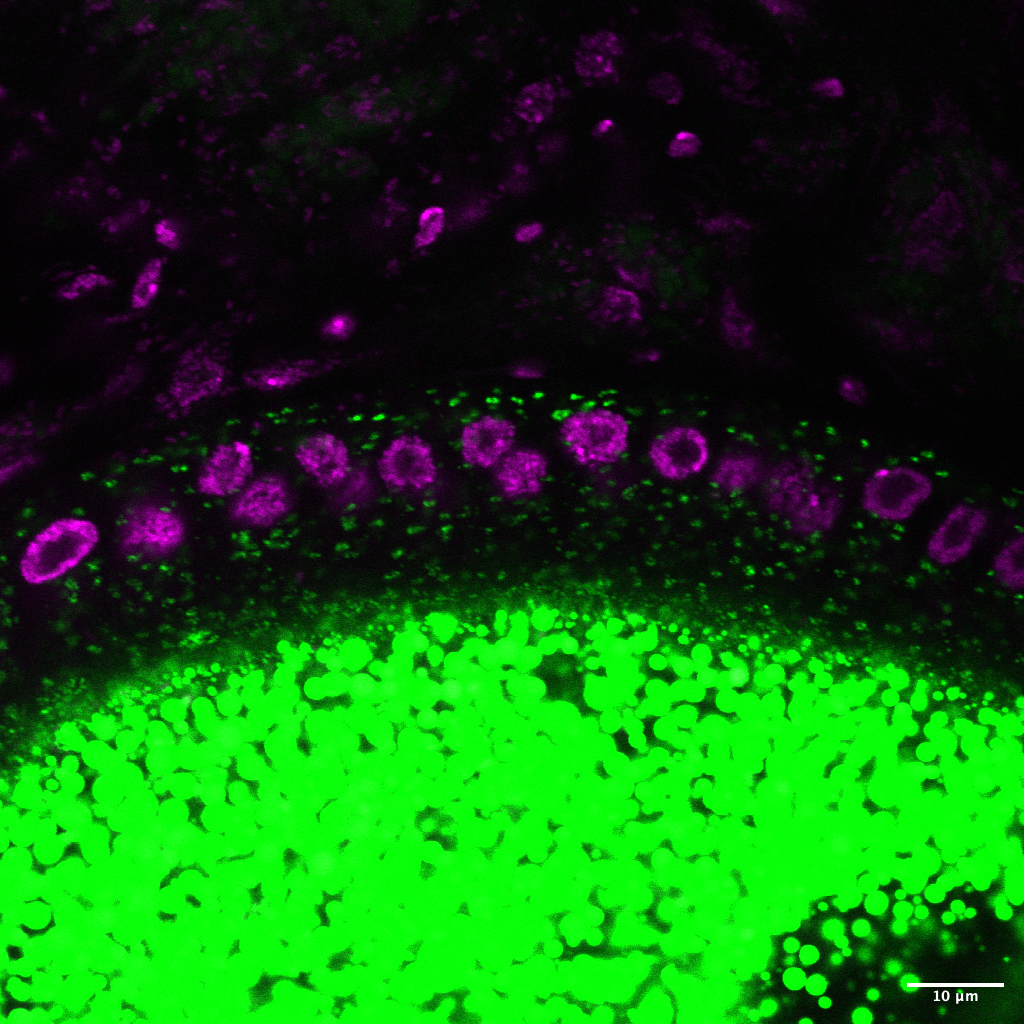

Supplement: Supplementary file 7 — Source data Fig. 5 [file 44319_2025_672_MOESM7_ESM.zip › Figure5/E/ND-tj_+.tif]

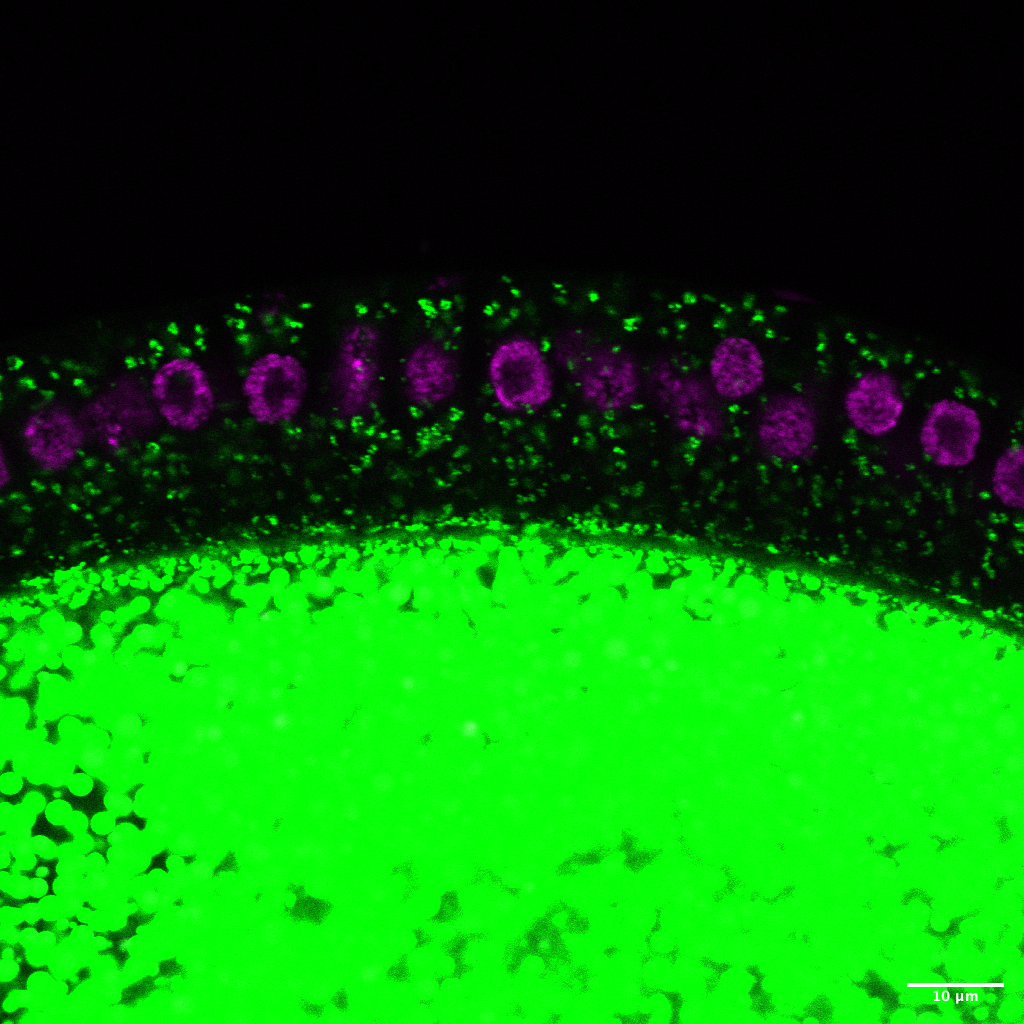

Supplement: Supplementary file 7 — Source data Fig. 5 [file 44319_2025_672_MOESM7_ESM.zip › Figure5/E/ND-tj_CrebA.tif]

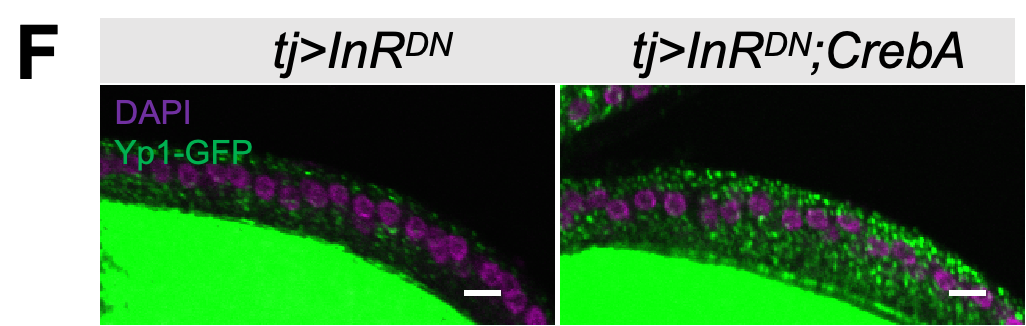

Supplement: Supplementary file 7 — Source data Fig. 5 [file 44319_2025_672_MOESM7_ESM.zip › Figure5/F/F.tif]

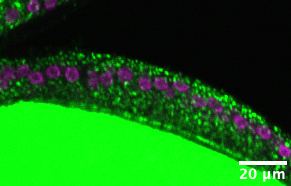

Supplement: Supplementary file 7 — Source data Fig. 5 [file 44319_2025_672_MOESM7_ESM.zip › Figure5/F/tj_InRDN,CrebA.tif]

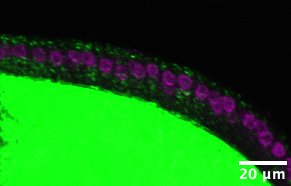

Supplement: Supplementary file 7 — Source data Fig. 5 [file 44319_2025_672_MOESM7_ESM.zip › Figure5/F/tj_InRDN.tif]

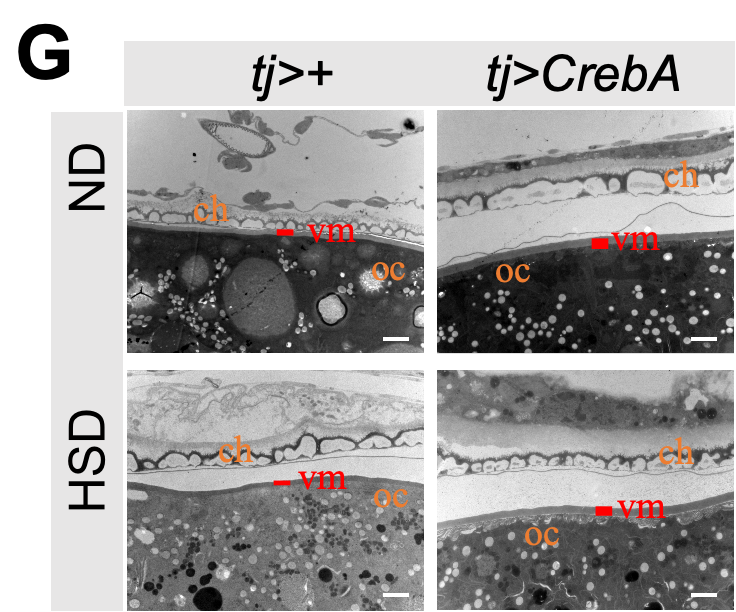

Supplement: Supplementary file 7 — Source data Fig. 5 [file 44319_2025_672_MOESM7_ESM.zip › Figure5/G/G.tif]

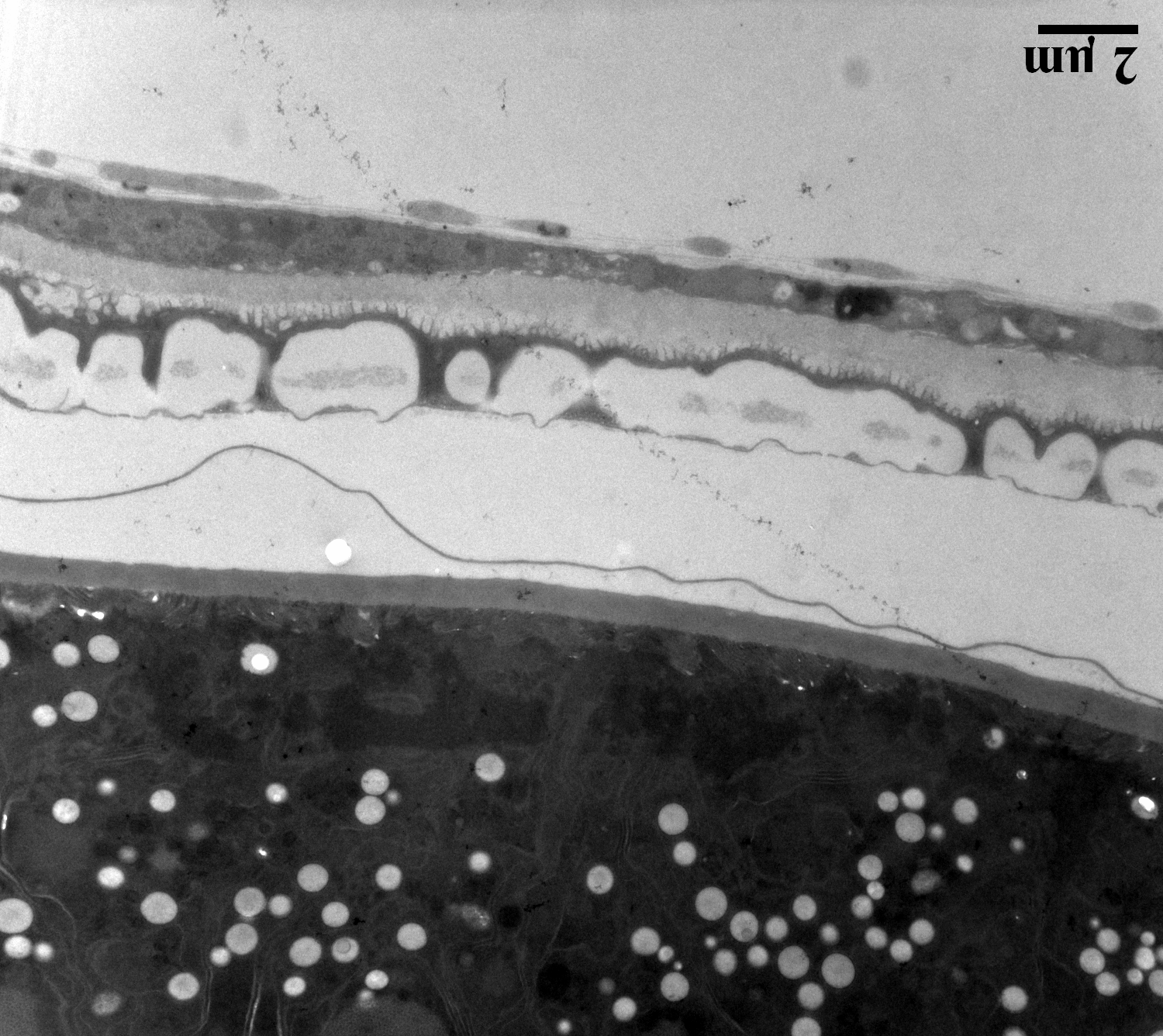

Supplement: Supplementary file 7 — Source data Fig. 5 [file 44319_2025_672_MOESM7_ESM.zip › Figure5/G/ND-tj_CrebA.tif]

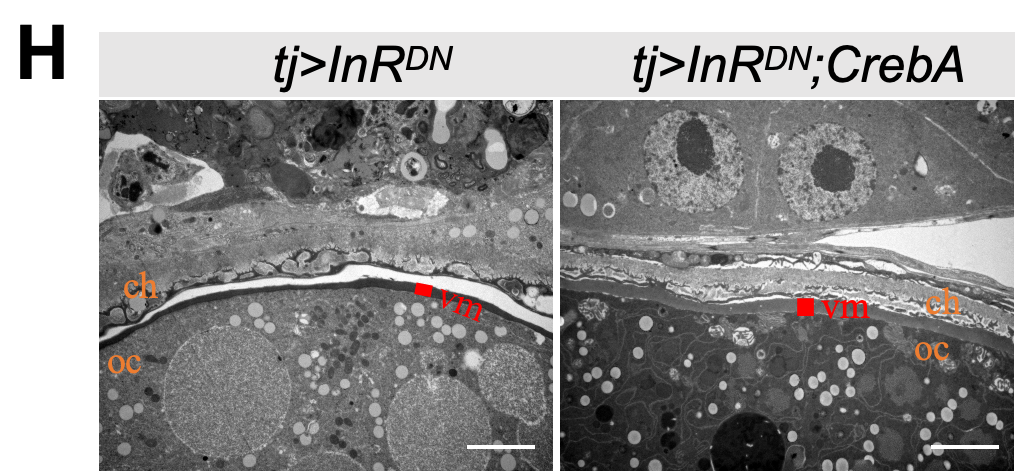

Supplement: Supplementary file 7 — Source data Fig. 5 [file 44319_2025_672_MOESM7_ESM.zip › Figure5/H/H.tif]

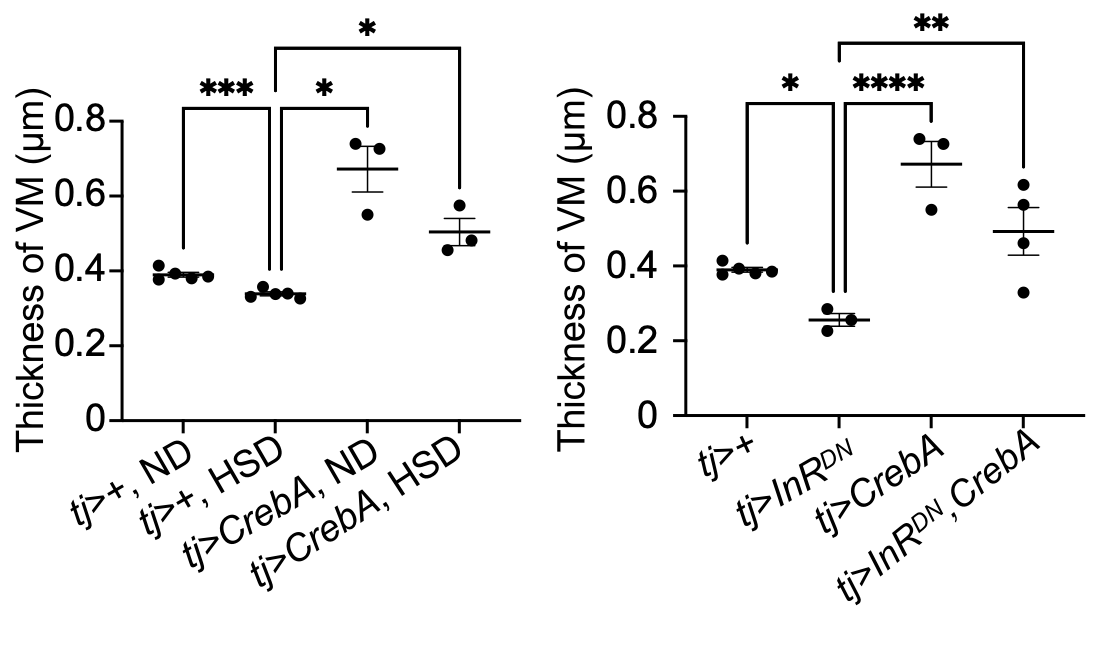

Supplement: Supplementary file 7 — Source data Fig. 5 [file 44319_2025_672_MOESM7_ESM.zip › Figure5/I/I.tif]

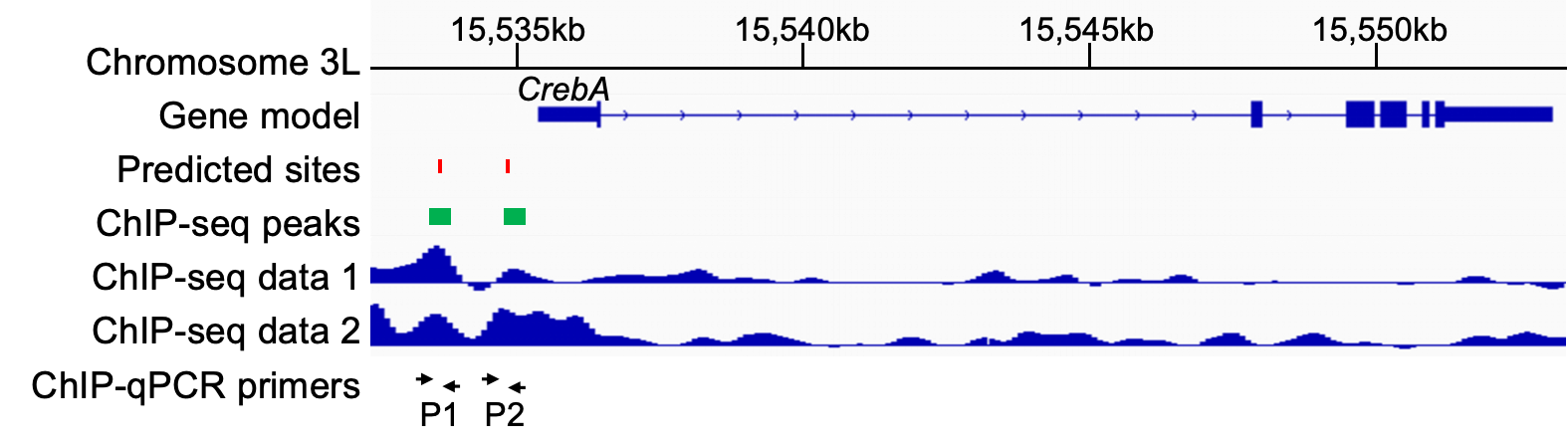

Supplement: Supplementary file 8 — Source data Fig. 6 [file 44319_2025_672_MOESM8_ESM.zip › Figure6/A/A.tif]

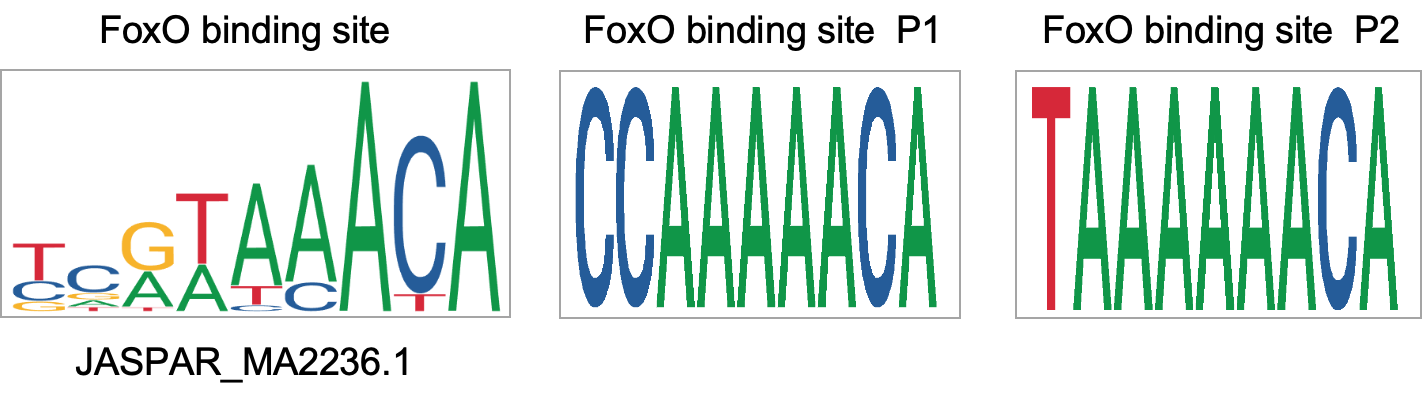

Supplement: Supplementary file 8 — Source data Fig. 6 [file 44319_2025_672_MOESM8_ESM.zip › Figure6/B/B.tif]

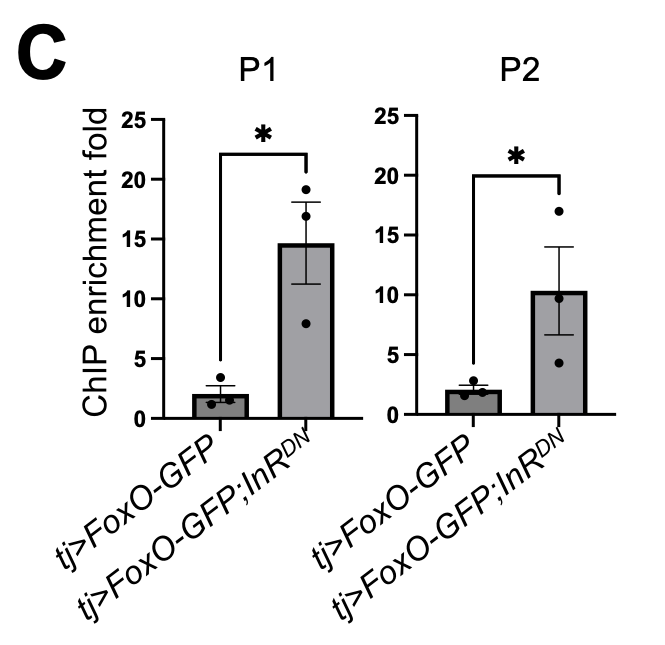

Supplement: Supplementary file 8 — Source data Fig. 6 [file 44319_2025_672_MOESM8_ESM.zip › Figure6/C/C.tif]

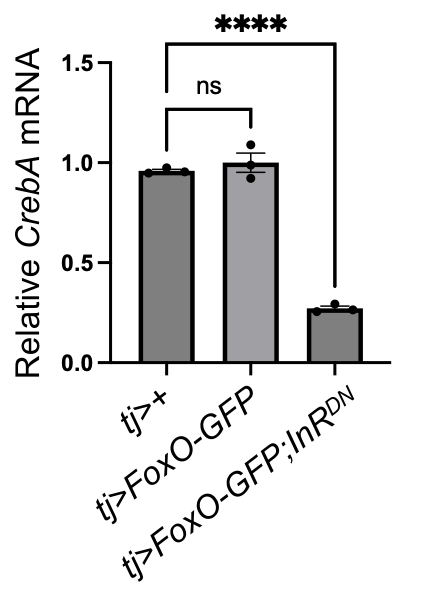

Supplement: Supplementary file 8 — Source data Fig. 6 [file 44319_2025_672_MOESM8_ESM.zip › Figure6/D/D.tif]

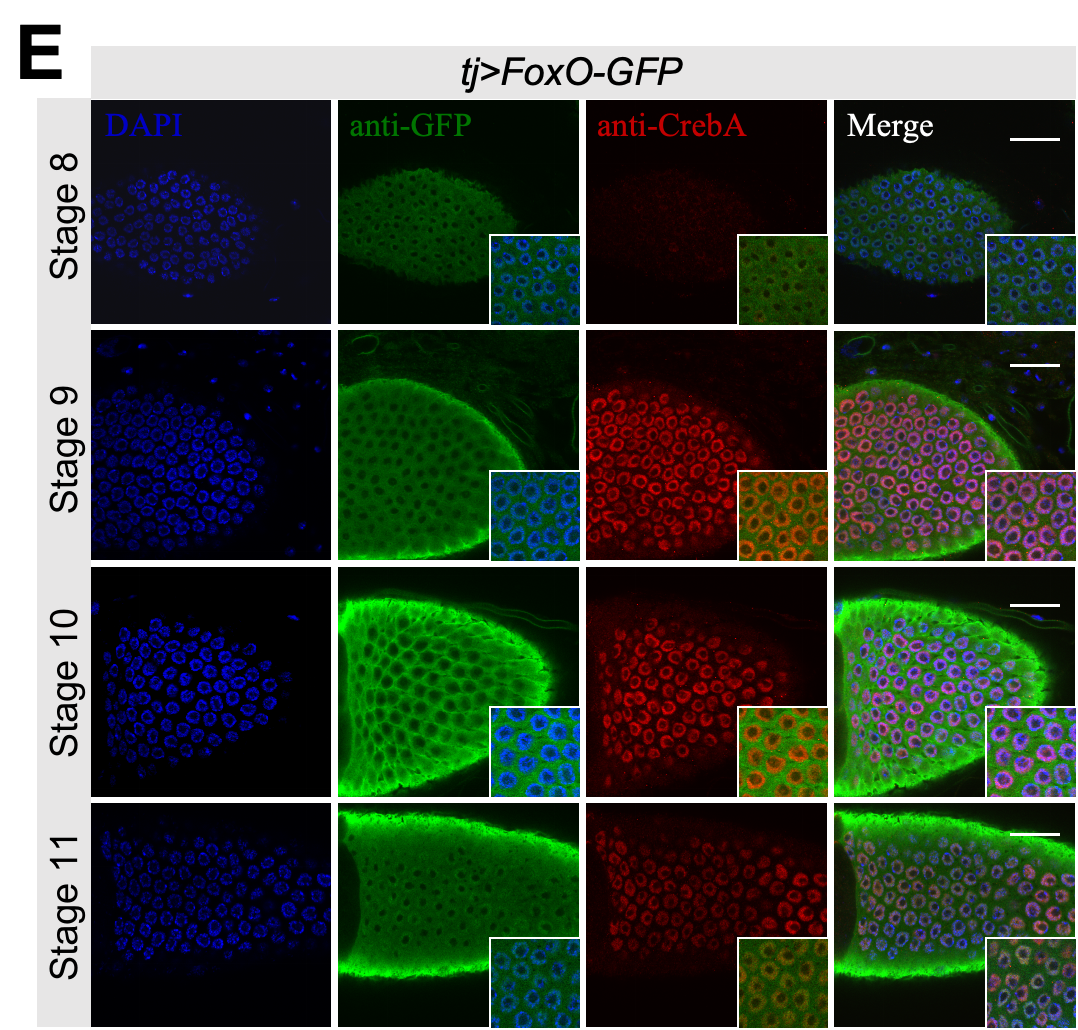

Supplement: Supplementary file 8 — Source data Fig. 6 [file 44319_2025_672_MOESM8_ESM.zip › Figure6/E/Etif.tif]

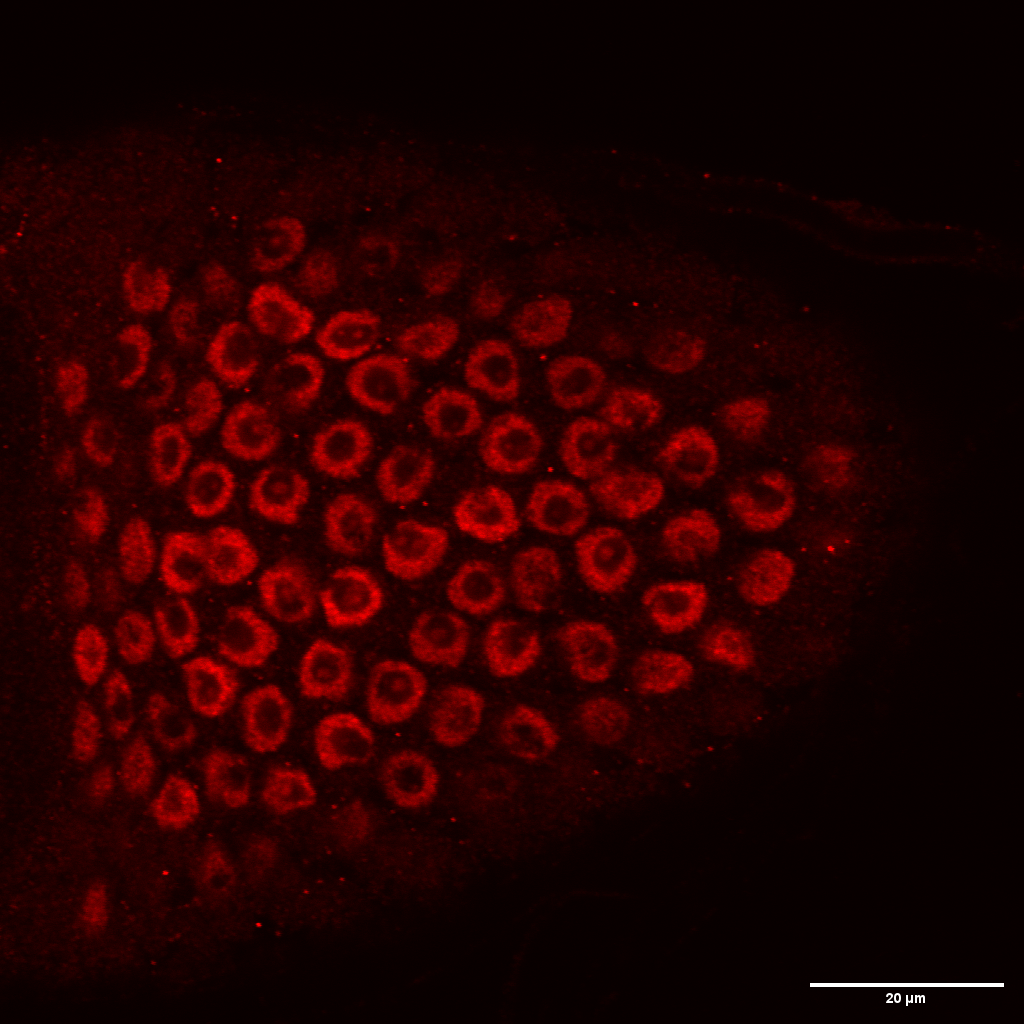

Supplement: Supplementary file 8 — Source data Fig. 6 [file 44319_2025_672_MOESM8_ESM.zip › Figure6/E/stage10-antiCrebA.tif]

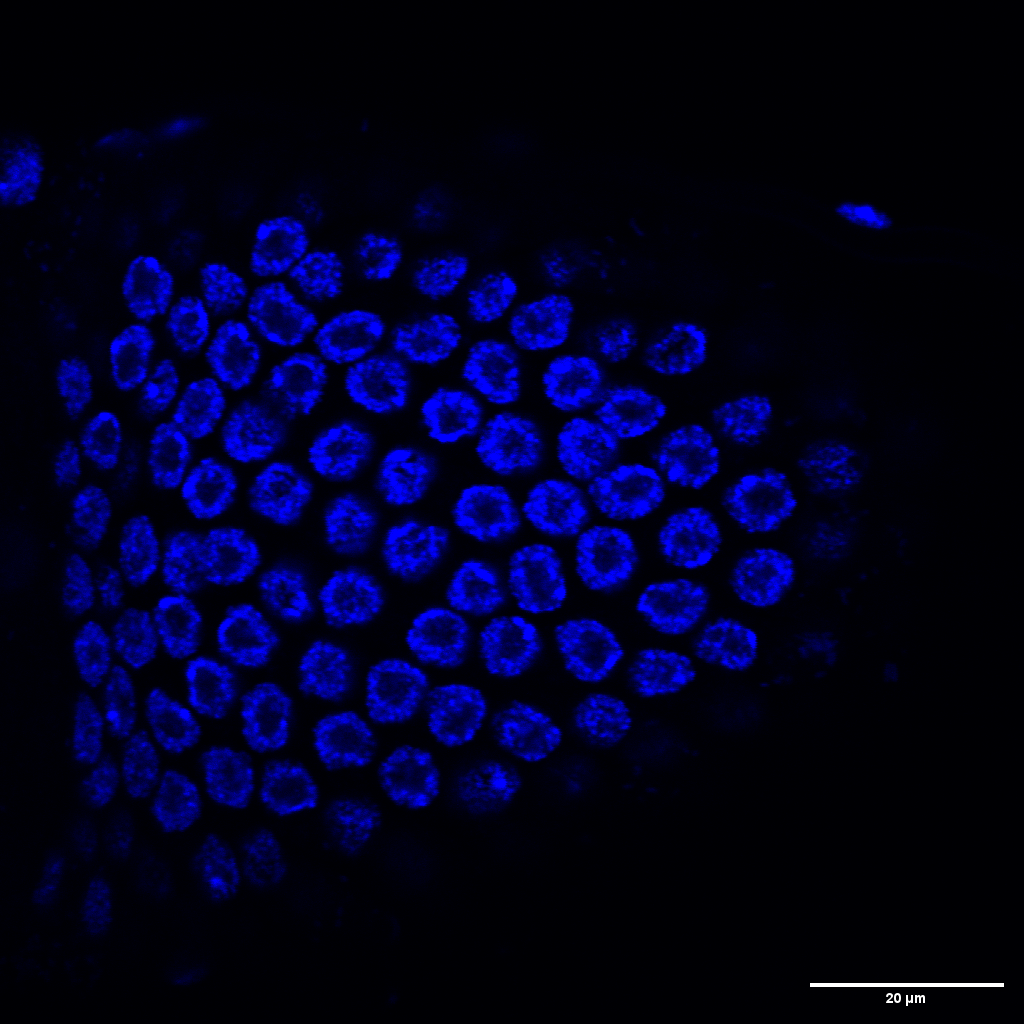

Supplement: Supplementary file 8 — Source data Fig. 6 [file 44319_2025_672_MOESM8_ESM.zip › Figure6/E/stage10-DAPI.tif]

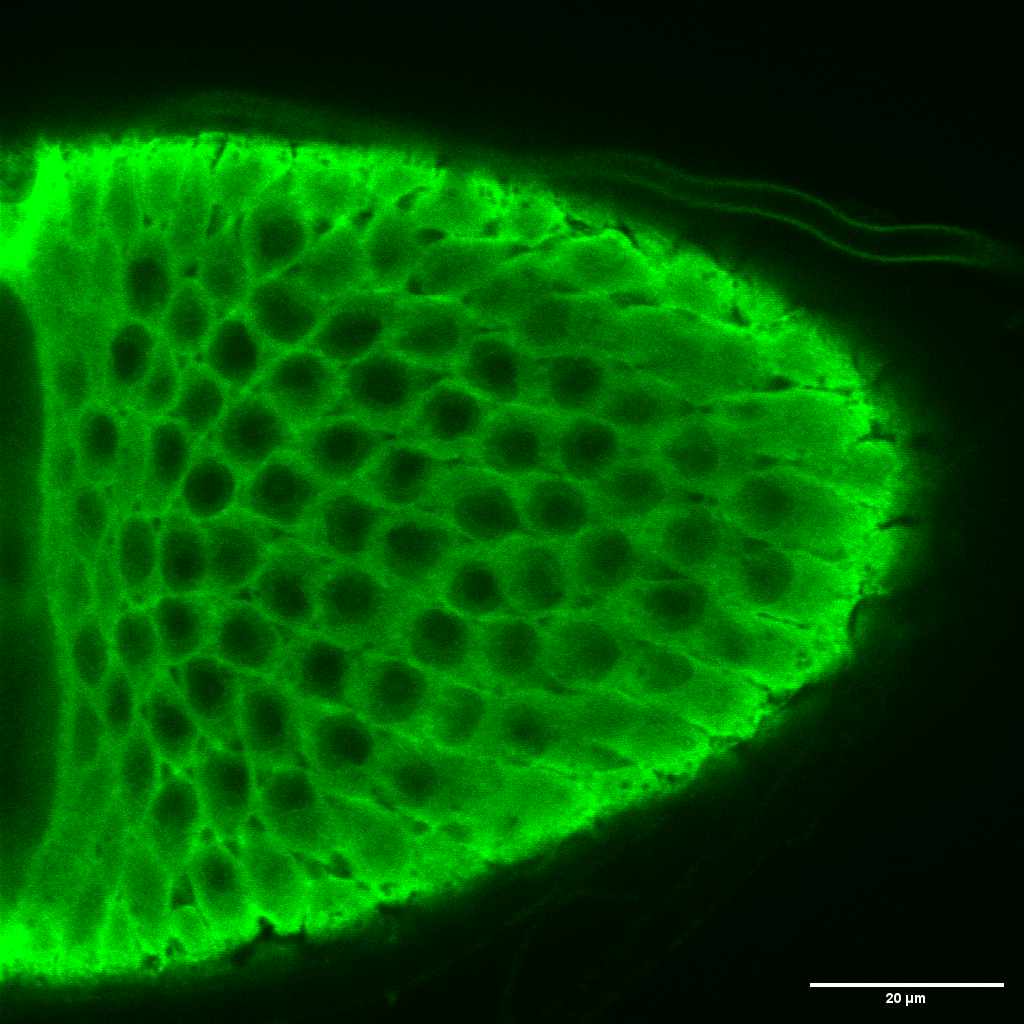

Supplement: Supplementary file 8 — Source data Fig. 6 [file 44319_2025_672_MOESM8_ESM.zip › Figure6/E/stage10-GFP.tif]

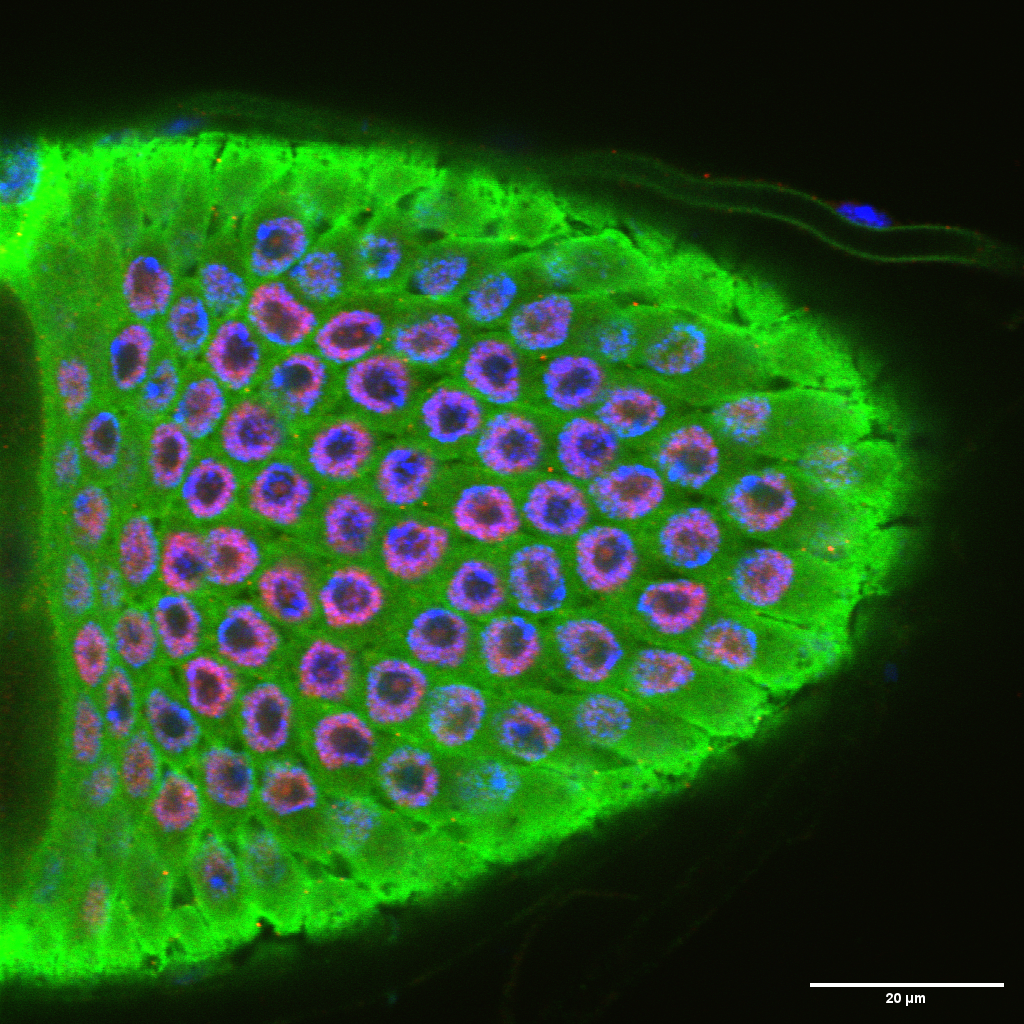

Supplement: Supplementary file 8 — Source data Fig. 6 [file 44319_2025_672_MOESM8_ESM.zip › Figure6/E/stage10-Merge.tif]

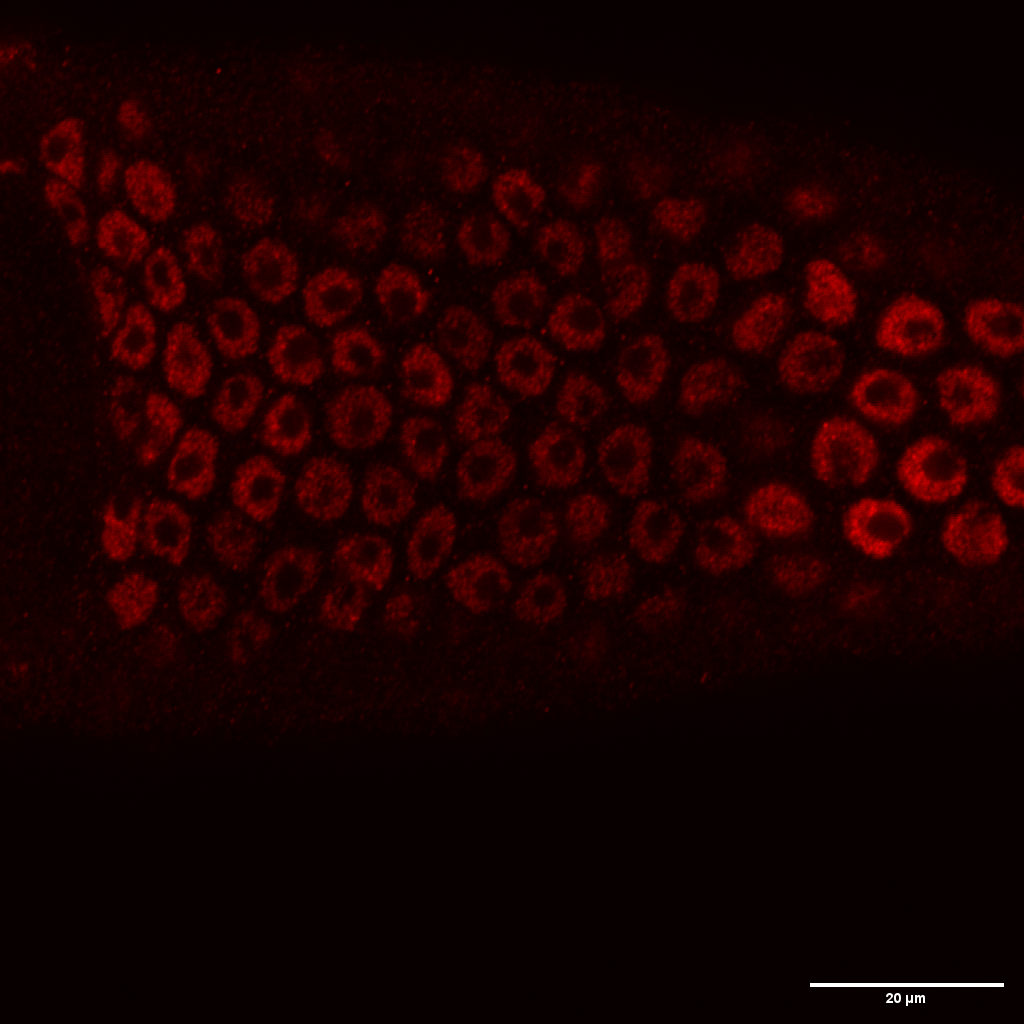

Supplement: Supplementary file 8 — Source data Fig. 6 [file 44319_2025_672_MOESM8_ESM.zip › Figure6/E/stage11-antiCrebA.tif]

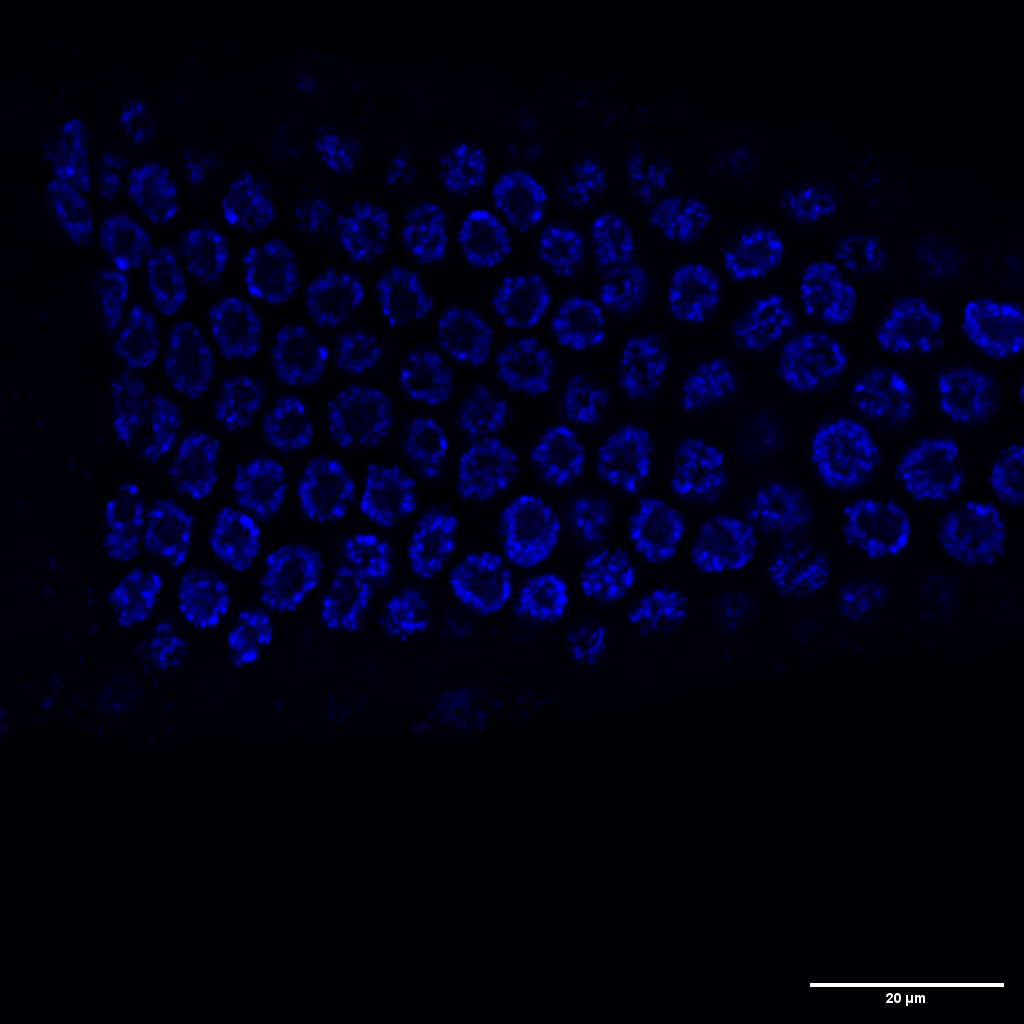

Supplement: Supplementary file 8 — Source data Fig. 6 [file 44319_2025_672_MOESM8_ESM.zip › Figure6/E/stage11-DAPI.tif]

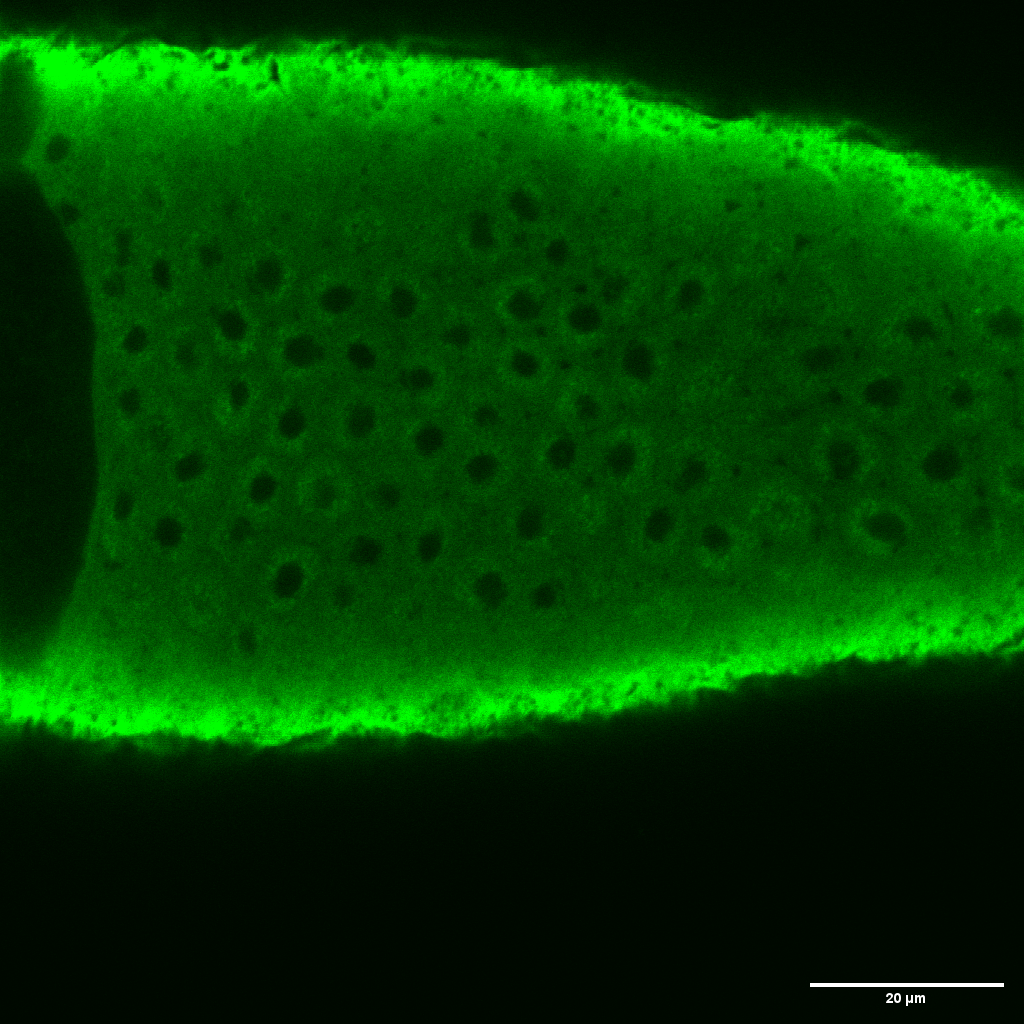

Supplement: Supplementary file 8 — Source data Fig. 6 [file 44319_2025_672_MOESM8_ESM.zip › Figure6/E/stage11-GFP.tif]

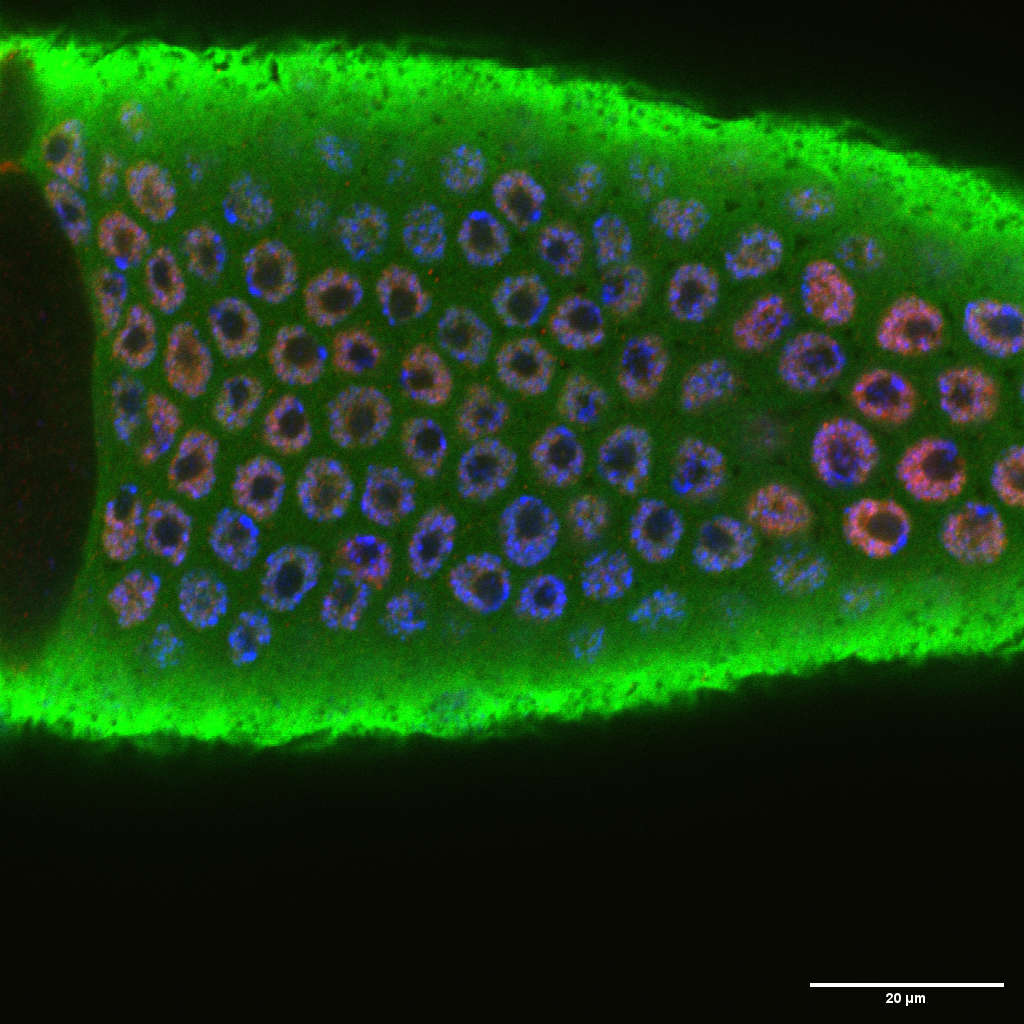

Supplement: Supplementary file 8 — Source data Fig. 6 [file 44319_2025_672_MOESM8_ESM.zip › Figure6/E/stage11-Merge.tif]

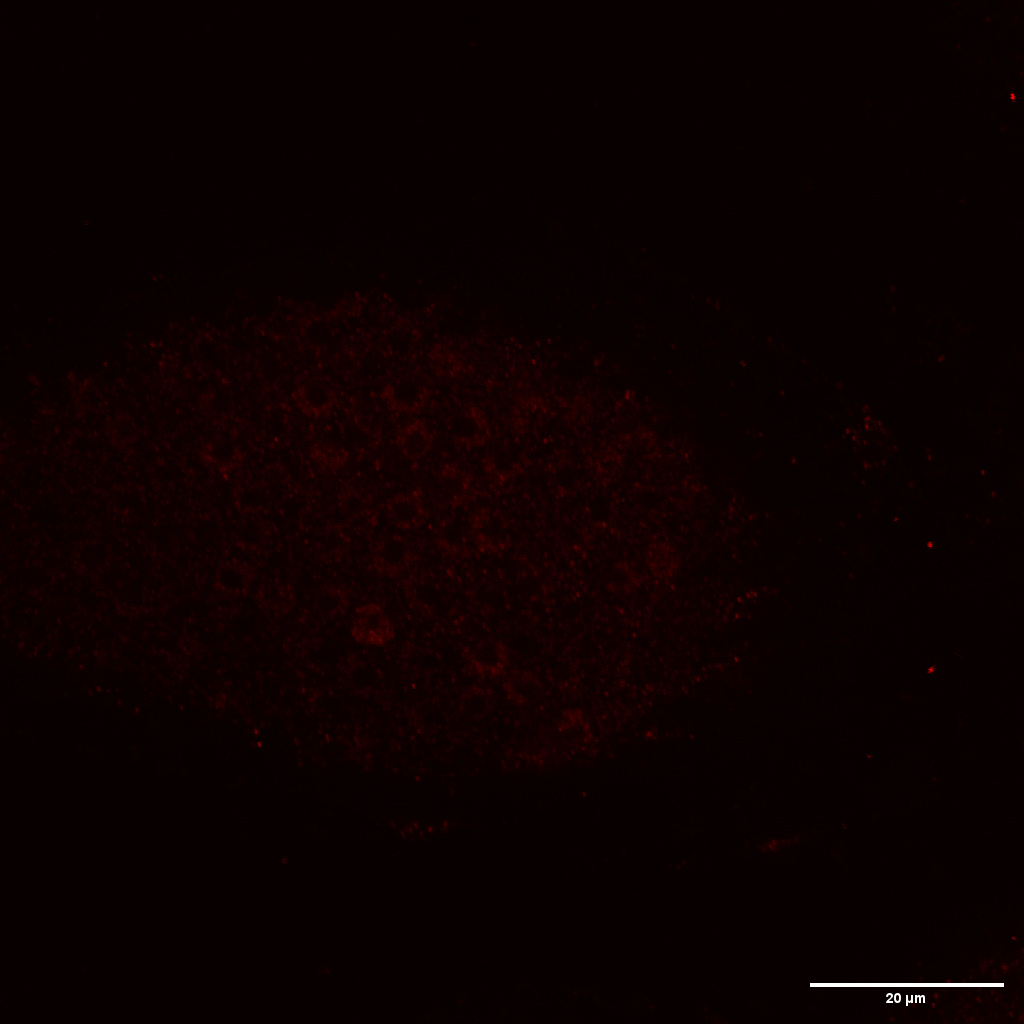

Supplement: Supplementary file 8 — Source data Fig. 6 [file 44319_2025_672_MOESM8_ESM.zip › Figure6/E/stage8-antiCrebA.tif]

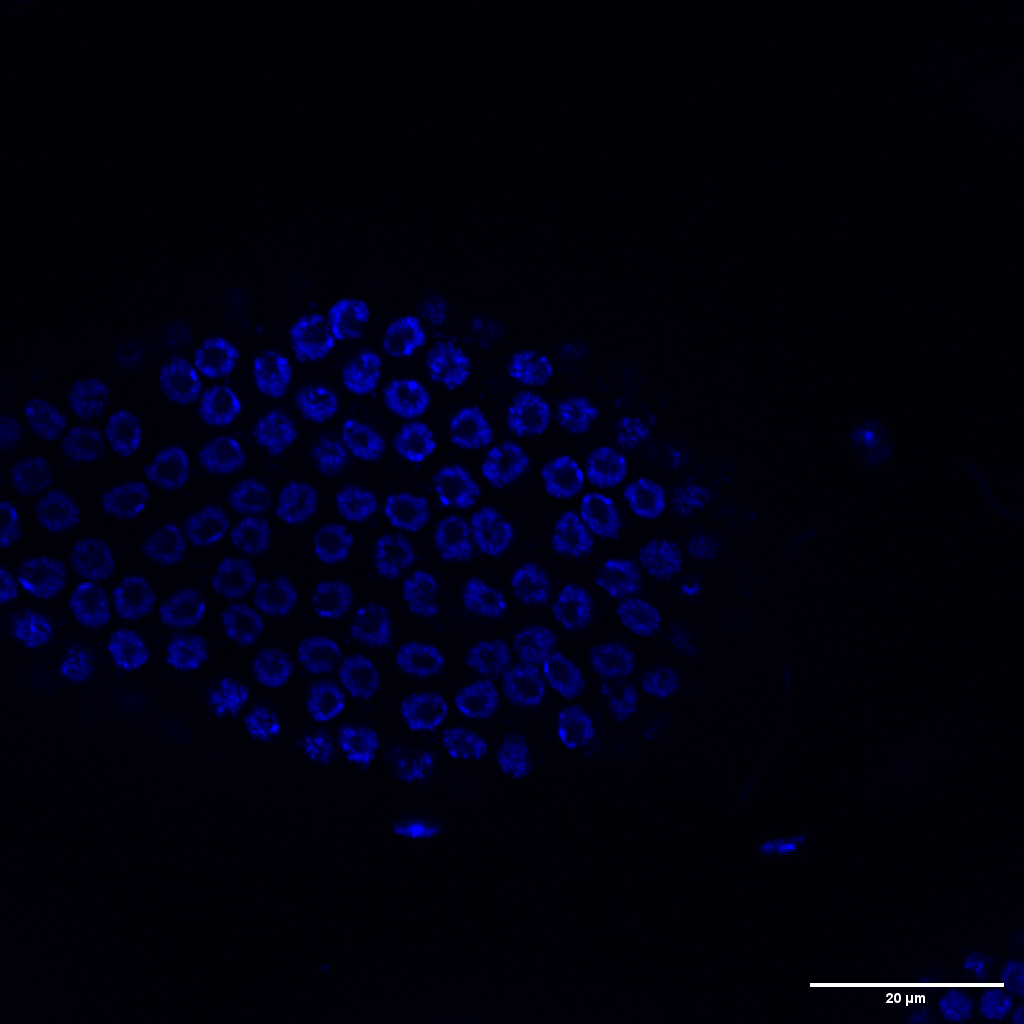

Supplement: Supplementary file 8 — Source data Fig. 6 [file 44319_2025_672_MOESM8_ESM.zip › Figure6/E/stage8-DAPI.tif]

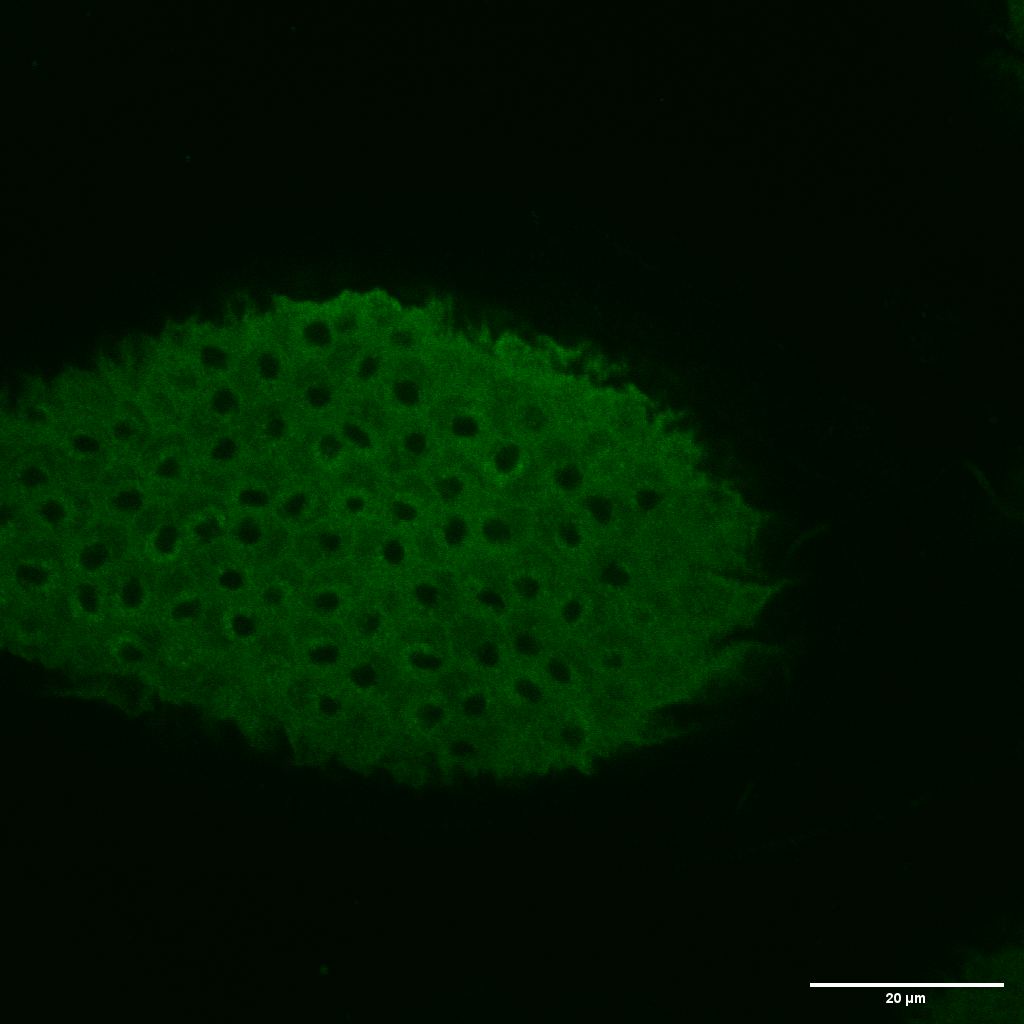

Supplement: Supplementary file 8 — Source data Fig. 6 [file 44319_2025_672_MOESM8_ESM.zip › Figure6/E/stage8-GFP.tif]

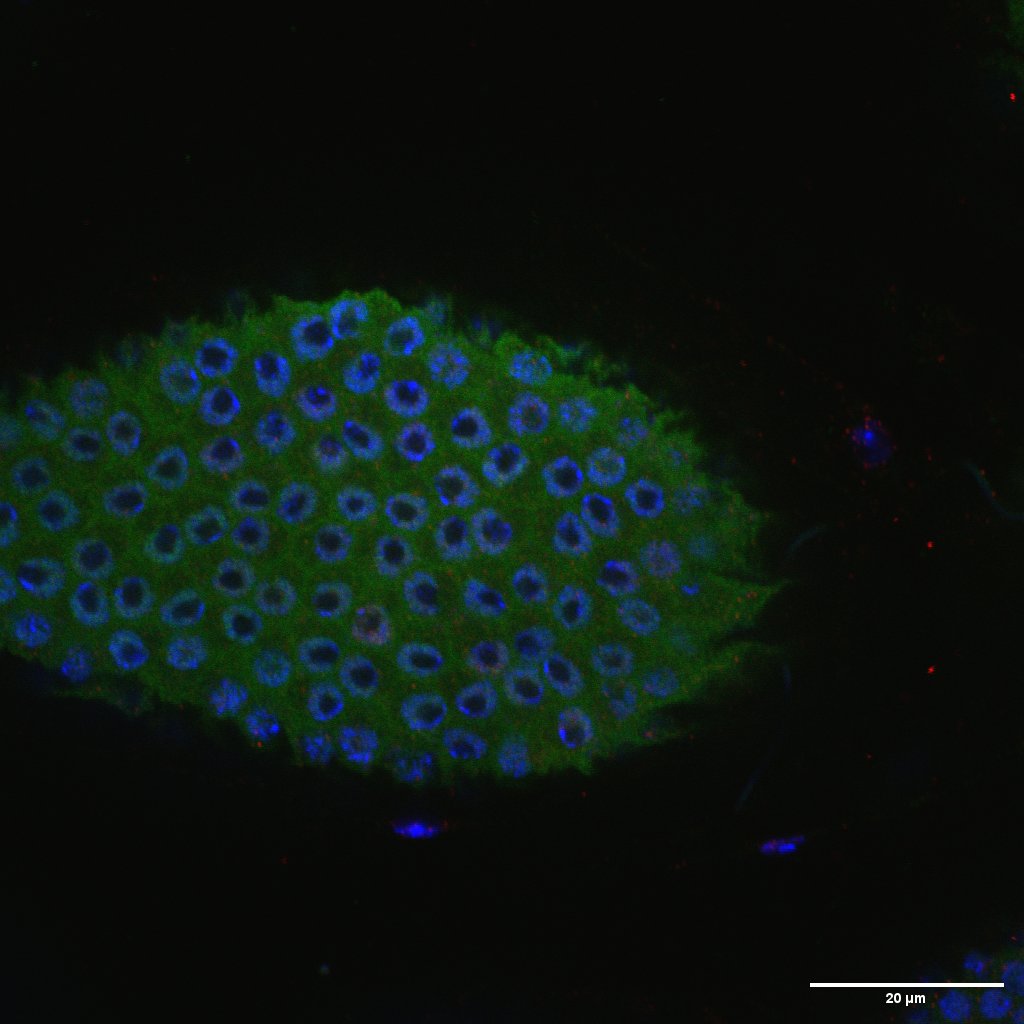

Supplement: Supplementary file 8 — Source data Fig. 6 [file 44319_2025_672_MOESM8_ESM.zip › Figure6/E/stage8-Merge.tif]

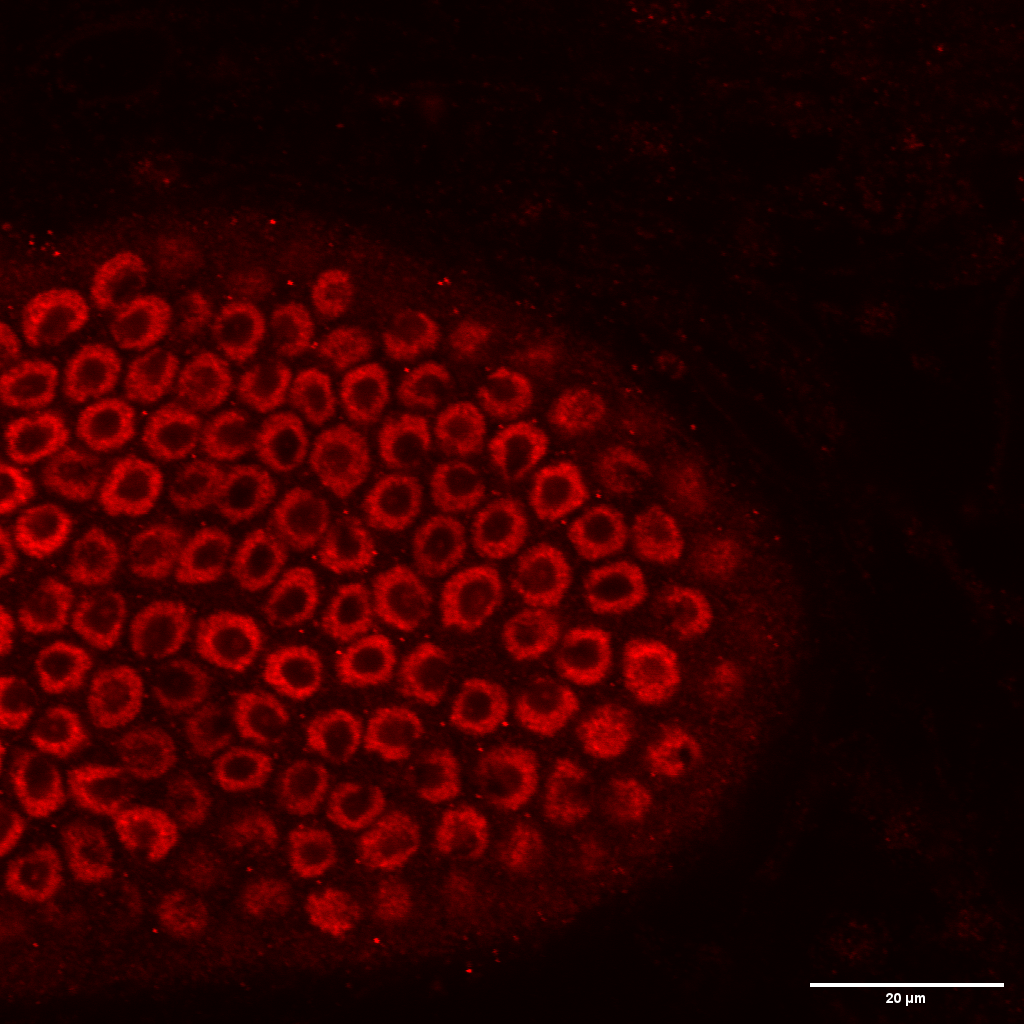

Supplement: Supplementary file 8 — Source data Fig. 6 [file 44319_2025_672_MOESM8_ESM.zip › Figure6/E/stage9-antiCrebA.tif]

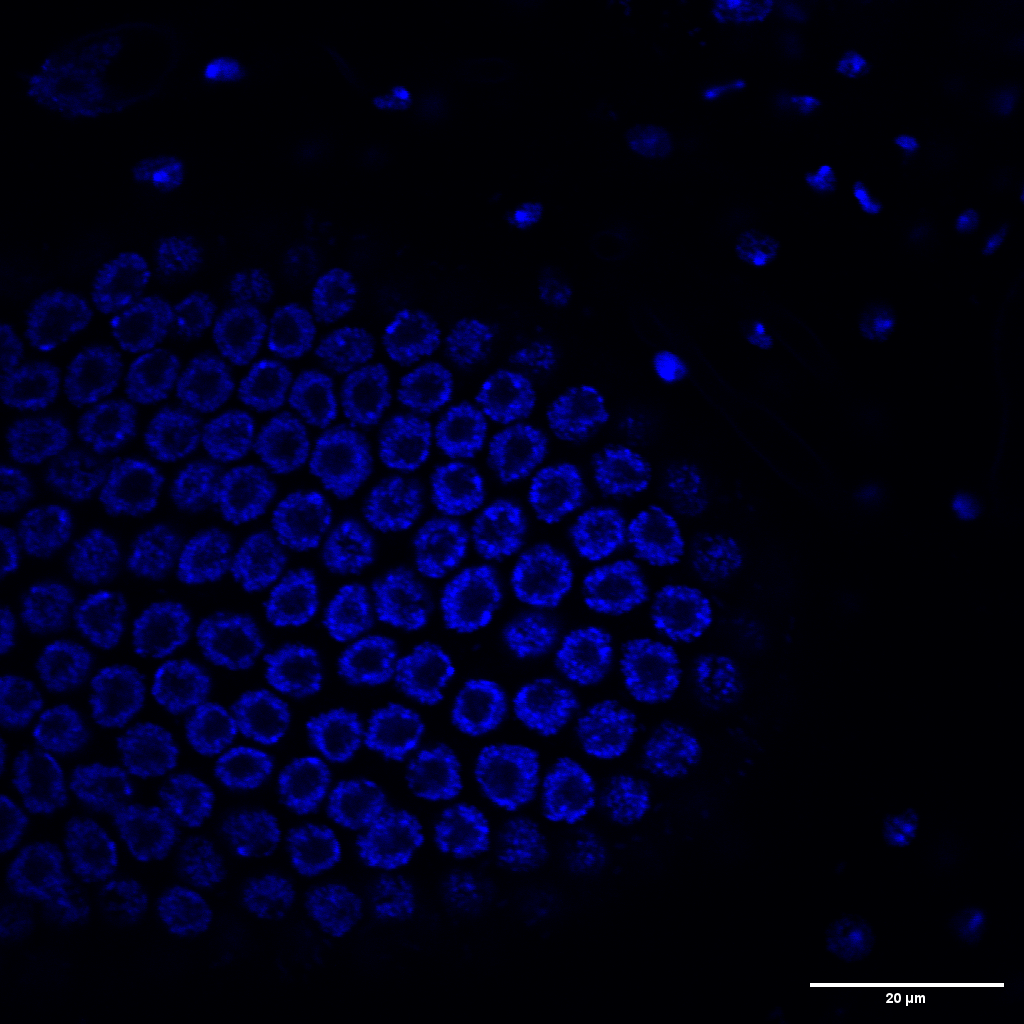

Supplement: Supplementary file 8 — Source data Fig. 6 [file 44319_2025_672_MOESM8_ESM.zip › Figure6/E/stage9-DAPI.tif]

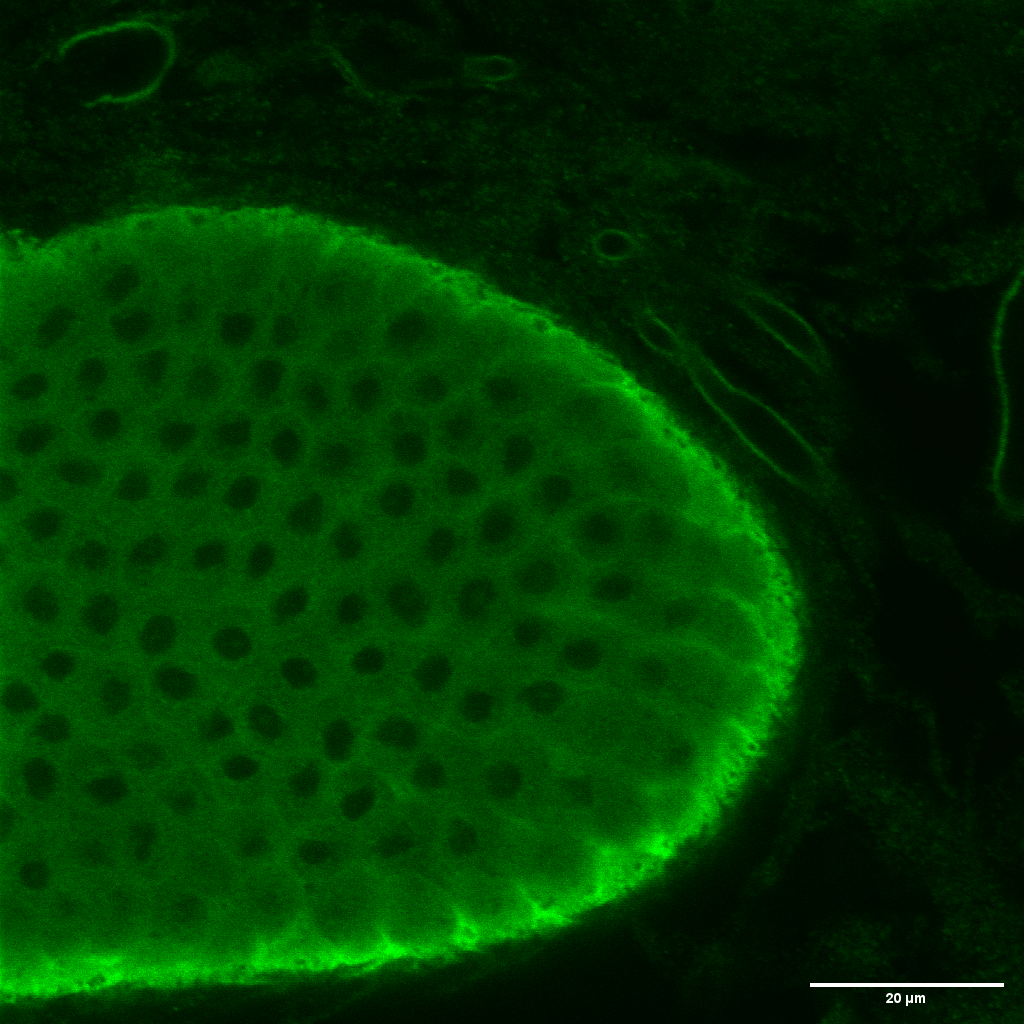

Supplement: Supplementary file 8 — Source data Fig. 6 [file 44319_2025_672_MOESM8_ESM.zip › Figure6/E/stage9-GFP.tif]

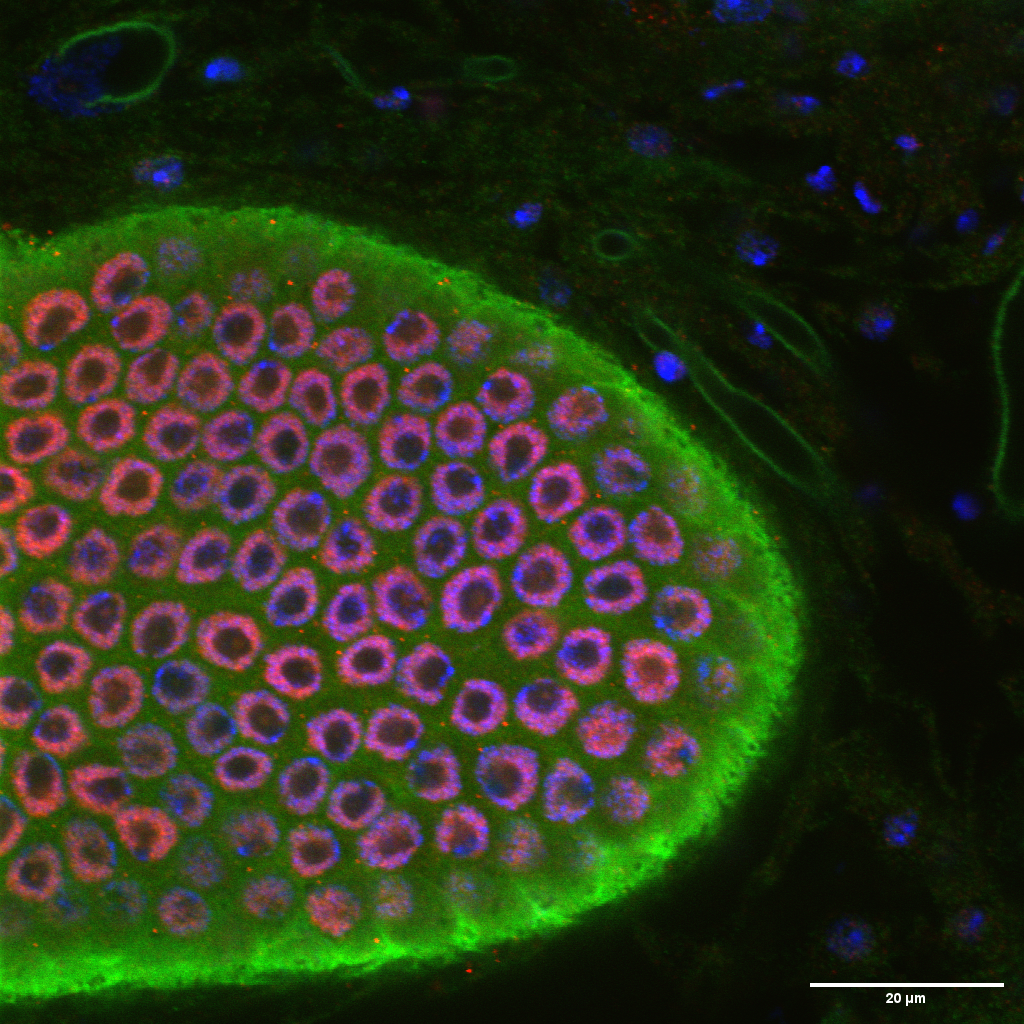

Supplement: Supplementary file 8 — Source data Fig. 6 [file 44319_2025_672_MOESM8_ESM.zip › Figure6/E/stage9-Merge.tif]

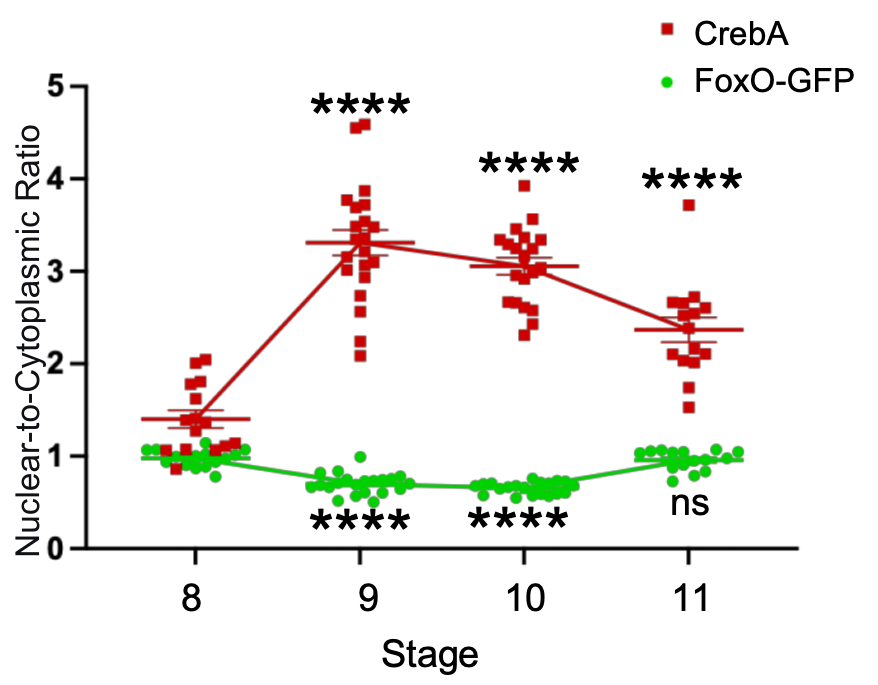

Supplement: Supplementary file 8 — Source data Fig. 6 [file 44319_2025_672_MOESM8_ESM.zip › Figure6/F/F.tif]

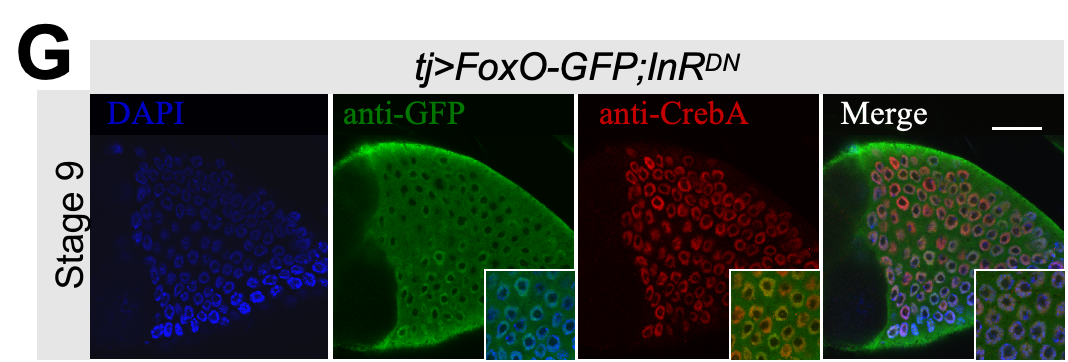

Supplement: Supplementary file 8 — Source data Fig. 6 [file 44319_2025_672_MOESM8_ESM.zip › Figure6/G/G.tif]

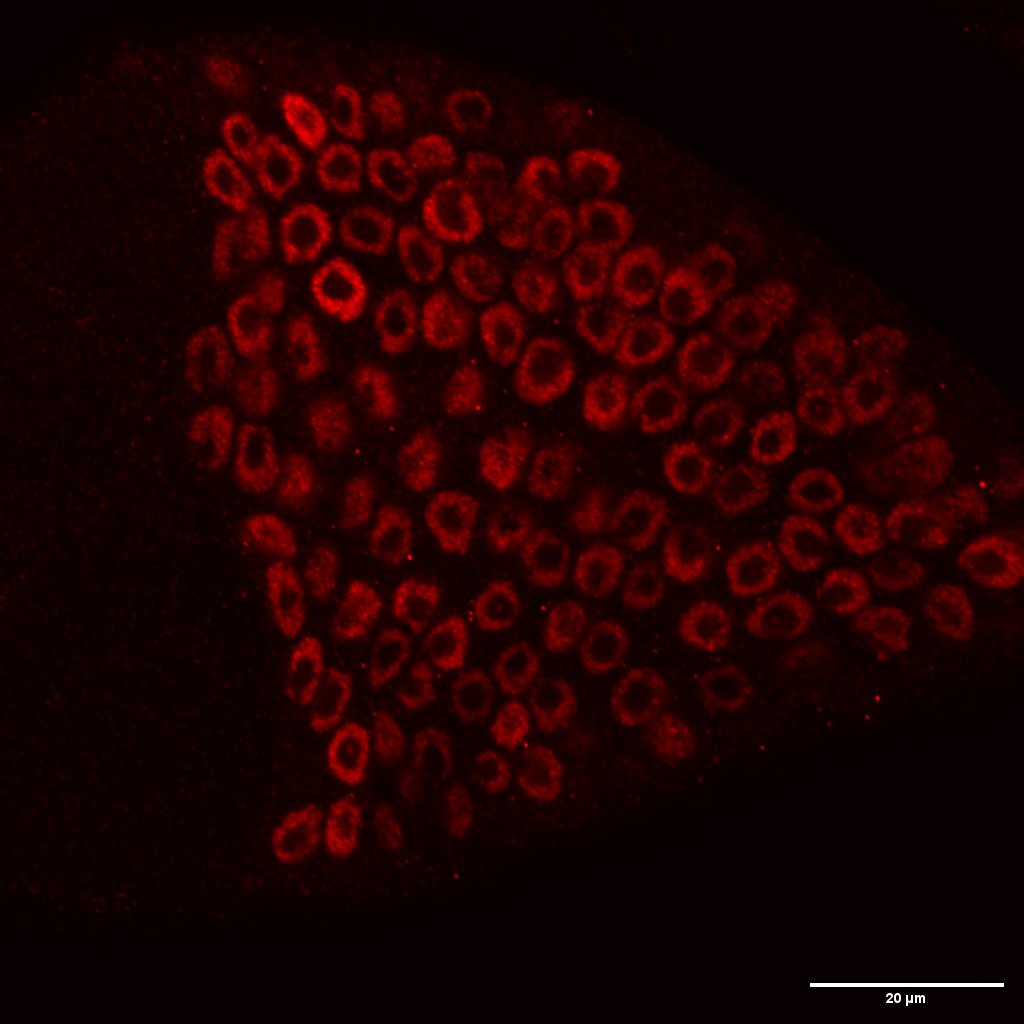

Supplement: Supplementary file 8 — Source data Fig. 6 [file 44319_2025_672_MOESM8_ESM.zip › Figure6/G/stage9-antiCrebA.tif]

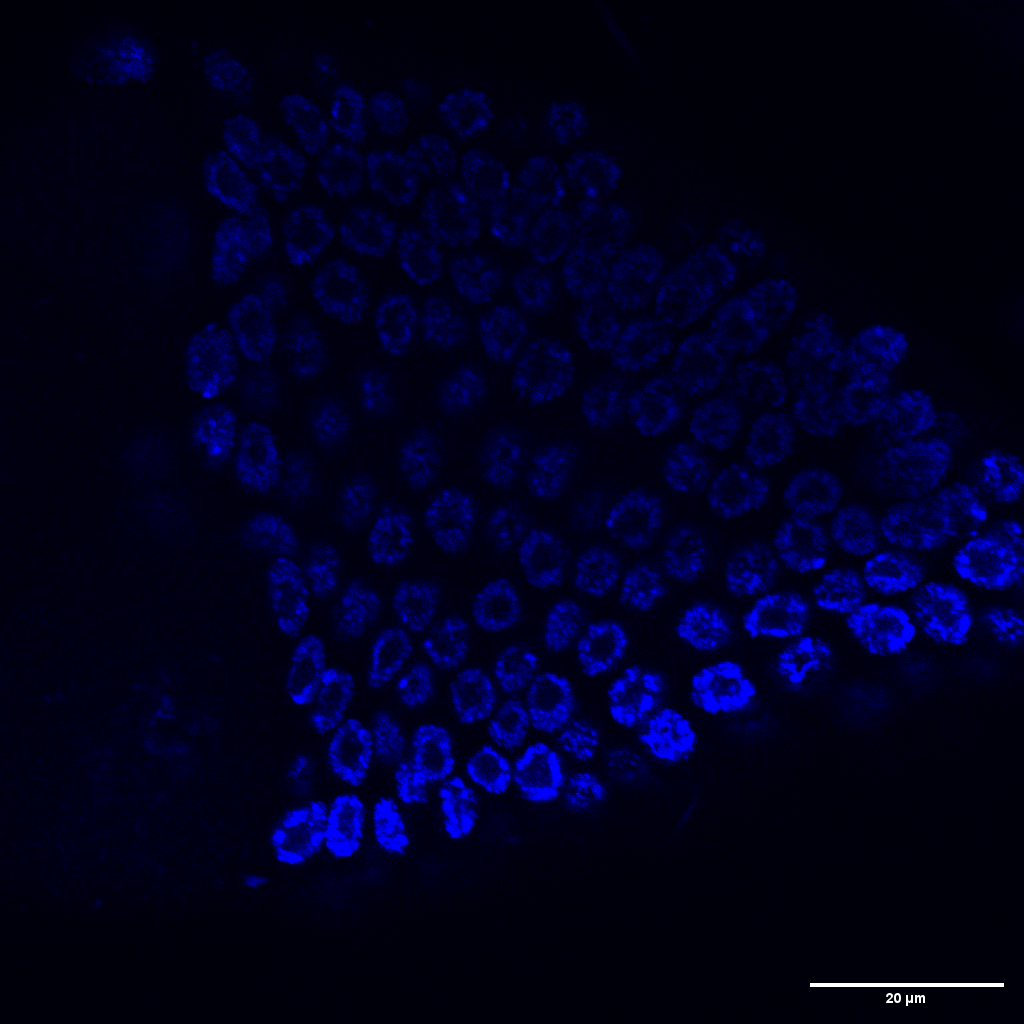

Supplement: Supplementary file 8 — Source data Fig. 6 [file 44319_2025_672_MOESM8_ESM.zip › Figure6/G/stage9-DAPI.tif]

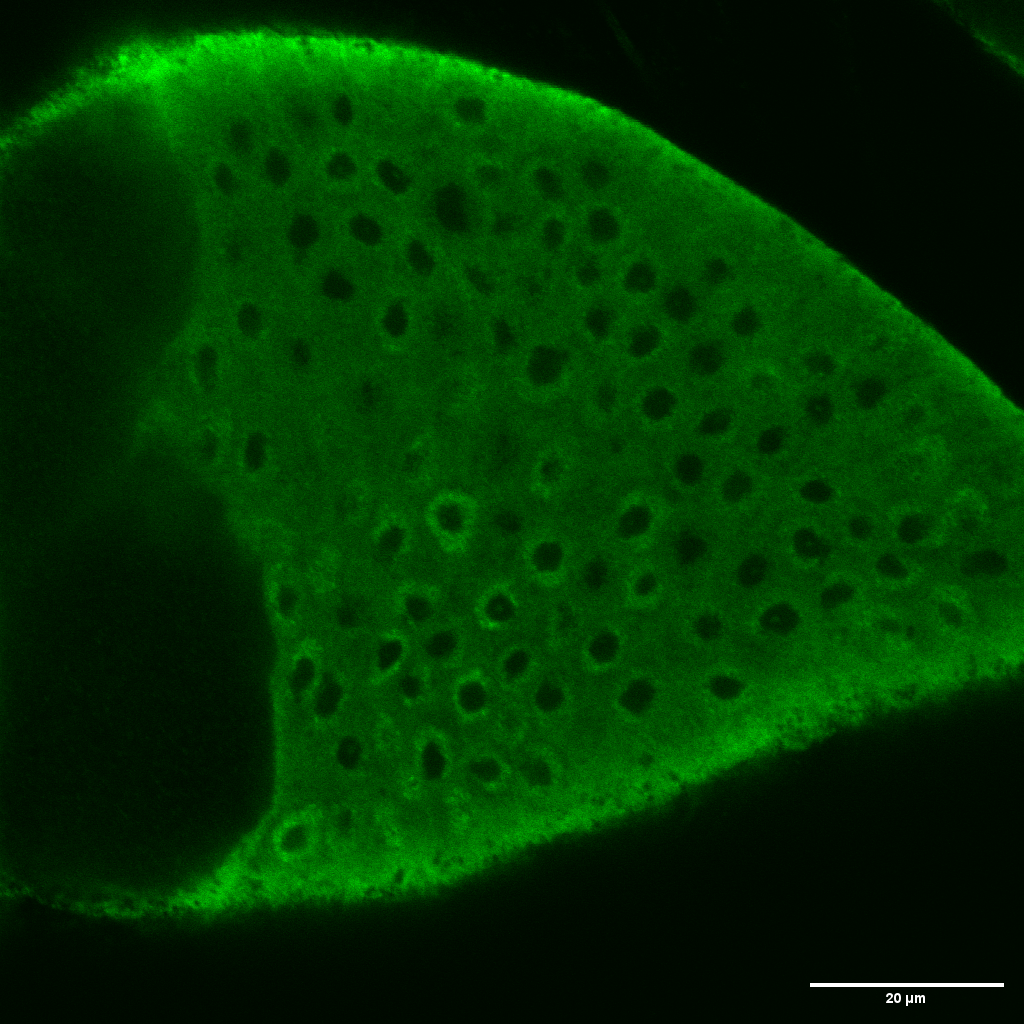

Supplement: Supplementary file 8 — Source data Fig. 6 [file 44319_2025_672_MOESM8_ESM.zip › Figure6/G/stage9-GFP.tif]

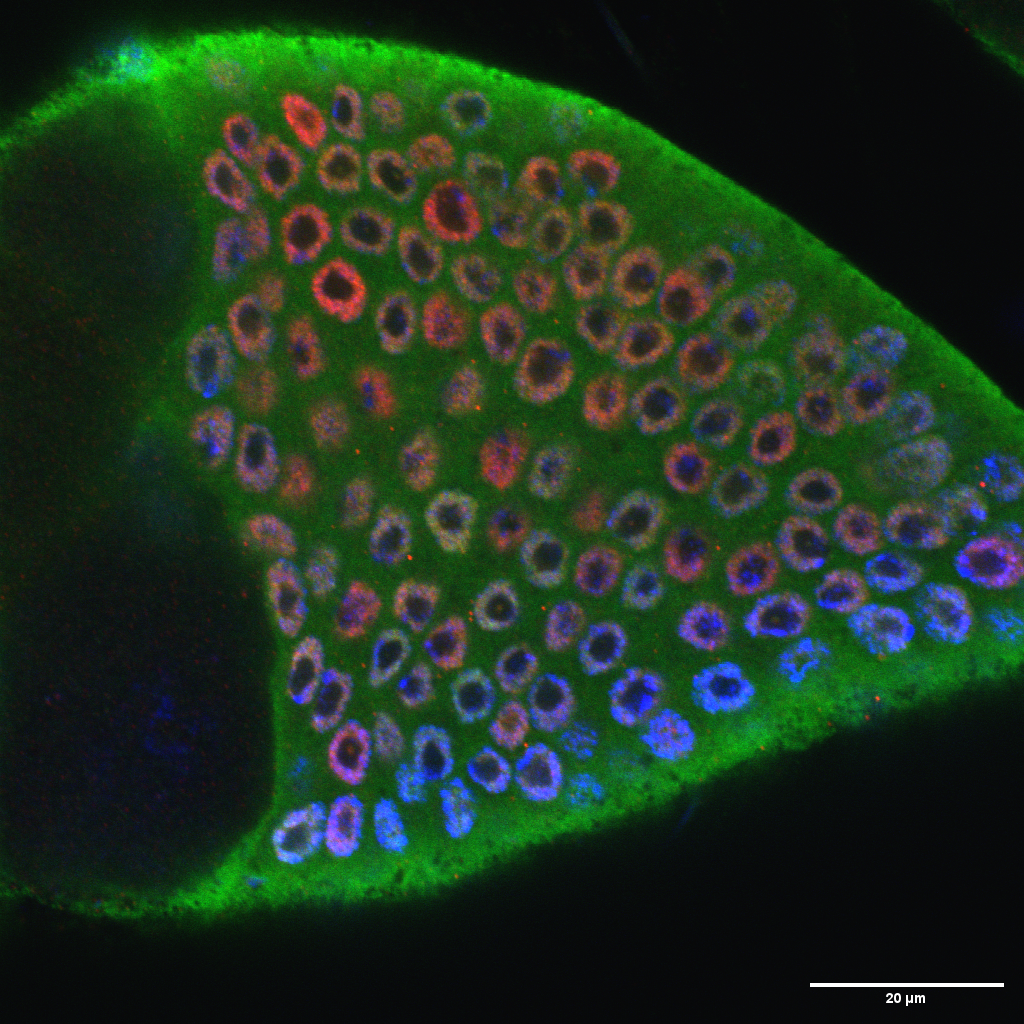

Supplement: Supplementary file 8 — Source data Fig. 6 [file 44319_2025_672_MOESM8_ESM.zip › Figure6/G/stage9-Merge.tif]

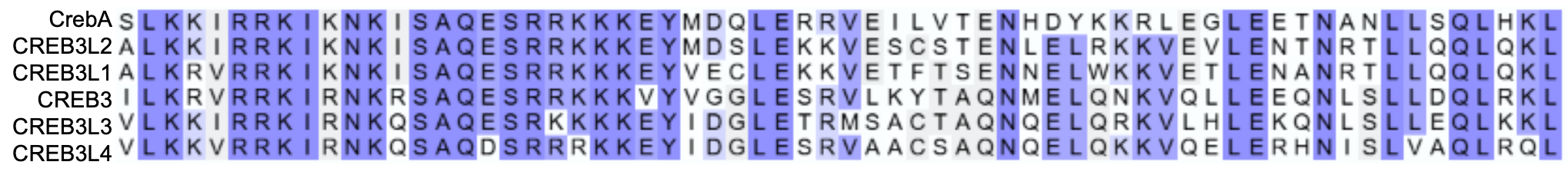

Supplement: Supplementary file 9 — Source data Fig. 7 [file 44319_2025_672_MOESM9_ESM.zip › Figure7/A/A.tif]

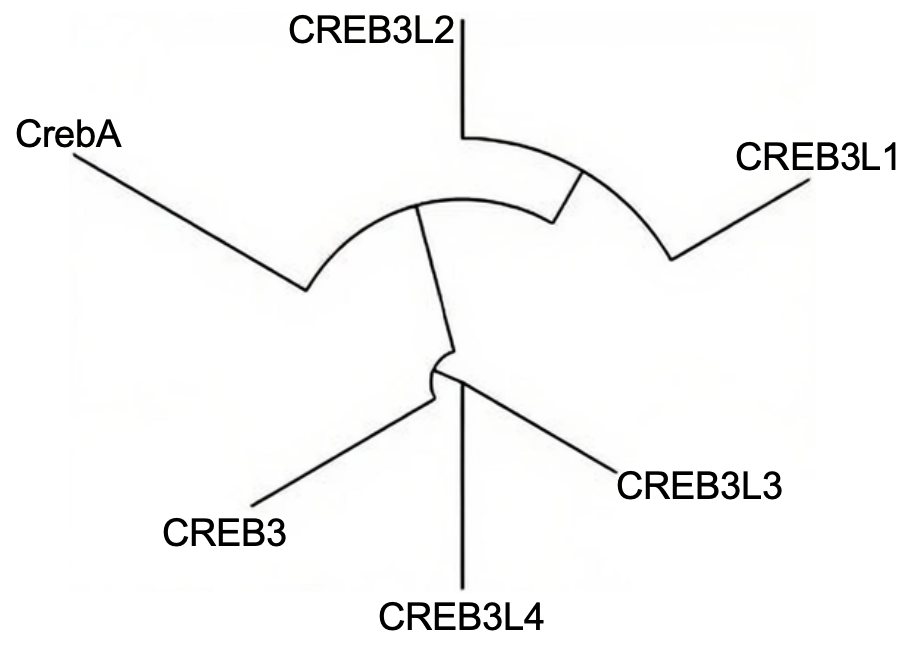

Supplement: Supplementary file 9 — Source data Fig. 7 [file 44319_2025_672_MOESM9_ESM.zip › Figure7/B/B.tif]

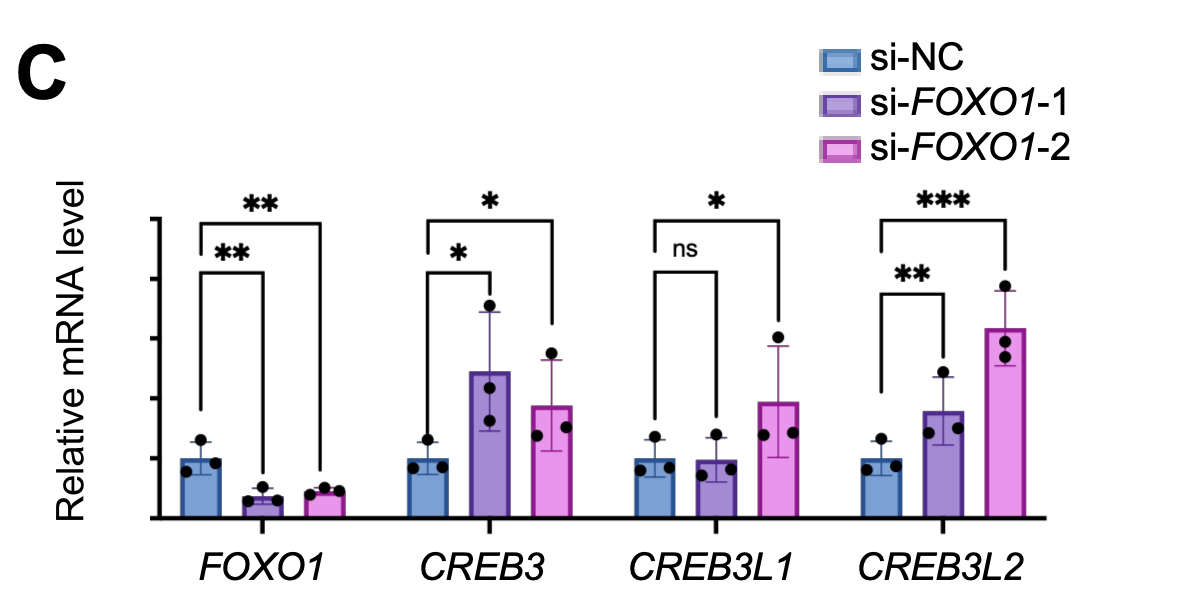

Supplement: Supplementary file 9 — Source data Fig. 7 [file 44319_2025_672_MOESM9_ESM.zip › Figure7/C/C.tif]

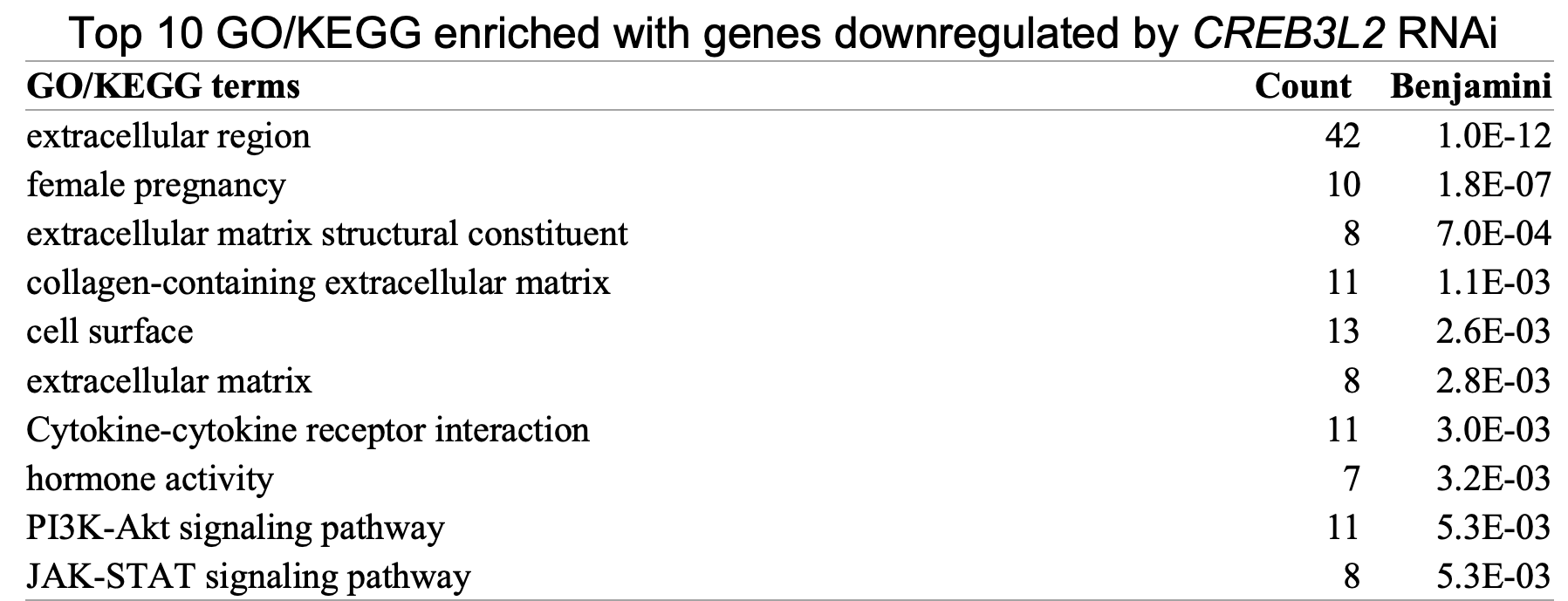

Supplement: Supplementary file 9 — Source data Fig. 7 [file 44319_2025_672_MOESM9_ESM.zip › Figure7/D/D.tif]

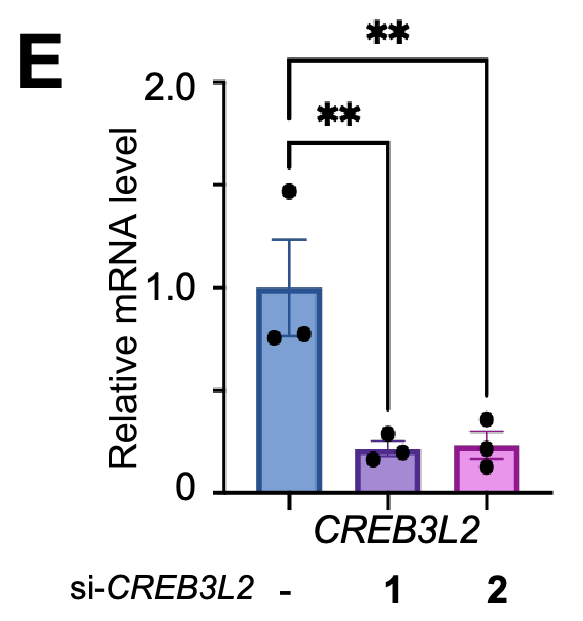

Supplement: Supplementary file 9 — Source data Fig. 7 [file 44319_2025_672_MOESM9_ESM.zip › Figure7/E/E.tif]

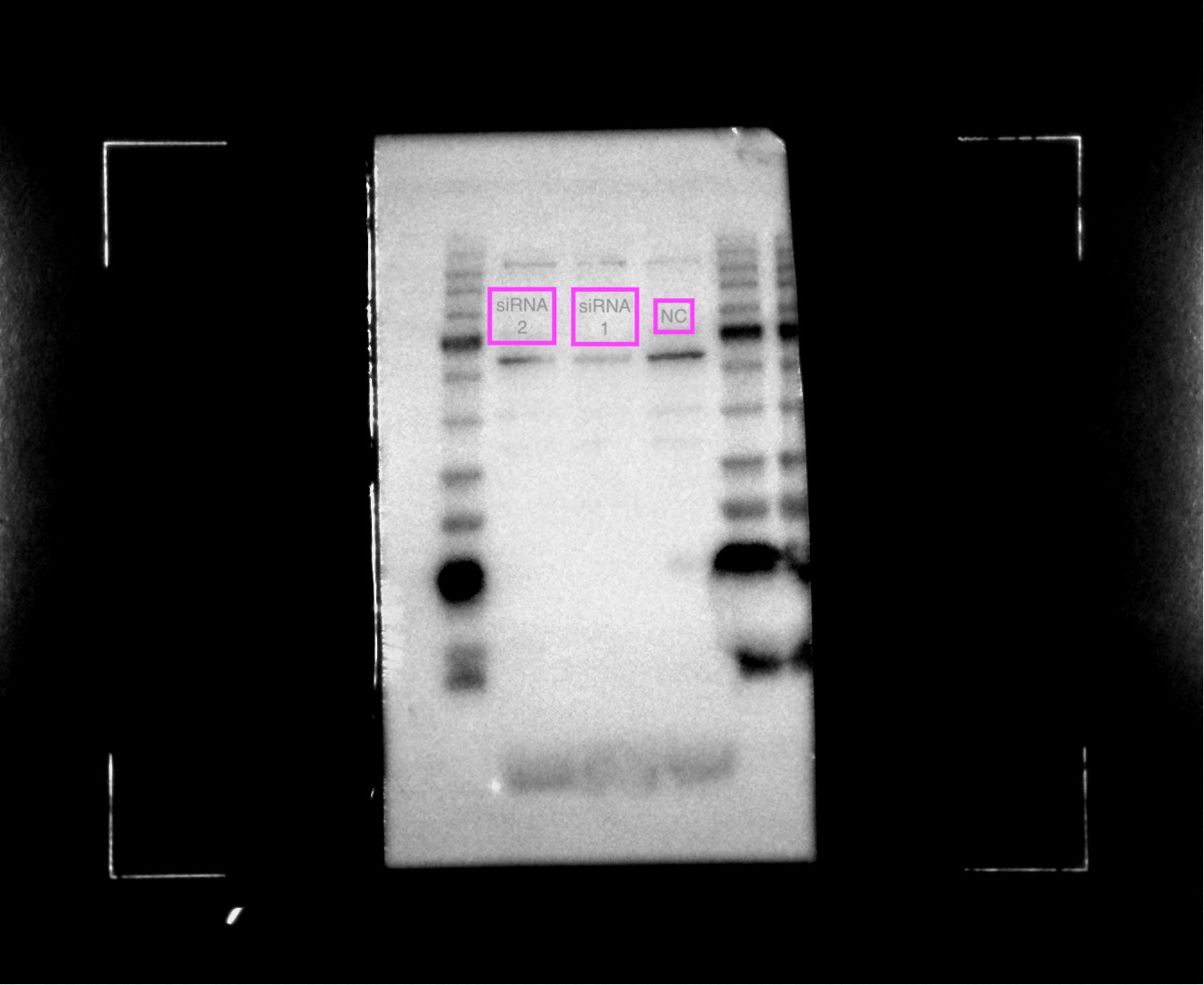

Supplement: Supplementary file 9 — Source data Fig. 7 [file 44319_2025_672_MOESM9_ESM.zip › Figure7/F/CPY19A1.jpg]
