# Supplementary figures and images for: An insulin receptor activity surge in follicle cells drives vitellogenesis by upregulating CrebA (part 2 of 2)
Source: EMBO Rep. 2026 Jan 3;27(3):748–73. doi: 10.1038/s44319-025-00672-6 (PMC12894986; doi:10.1038/s44319-025-00672-6)

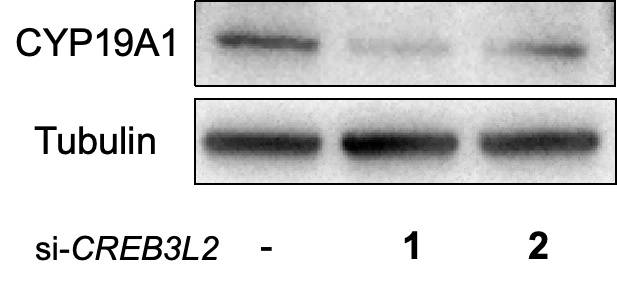

Supplement: Supplementary file 9 — Source data Fig. 7 [file 44319_2025_672_MOESM9_ESM.zip › Figure7/F/F.tif]

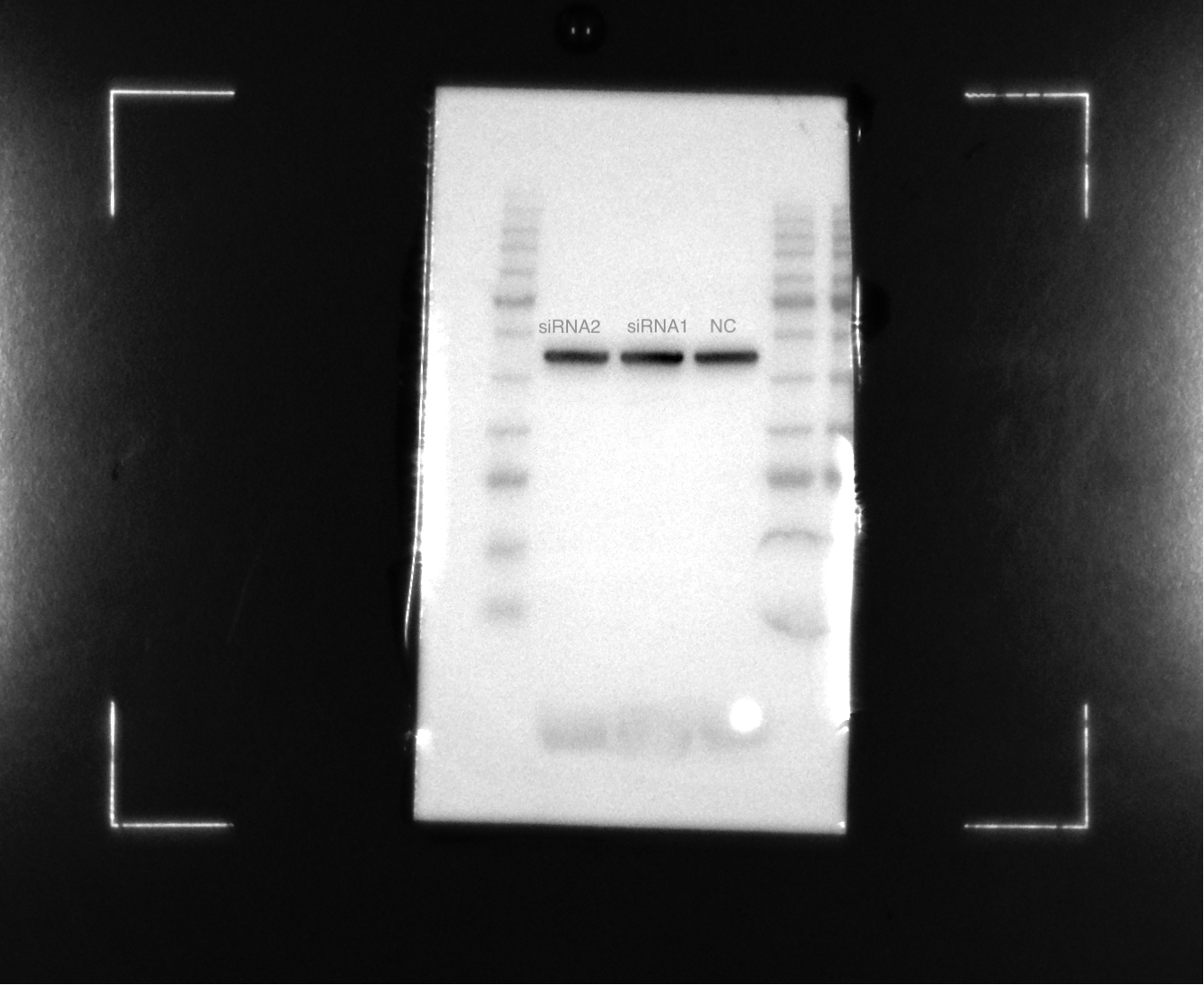

Supplement: Supplementary file 9 — Source data Fig. 7 [file 44319_2025_672_MOESM9_ESM.zip › Figure7/F/tubulin.tif]

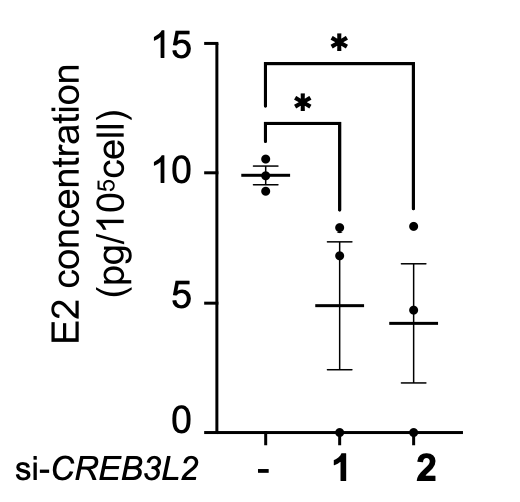

Supplement: Supplementary file 9 — Source data Fig. 7 [file 44319_2025_672_MOESM9_ESM.zip › Figure7/G/G.tif]

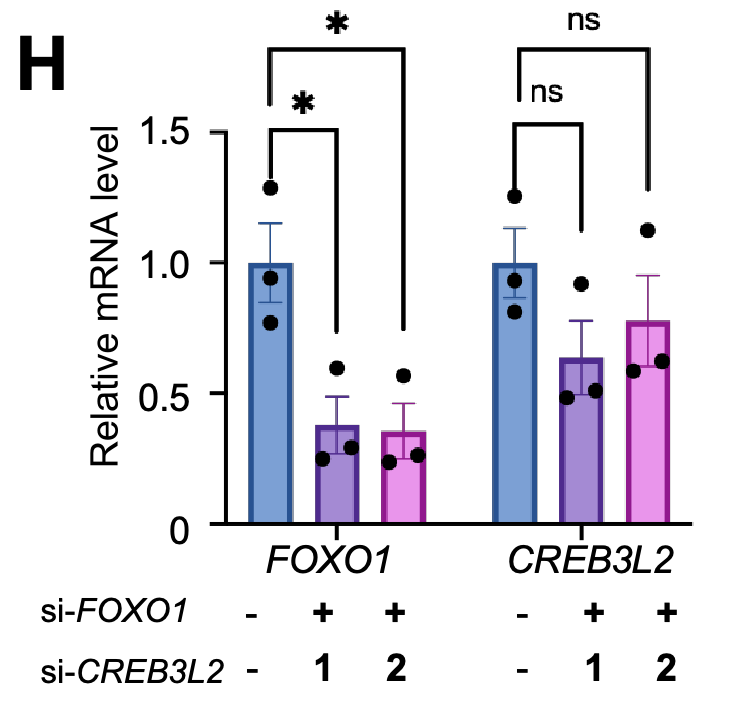

Supplement: Supplementary file 9 — Source data Fig. 7 [file 44319_2025_672_MOESM9_ESM.zip › Figure7/H/H.tif]

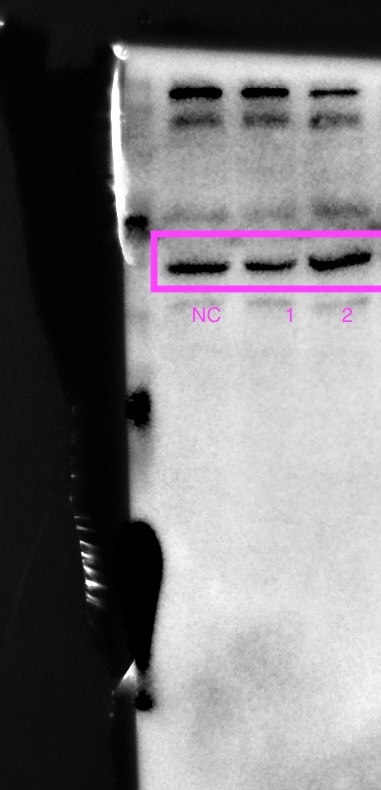

Supplement: Supplementary file 9 — Source data Fig. 7 [file 44319_2025_672_MOESM9_ESM.zip › Figure7/I/CYP19A1.jpg]

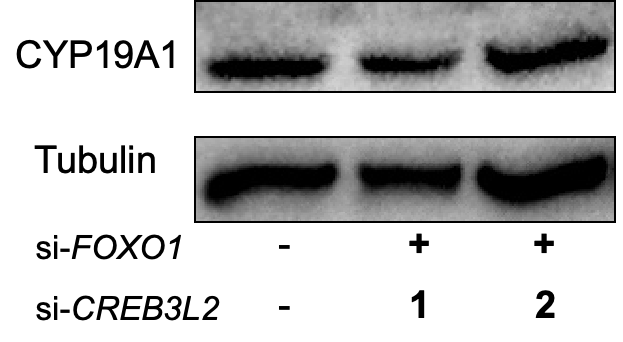

Supplement: Supplementary file 9 — Source data Fig. 7 [file 44319_2025_672_MOESM9_ESM.zip › Figure7/I/I.tif]

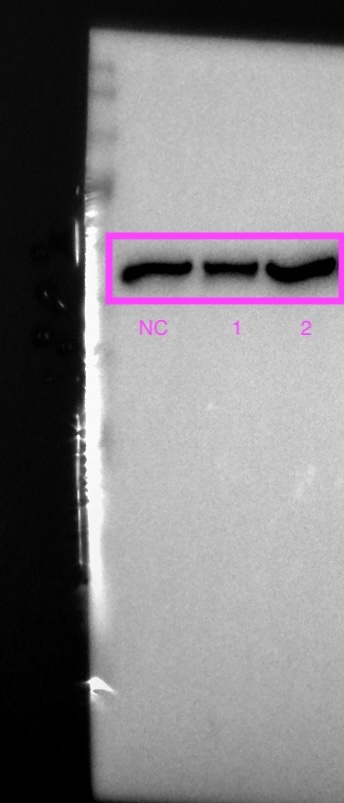

Supplement: Supplementary file 9 — Source data Fig. 7 [file 44319_2025_672_MOESM9_ESM.zip › Figure7/I/tubulin.jpg]

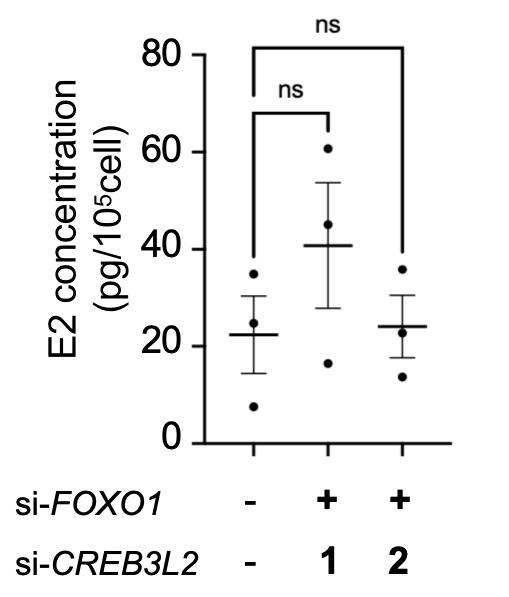

Supplement: Supplementary file 9 — Source data Fig. 7 [file 44319_2025_672_MOESM9_ESM.zip › Figure7/J/J.tif]

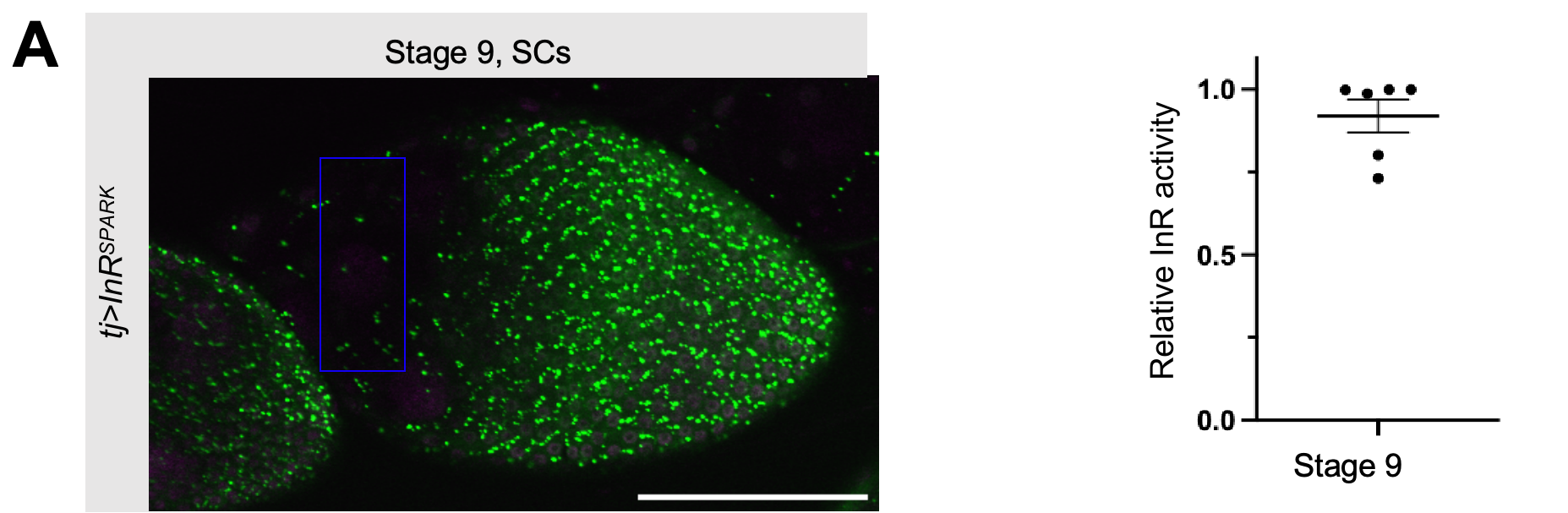

Supplement: Supplementary file 10 — Figure EV1 Source Data [file 44319_2025_672_MOESM10_ESM.zip › EV1/A/A.tif]

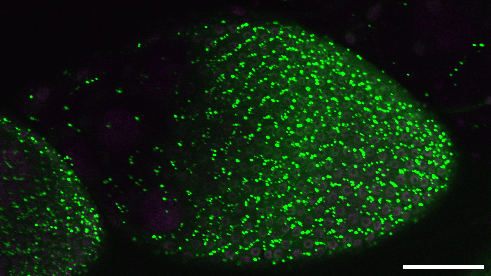

Supplement: Supplementary file 10 — Figure EV1 Source Data [file 44319_2025_672_MOESM10_ESM.zip › EV1/A/stage9-surface.tif]

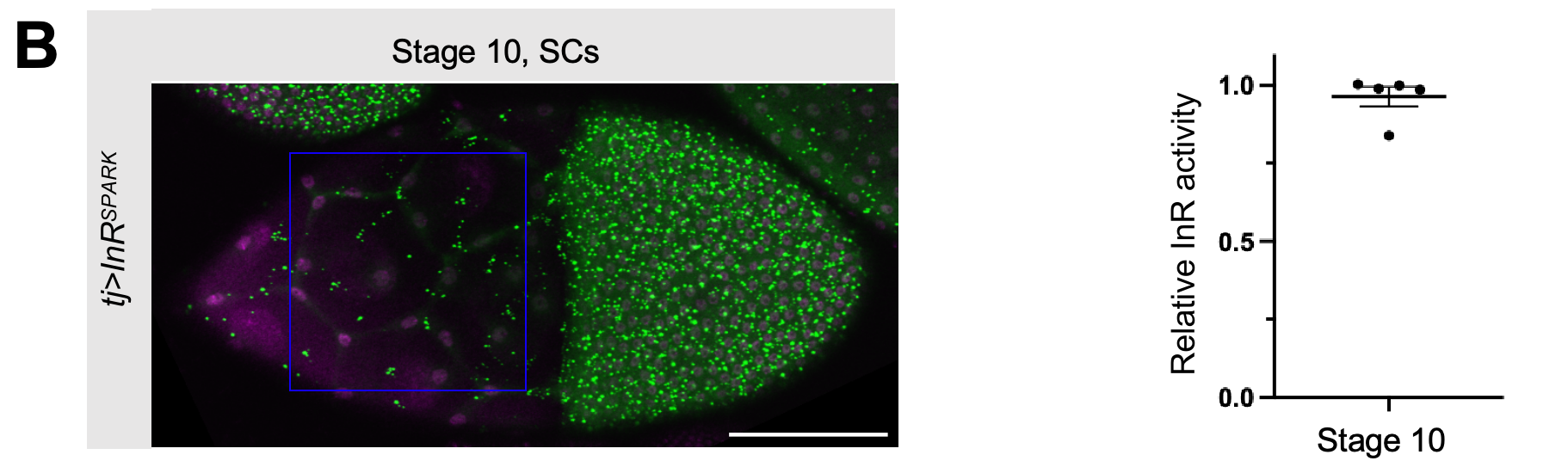

Supplement: Supplementary file 10 — Figure EV1 Source Data [file 44319_2025_672_MOESM10_ESM.zip › EV1/B/B.tif]

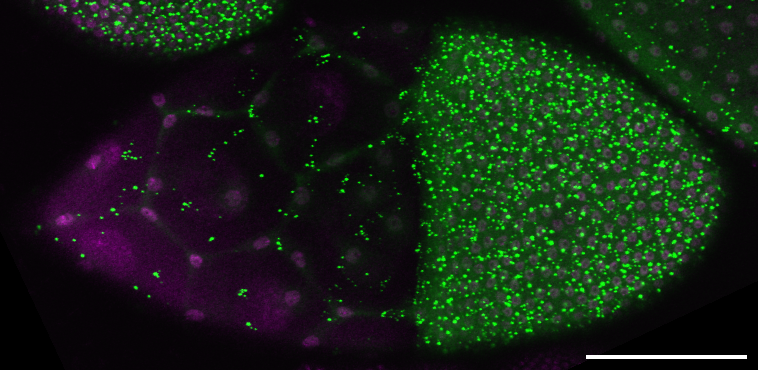

Supplement: Supplementary file 10 — Figure EV1 Source Data [file 44319_2025_672_MOESM10_ESM.zip › EV1/B/stage1-11-20x-1-500-1-17-30-wxy-3_Z003-2.tif]

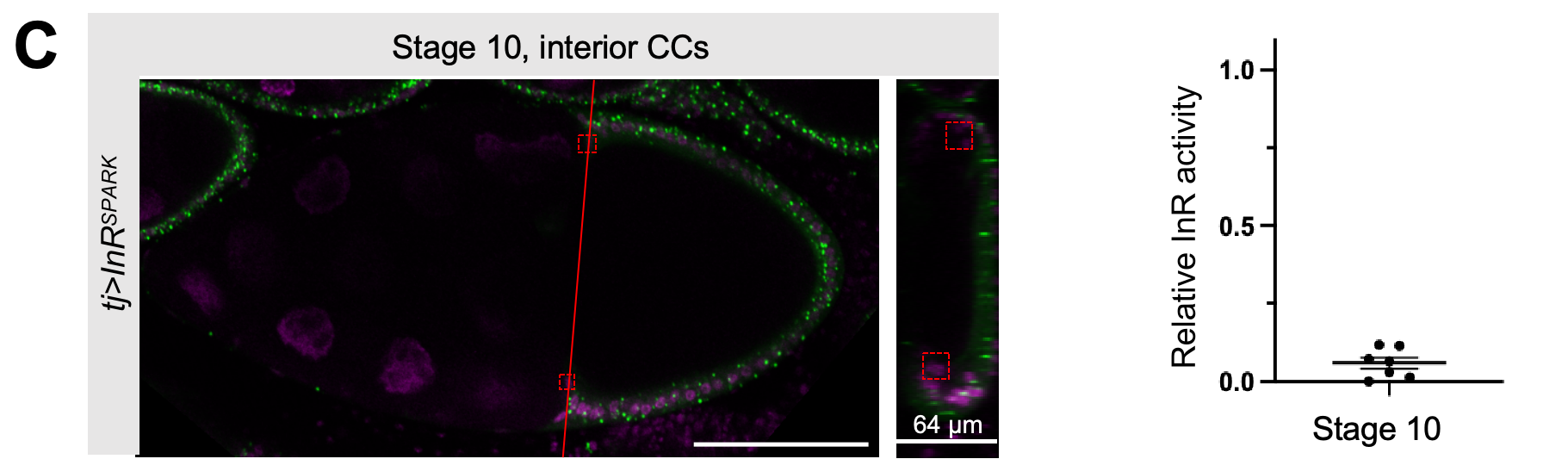

Supplement: Supplementary file 10 — Figure EV1 Source Data [file 44319_2025_672_MOESM10_ESM.zip › EV1/C/C.tif]

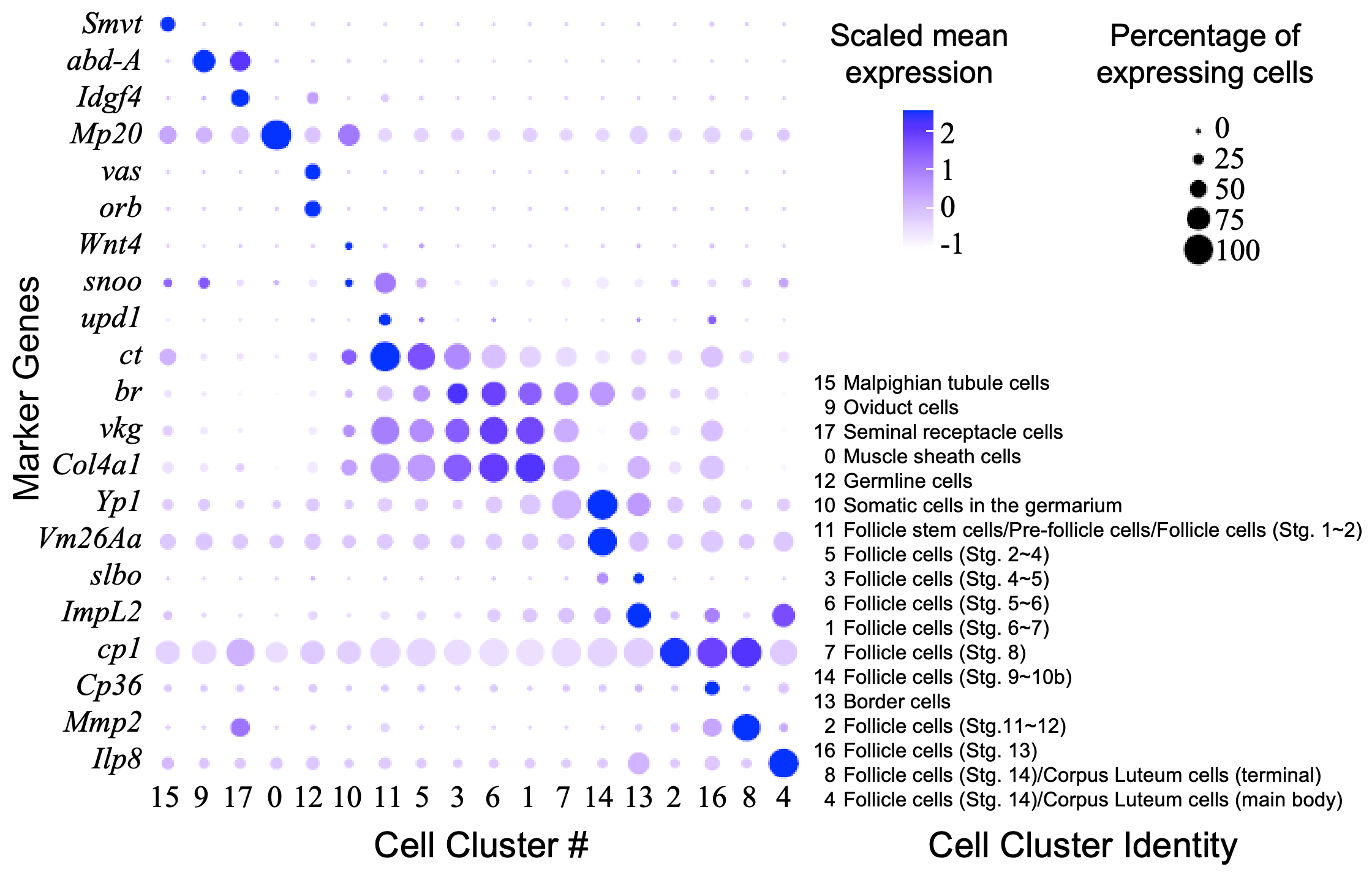

Supplement: Supplementary file 11 — Figure EV2 Source Data [file 44319_2025_672_MOESM11_ESM.zip › EV2/EV2.tif]

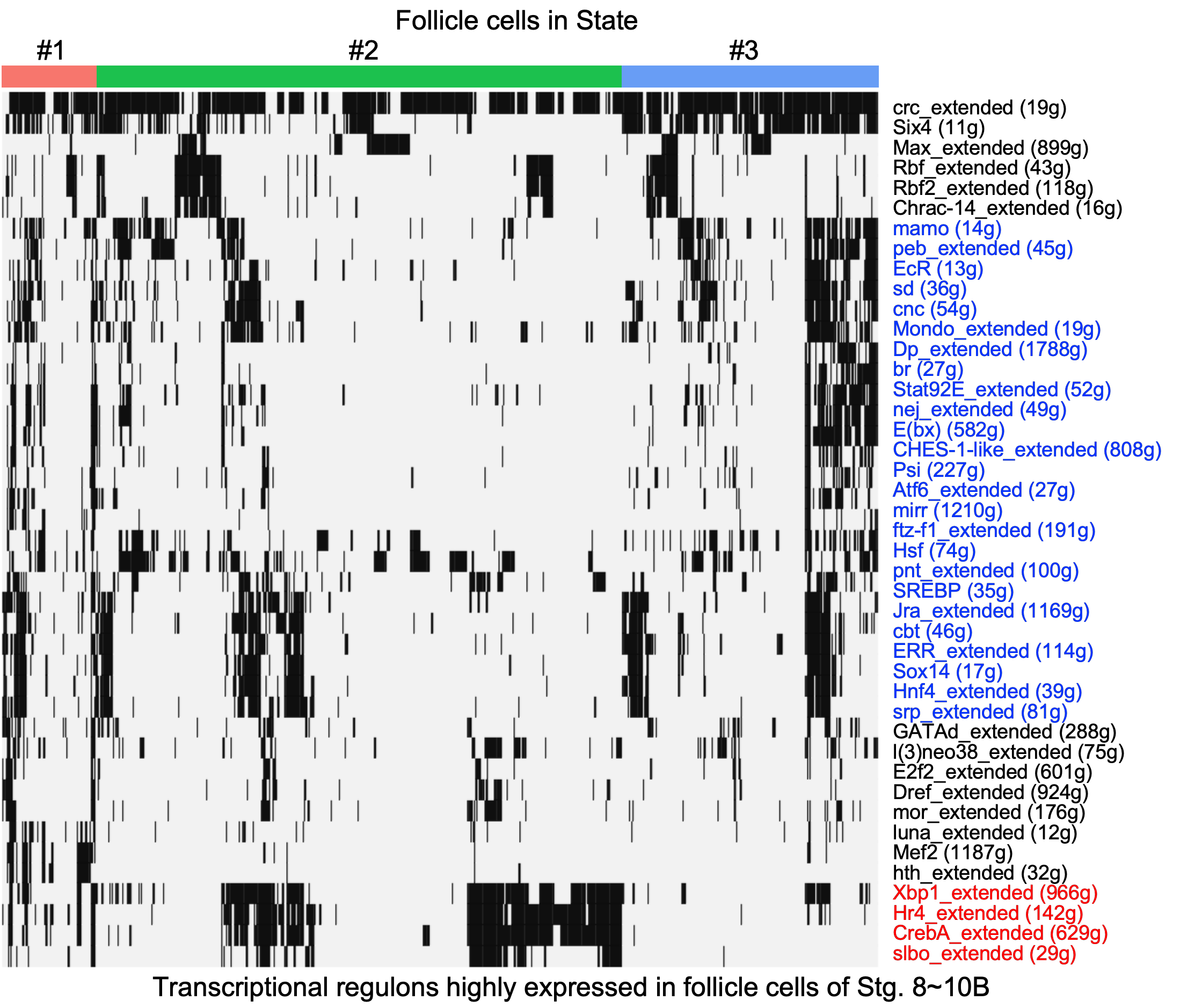

Supplement: Supplementary file 12 — Figure EV3 Source Data [file 44319_2025_672_MOESM12_ESM.zip › EV3/EV3.tif]

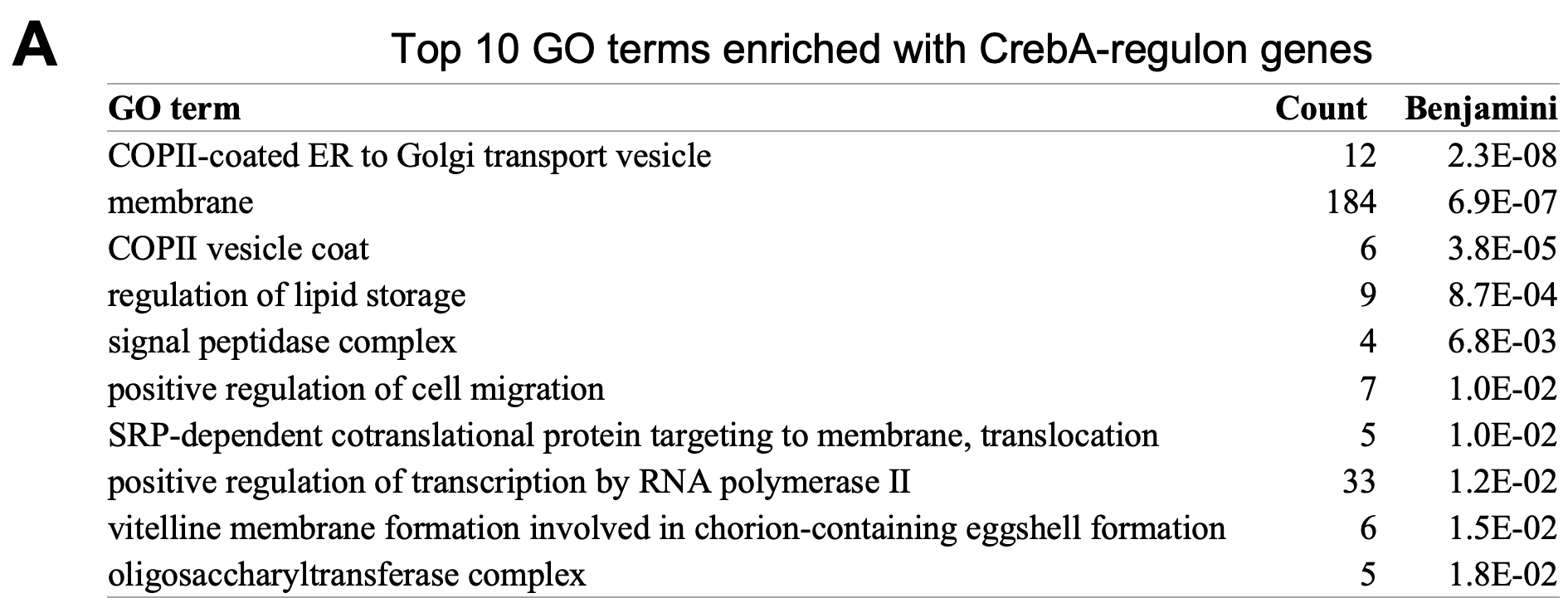

Supplement: Supplementary file 13 — Figure EV4 Source Data [file 44319_2025_672_MOESM13_ESM.zip › EV4/A.tif]

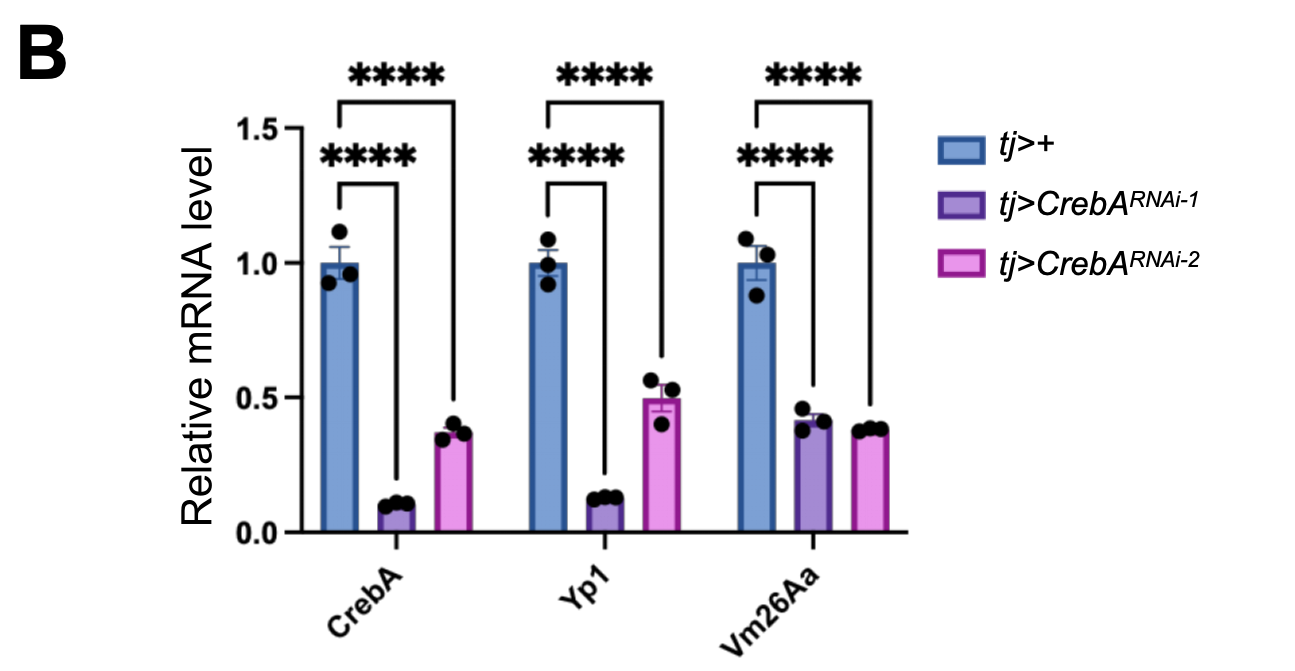

Supplement: Supplementary file 13 — Figure EV4 Source Data [file 44319_2025_672_MOESM13_ESM.zip › EV4/B.tif]

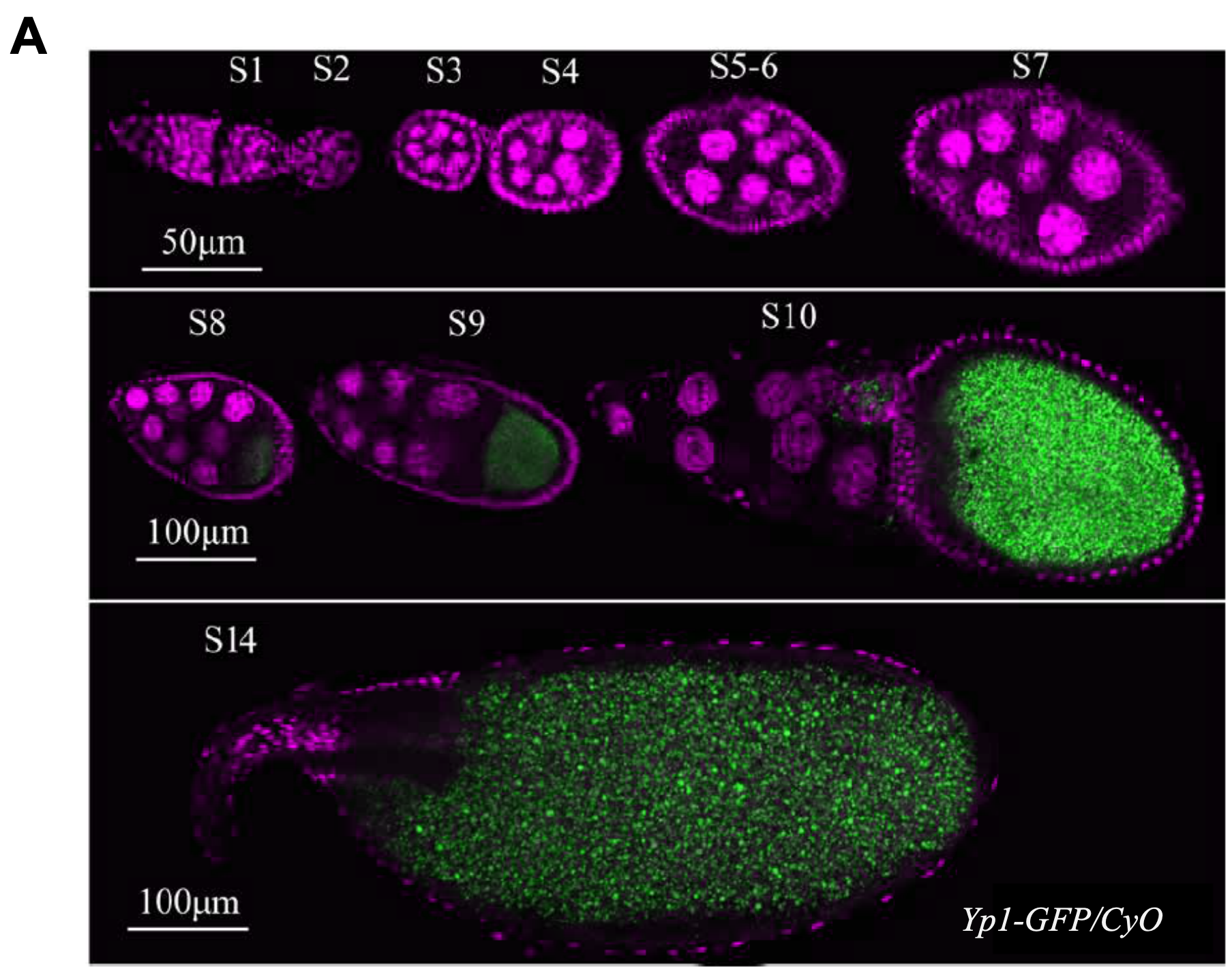

Supplement: Supplementary file 14 — Figure EV5 Source Data [file 44319_2025_672_MOESM14_ESM.zip › EV5/A/Atif.tif]

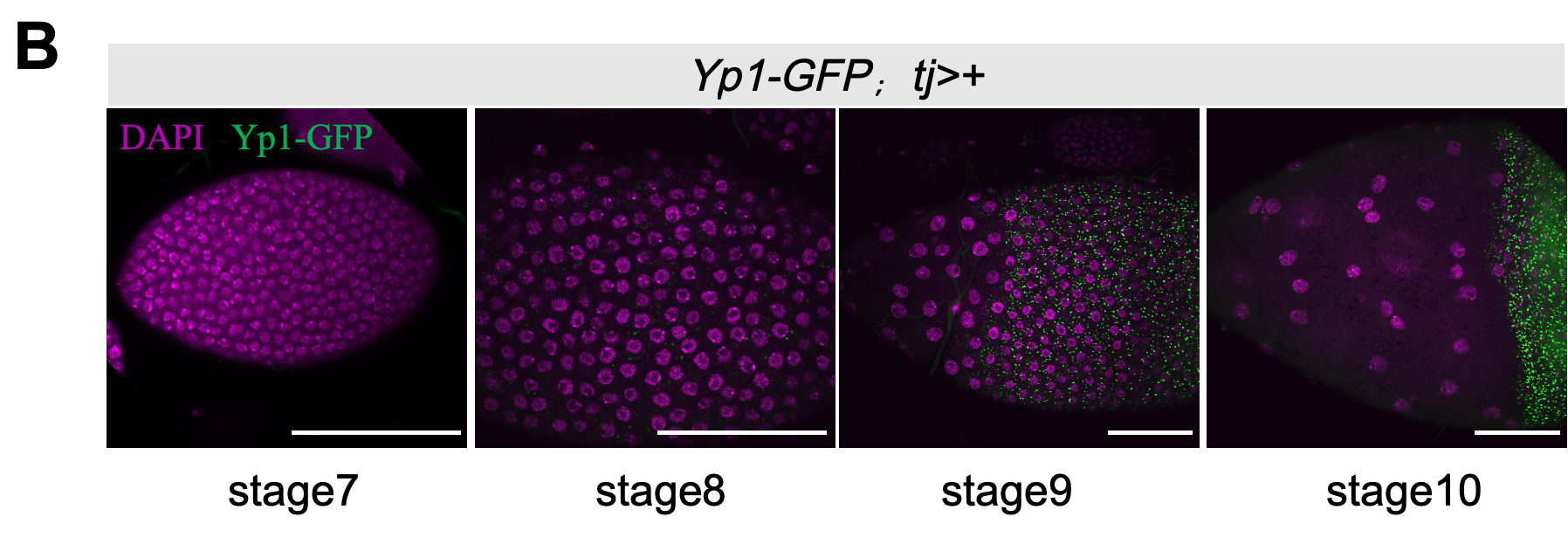

Supplement: Supplementary file 14 — Figure EV5 Source Data [file 44319_2025_672_MOESM14_ESM.zip › EV5/B/B.tif]

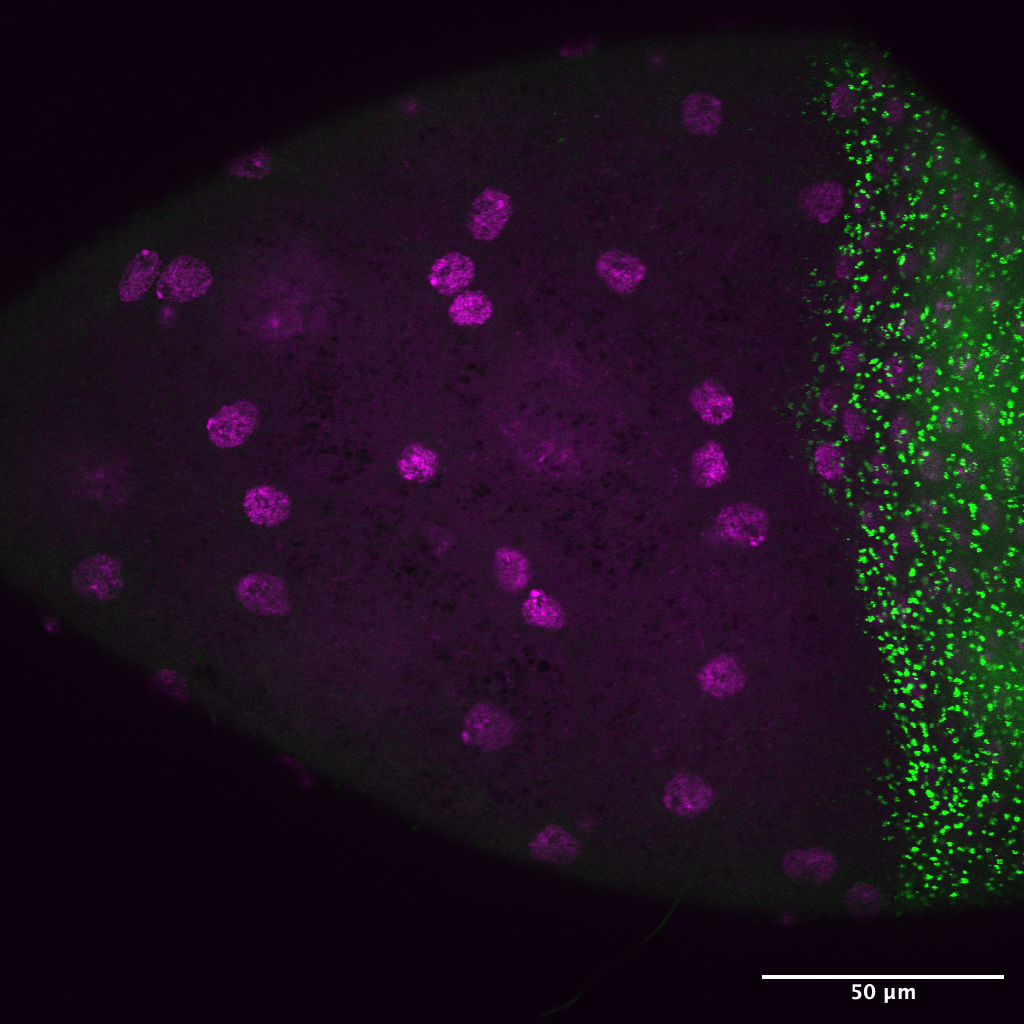

Supplement: Supplementary file 14 — Figure EV5 Source Data [file 44319_2025_672_MOESM14_ESM.zip › EV5/B/stage10-sc_-1.tif]

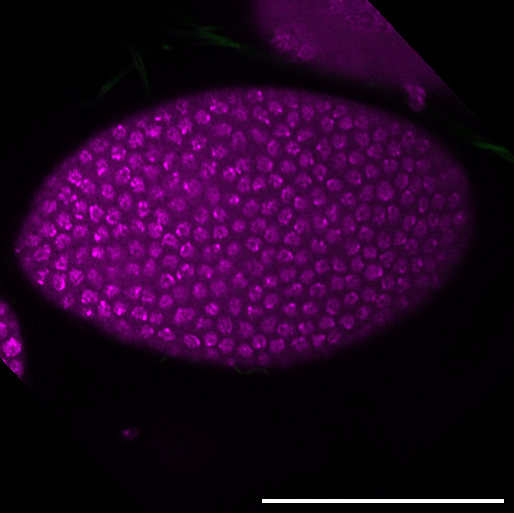

Supplement: Supplementary file 14 — Figure EV5 Source Data [file 44319_2025_672_MOESM14_ESM.zip › EV5/B/stage1-7-sc-1_-2.tif]

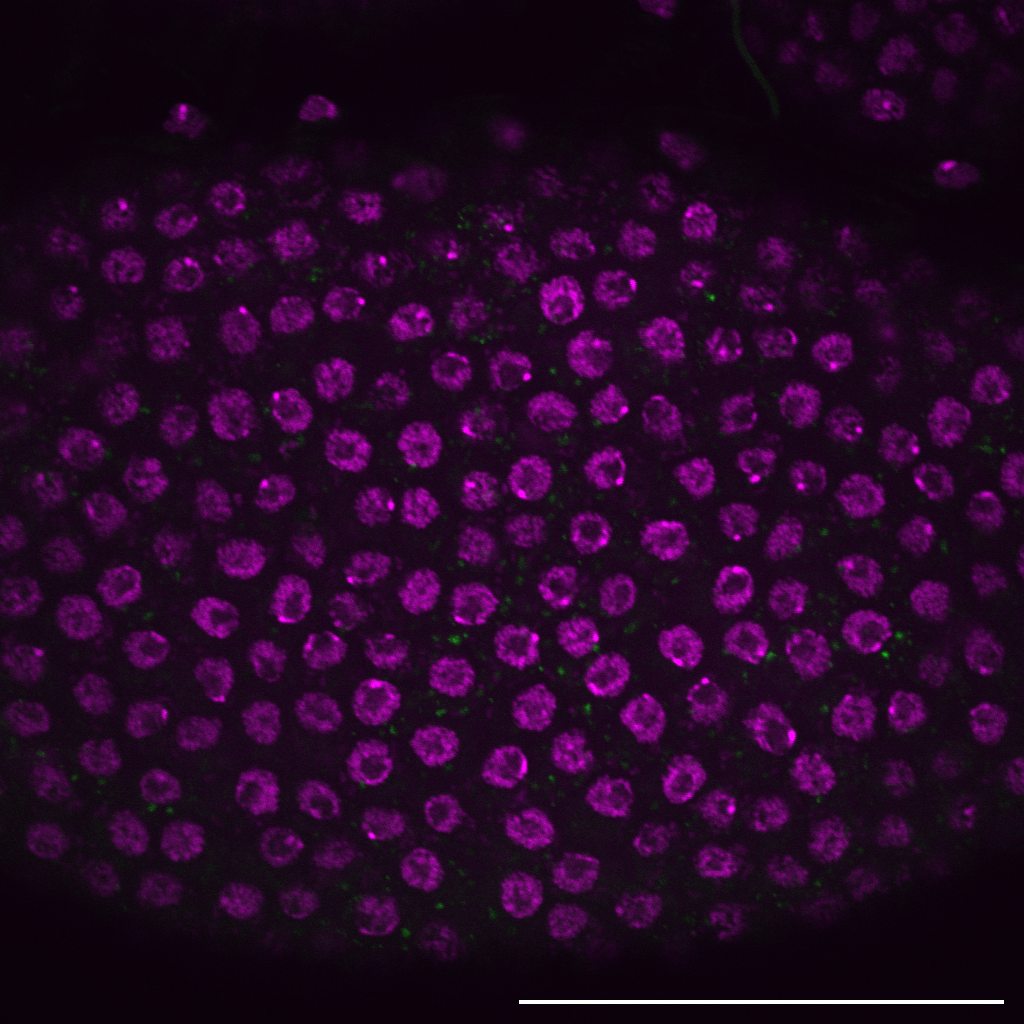

Supplement: Supplementary file 14 — Figure EV5 Source Data [file 44319_2025_672_MOESM14_ESM.zip › EV5/B/stage8-60x-2-1.tif]

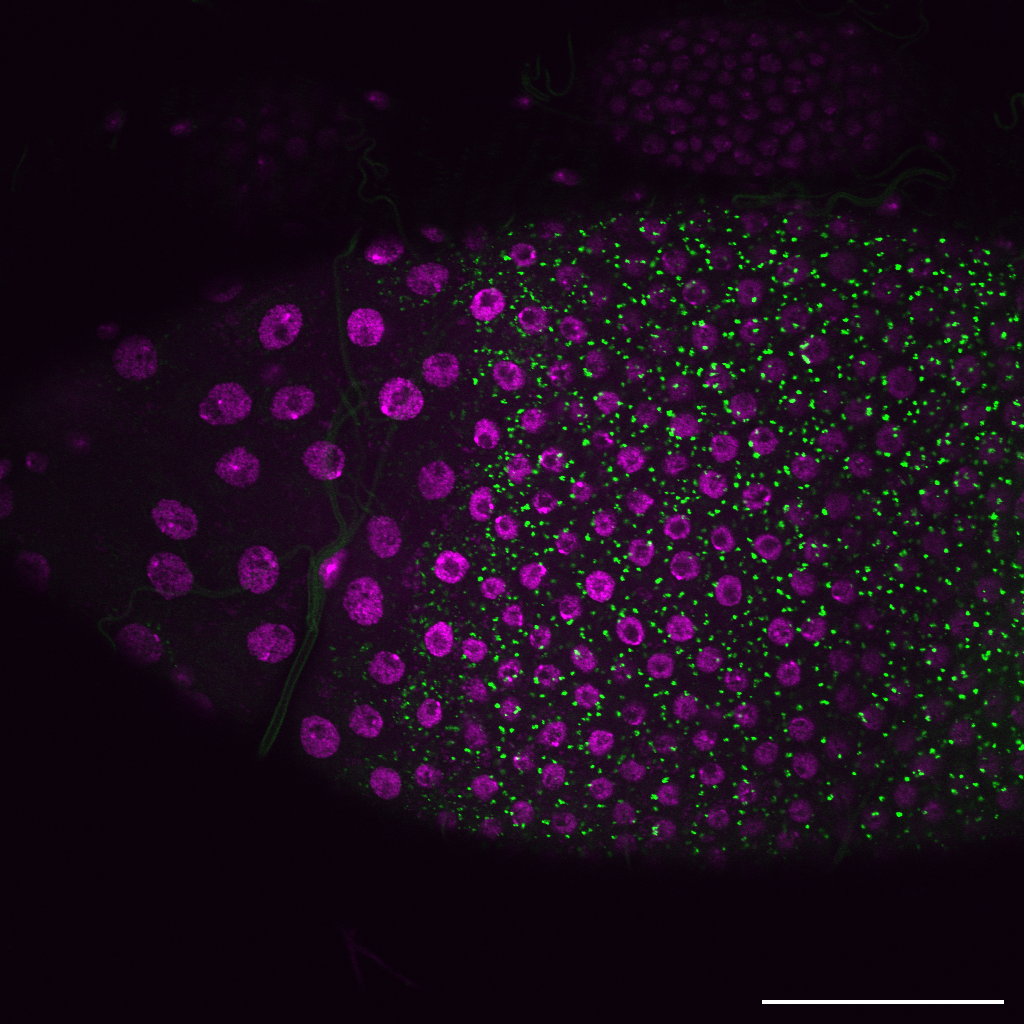

Supplement: Supplementary file 14 — Figure EV5 Source Data [file 44319_2025_672_MOESM14_ESM.zip › EV5/B/stage9-60x-1-sc-1.tif]

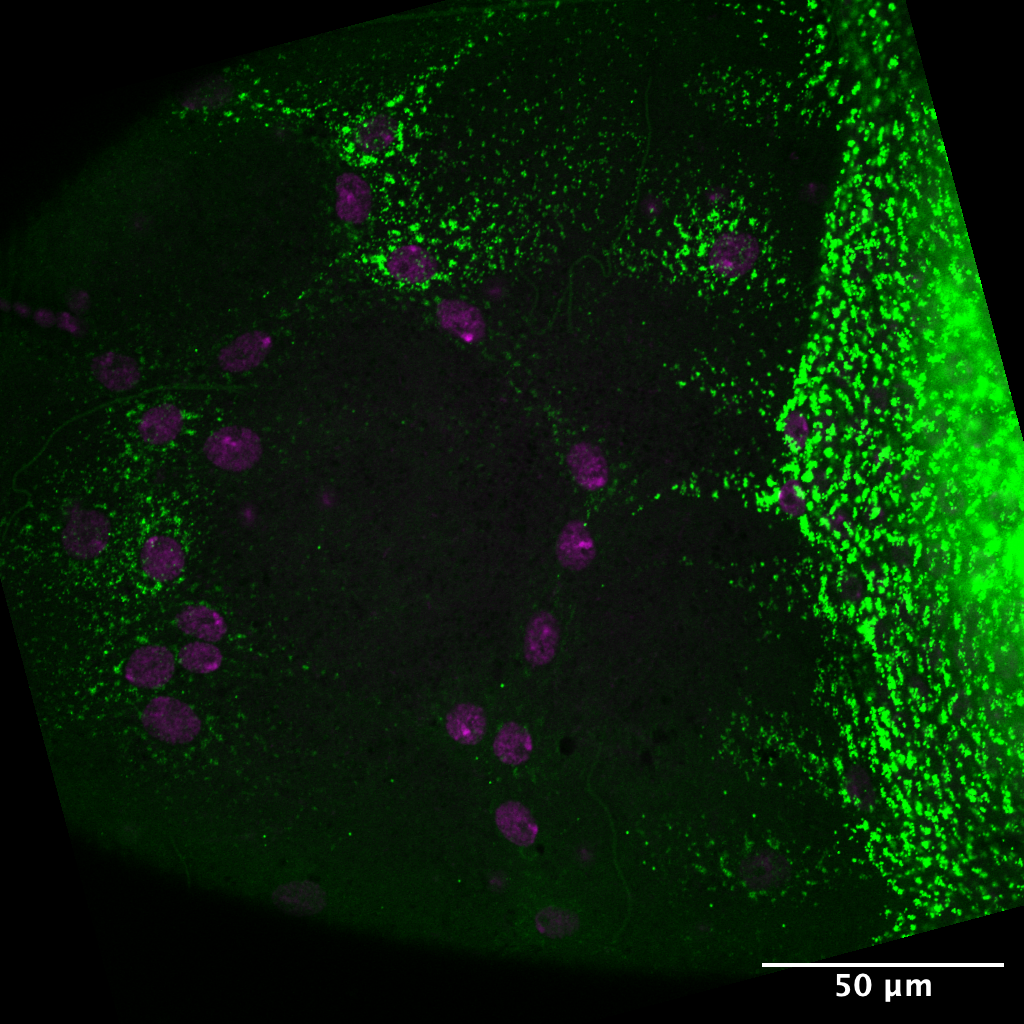

Supplement: Supplementary file 14 — Figure EV5 Source Data [file 44319_2025_672_MOESM14_ESM.zip › EV5/C/79021-stage10-sc-1.tif]

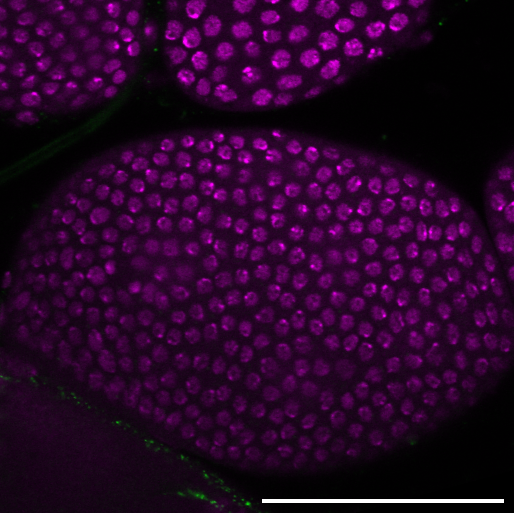

Supplement: Supplementary file 14 — Figure EV5 Source Data [file 44319_2025_672_MOESM14_ESM.zip › EV5/C/79021-stage7-sc-2_-2.tif]

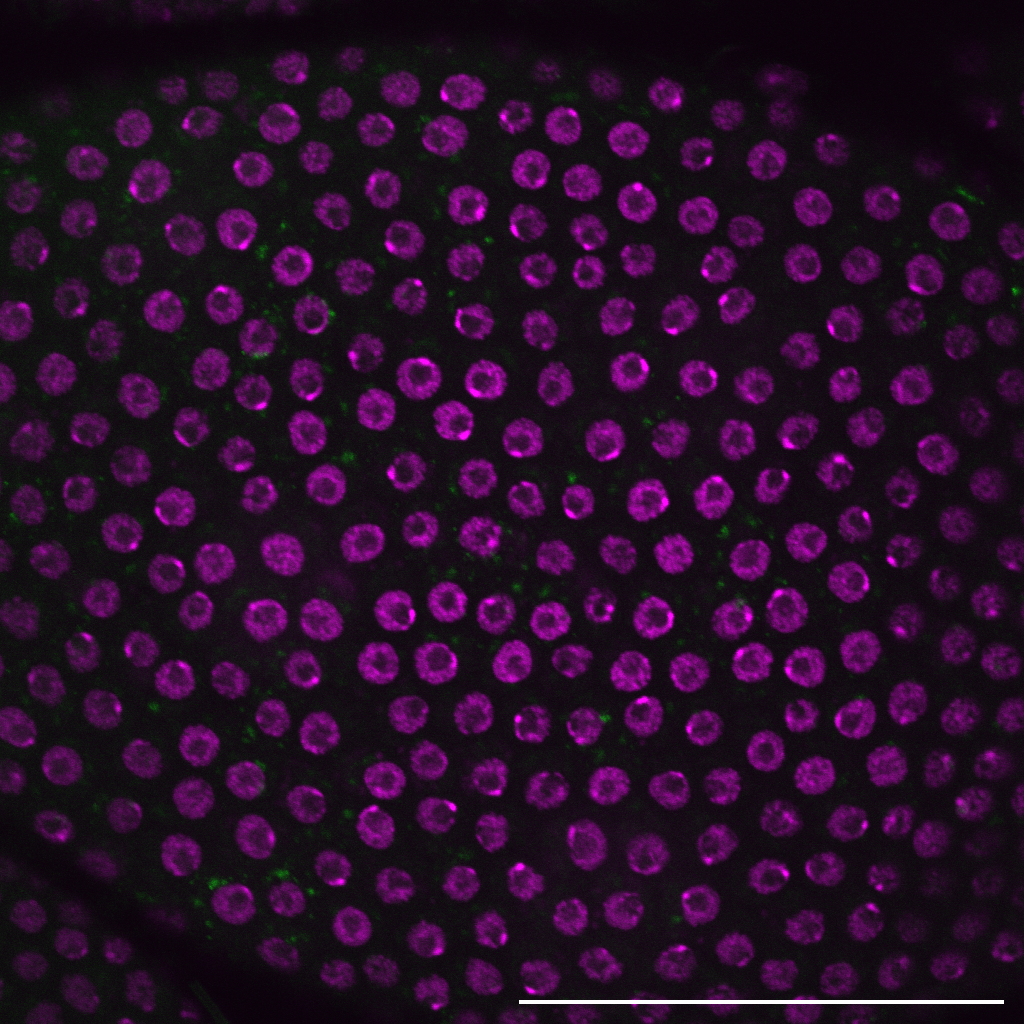

Supplement: Supplementary file 14 — Figure EV5 Source Data [file 44319_2025_672_MOESM14_ESM.zip › EV5/C/79021-stage8-60x-2-1.tif]

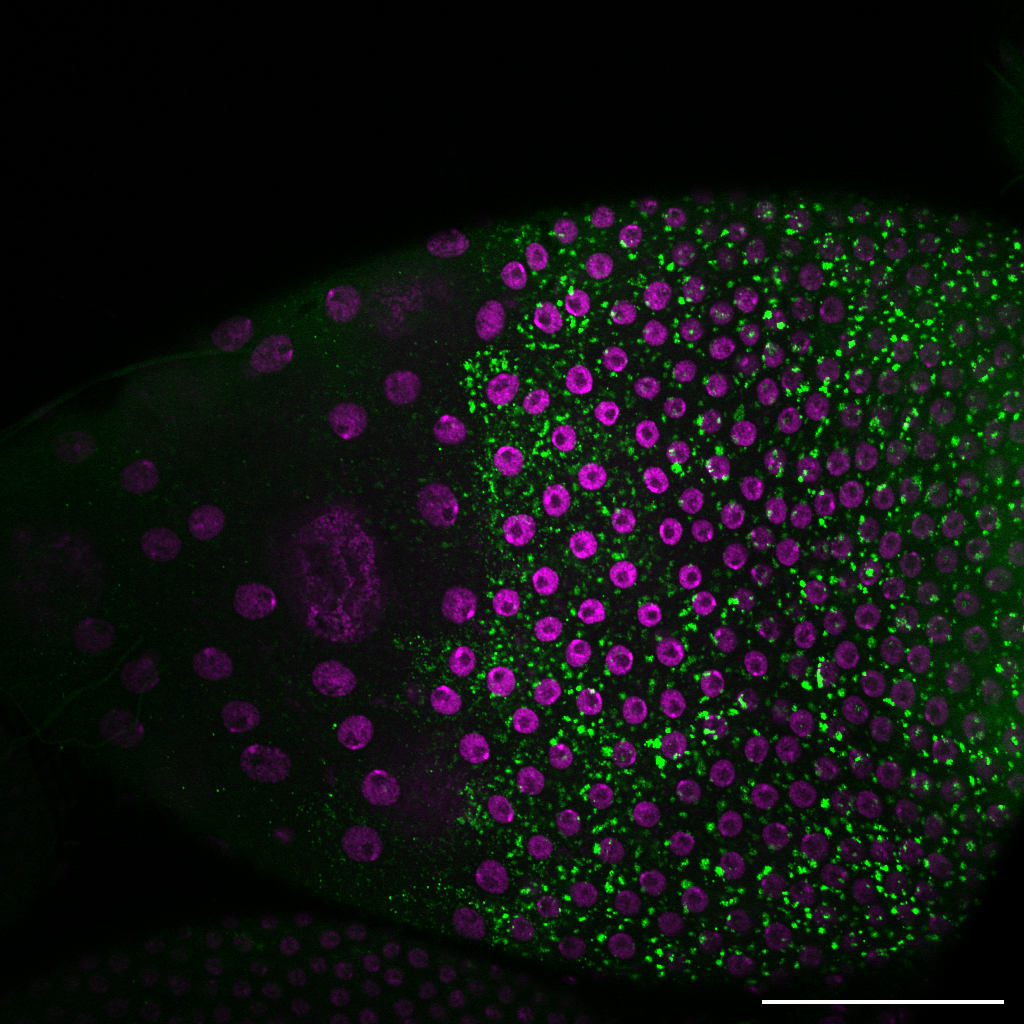

Supplement: Supplementary file 14 — Figure EV5 Source Data [file 44319_2025_672_MOESM14_ESM.zip › EV5/C/79021-stage9-sc-1.tif]

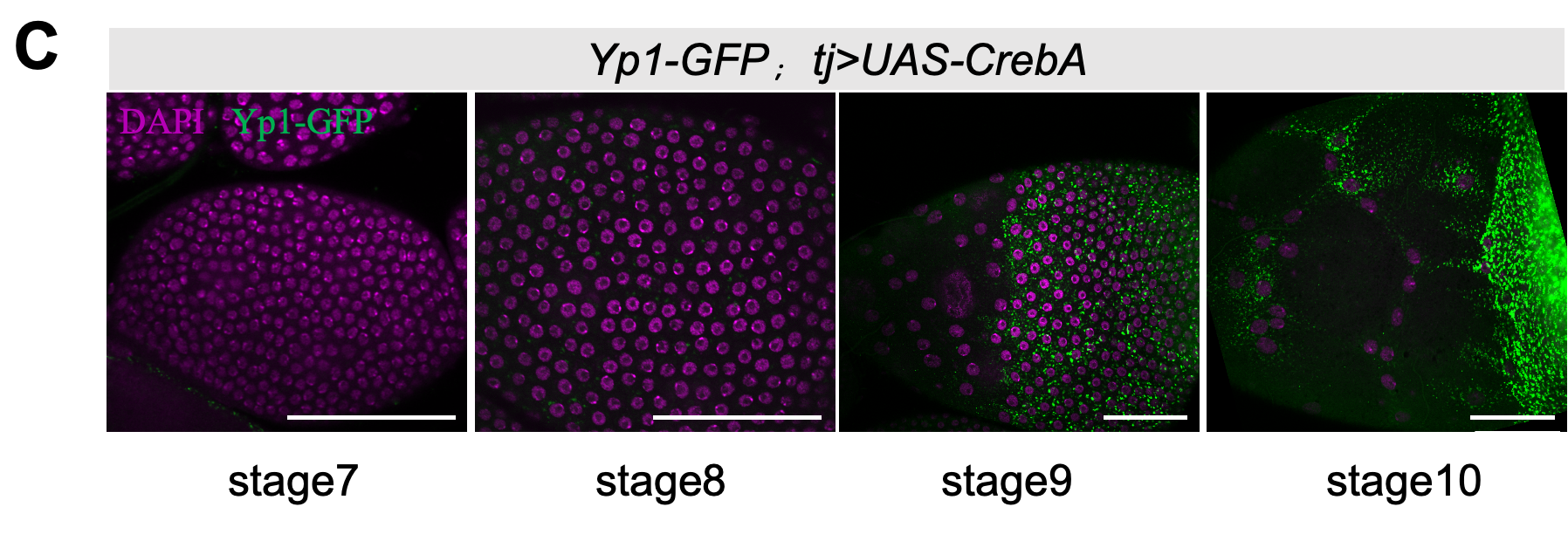

Supplement: Supplementary file 14 — Figure EV5 Source Data [file 44319_2025_672_MOESM14_ESM.zip › EV5/C/C.tif]
