# Supplementary material for: An insulin receptor activity surge in follicle cells drives vitellogenesis by upregulating CrebA
Source: EMBO Rep. 2026 Jan 3;27(3):748–73. doi: 10.1038/s44319-025-00672-6 (PMC12894986; doi:10.1038/s44319-025-00672-6)
Supplement: Supplementary file 15 — Expanded View Figures [file 44319_2025_672_MOESM15_ESM.pdf]

Expanded View Figures

Figure EV1. InR activity in stretched cells and centripetal cells.

(**A**) Quantification of InR activity in stretched cells (blue rectangle) at stage 9. $N = 6$ egg chambers. The quantification was performed using the same method as in Fig. 1B. Error bars represent mean ± SEM. Scale bar = 100 µm. (**B**) Quantification of InR activity in stretched cells at stage 10. $N = 5$ egg chambers. Error bars represent mean ± SEM. Scale bar = 100 µm. (**C**) For centripetal cells, the quantification was performed within the border region of a *z*-stacked image (a total of 33 slices and the interval between neighboring slices = 2 µm). $N = 7$ egg chambers. Error bars represent mean ± SEM. Scale bar = 100 µm. We speculate that the interiorly situated cells might have an insufficient accessibility to insulin-like peptides, a possibility worthy for future investigation. Source data are available online for this figure.

 © The Author(s)

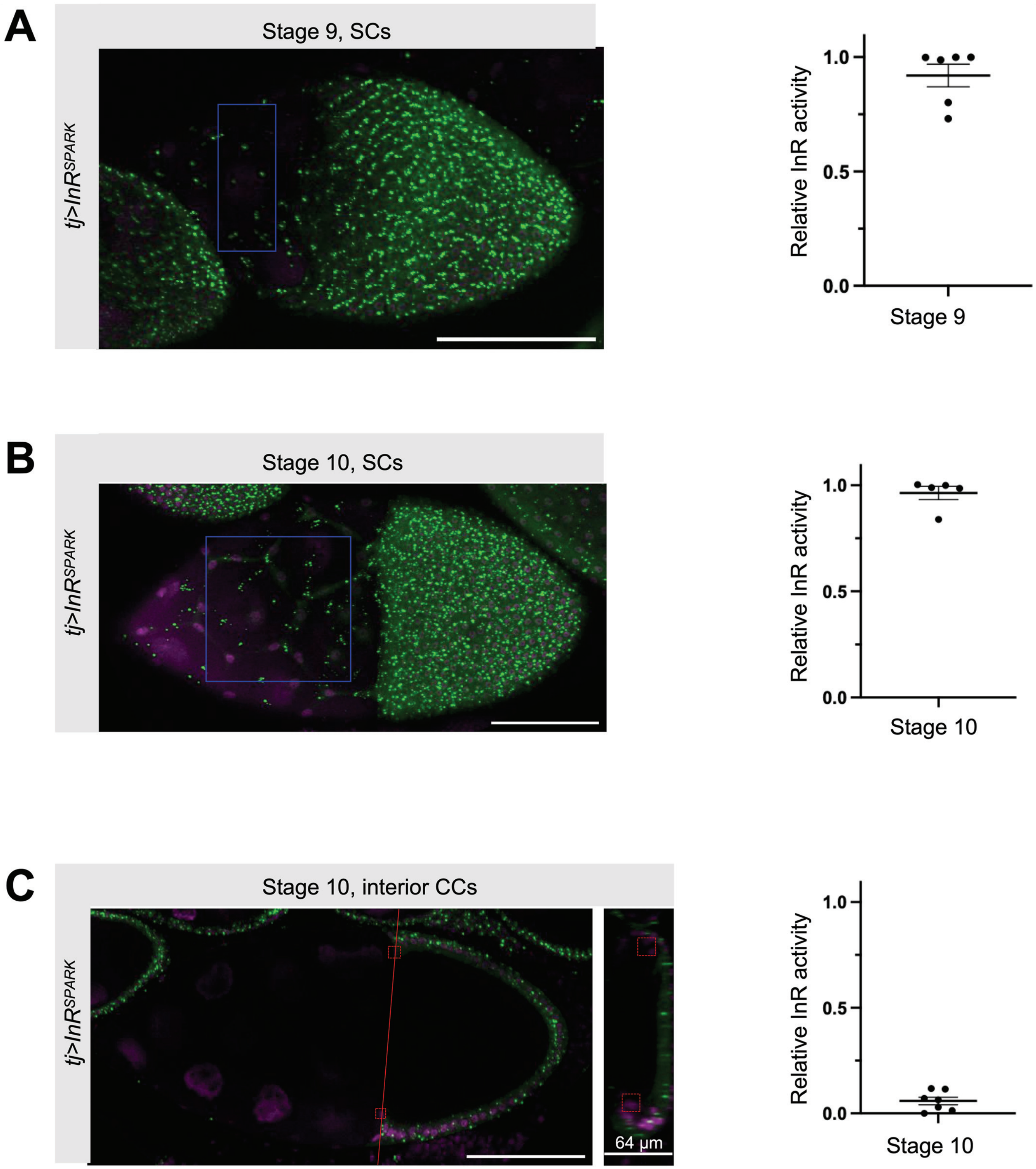
A
Stage 9, SCs
tj>InRSPARK
Relative InR activity
1.0
0.5
0.0
Stage 9
B
Stage 10, SCs
tj>InRSPARK
Relative InR activity
1.0
0.5
0.0
Stage 10
C
Stage 10, interior CCs
tj>InRSPARK
64 µm
Relative InR activity
1.0
0.5
0.0
Stage 10

© The Author(s)

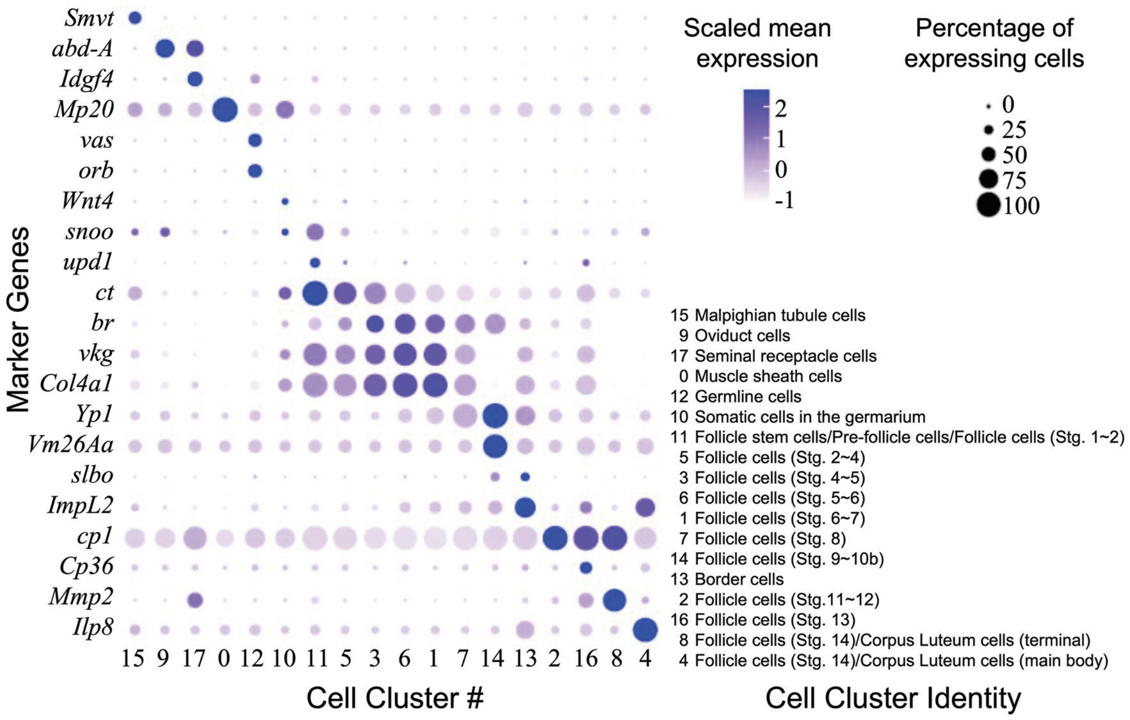

Figure EV2. Marker genes to annotate cell clusters from scRNA-seq data.

A dotplot of the scaled expression of marker genes in each inferred cell type. The size of each dot represents the percentage of cells in a given cluster expressing a given gene, and the color of each dot represents a *z*-score-scaled value of the gene's average expression across all cells in the cluster. Source data are available online for this figure.

 © The Author(s)

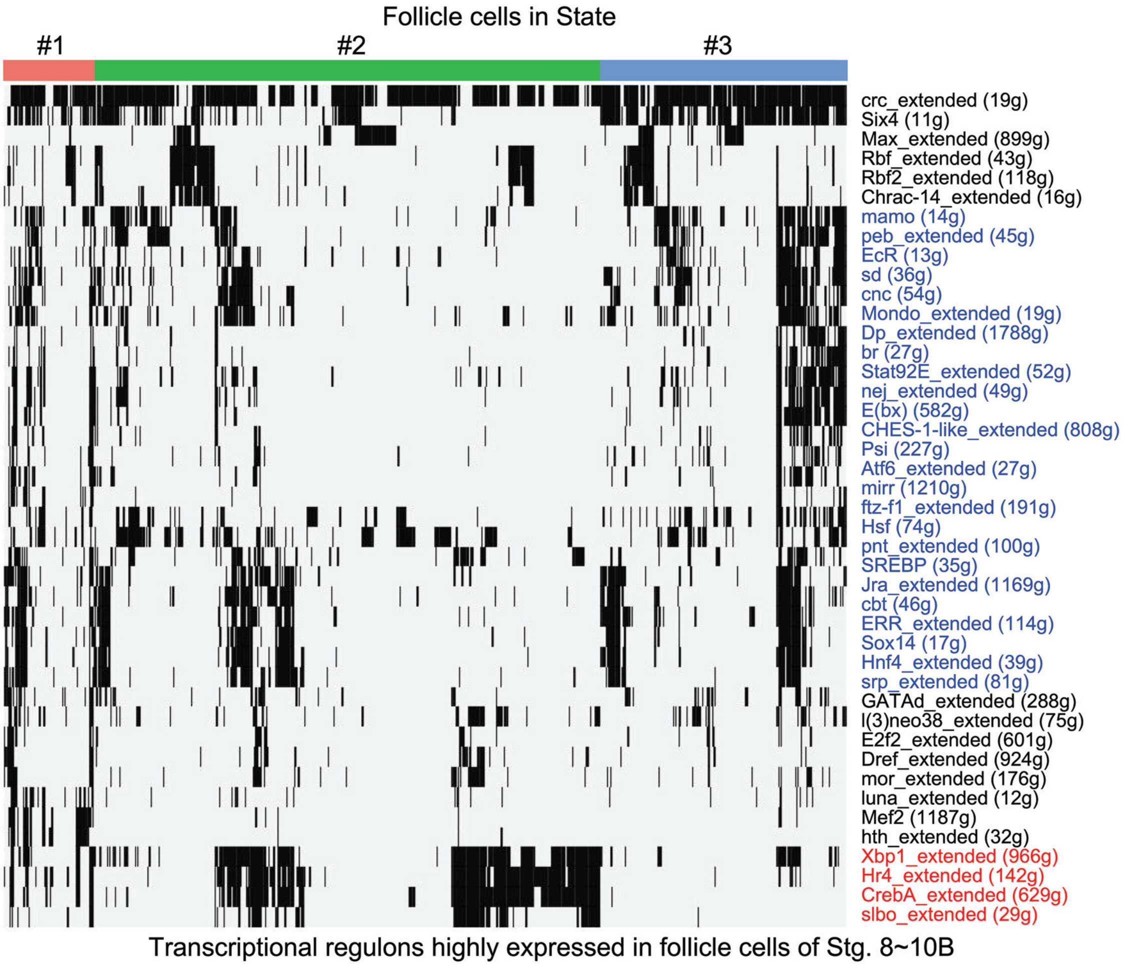

Figure EV3. Active transcriptional regulons in vitellogenic follicle cells.

SCENIC (Van de Sande et al, 2020) was used to infer the single-cell transcription regulatory network. This figure plots the binary activity scores of all the 42 regulons identified from follicle cells at stages 8–10b. A black bar indicates an active state of the given regulon in the given cell. Grouped by hierarchical clustering, 24 regulons have elevated activities in the HSD-induced population #3 (blue), and 4 regulons show deactivation in this population (red). Source data are available online for this figure.

A

Top 10 GO terms enriched with CrebA-regulon genes

GO term	Count	Benjamini
COPII-coated ER to Golgi transport vesicle	12	2.3E-08
membrane	184	6.9E-07
COPII vesicle coat	6	3.8E-05
regulation of lipid storage	9	8.7E-04
signal peptidase complex	4	6.8E-03
positive regulation of cell migration	7	1.0E-02
SRP-dependent cotranslational protein targeting to membrane, translocation	5	1.0E-02
positive regulation of transcription by RNA polymerase II	33	1.2E-02
vitelline membrane formation involved in chorion-containing eggshell formation	6	1.5E-02
oligosaccharyltransferase complex	5	1.8E-02

B

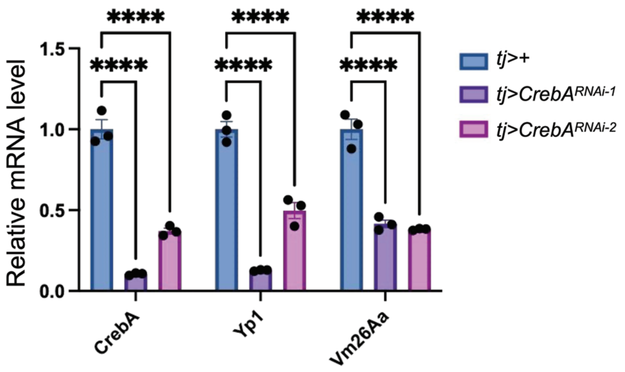

Figure EV4. CrebA is required for ovarian transcription of yolk protein and vitelline membrane genes.

(**A**) Top ten GO terms enriched with CrebA-regulon genes. (**B**) RT-qPCR analysis of the ovarian mRNA levels of *CrebA*, *Yp1*, and *Vm26Aa* in *tj> CrebA*$^{RNAi-1}$ and *tj> CrebA*$^{RNAi-2}$ females. For each genotype, seven pairs of ovaries were sampled. Data were presented as mean ± SEM (N = 3 biological replicates for each measurement). Error bars represent mean ± SEM. One-way ANOVA was performed to compare either RNAi line with the control line, and all p values <0.0001 (denoted as ****). Source data are available online for this figure.

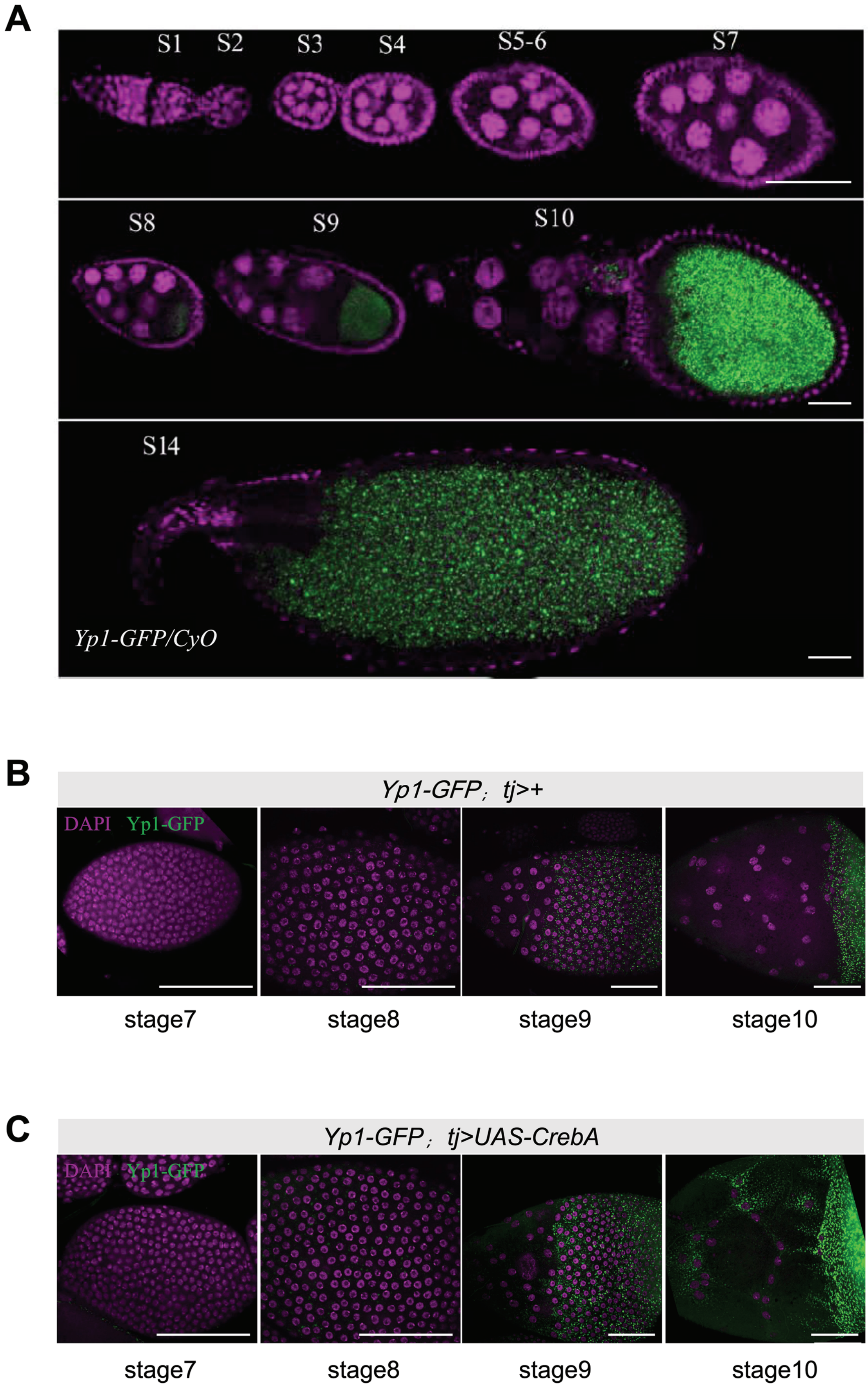
A
S1
S2
S3
S4
S5-6
S7
S8
S9
S10
S14
Yp1-GFP/CyO
B
Yp1-GFP; tj>+
DAPI Yp1-GFP
stage7
stage8
stage9
stage10
C
Yp1-GFP; tj>UAS-CrebA
DAPI Yp1-GFP
stage7
stage8
stage9
stage10

Figure EV5. CrebA can ectopically activate the expression of Yp1-GFP in follicle cells.

(**A**) Representative images of Yp1-GFP (green) in egg chambers at different stages from *Yp1-GFP/CyO* females. Nuclei were counterstained with DAPI (magenta). Scale bars = 50 µm. (**B**) Representative images of Yp1-GFP in egg chambers at different stages from *Yp1-GFP/tj-Gal4;UAS-CrebA/+* females. Scale bars = 50 µm. (**C**) Representative images of Yp1-GFP in egg chambers at different stages from *Yp1-GFP/tj-Gal4;UAS-CrebA/+* females. Scale bars = 50 µm. We note that, in stretched cells where both CrebA and Yp1-GFP are normally low, ectopic expression of CrebA significantly increased Yp1-GFP. Source data are available online for this figure.

 © The Author(s)
